# Association of omega 3 polyunsaturated fatty acids with incident chronic kidney disease: pooled analysis of 19 cohorts

**DOI:** 10.1136/bmj-2022-072909

**Published:** 2023-01-18

**Authors:** Kwok Leung Ong, Matti Marklund, Liping Huang, Kerry-Anne Rye, Nicholas Hui, Xiong-Fei Pan, Casey M Rebholz, Hyunju Kim, Lyn M Steffen, Anniek C van Westing, Johanna M Geleijnse, Ellen K Hoogeveen, Yun-Yu Chen, Kuo-Liong Chien, Amanda M Fretts, Rozenn N Lemaitre, Fumiaki Imamura, Nita G Forouhi, Nicholas J Wareham, Anna Birukov, Susanne Jäger, Olga Kuxhaus, Matthias B Schulze, Vanessa Derenji de Mello, Jaakko Tuomilehto, Matti Uusitupa, Jaana Lindström, Nathan Tintle, William S Harris, Keisuke Yamasaki, Yoichiro Hirakawa, Toshiharu Ninomiya, Toshiko Tanaka, Luigi Ferrucci, Stefania Bandinelli, Jyrki K Virtanen, Ari Voutilainen, Tharusha Jayasena, Anbupalam Thalamuthu, Anne Poljak, Sonia Bustamante, Perminder S Sachdev, Mackenzie K Senn, Stephen S Rich, Michael Y Tsai, Alexis C Wood, Markku Laakso, Maria Lankinen, Xiaowei Yang, Liang Sun, Huaixing Li, Xu Lin, Christoph Nowak, Johan Ärnlöv, Ulf Risérus, Lars Lind, Mélanie Le Goff, Cécilia Samieri, Catherine Helmer, Frank Qian, Renata Micha, Adrienne Tin, Anna Köttgen, Ian H de Boer, David S Siscovick, Dariush Mozaffarian, Jason HY Wu

**Affiliations:** 1 Lipid Research Group, School of Biomedical Sciences, University of New South Wales, Sydney, NSW, Australia; 2 The George Institute for Global Health, Faculty of Medicine and Health, University of New South Wales, Sydney, NSW, Australia; 3 The Welch Center for Prevention, Epidemiology, and Clinical Research, Johns Hopkins Bloomberg School of Public Health, Baltimore, MD, USA; 4 Department of Public Health and Caring Sciences, Clinical Nutrition and Metabolism, Uppsala University, Uppsala, Sweden; 5 Key Laboratory of Birth Defects and Related Diseases of Women and Children (Sichuan University), Ministry of Education, West China Second University Hospital, Sichuan University, Chengdu, Sichuan, China; 6 Shuangliu Institute of Women's and Children's Health, Shuangliu Maternal and Child Health Hospital, Chengdu, Sichuan, China; 7 Department of Epidemiology, Johns Hopkins Bloomberg School of Public Health, Baltimore, MD, USA; 8 University of Minnesota School of Public Health, Minneapolis, MN, USA; 9 Division of Human Nutrition and Health, Wageningen University, Wageningen, The Netherlands; 10 Department of Nephrology, Jeroen Bosch Hospital, Den Bosch, The Netherlands; 11 Institute of Epidemiology and Preventive Medicine College of Public Health, National Taiwan University, Taipei, Taiwan; 12 Department of Medical Research, Taichung Veterans General Hospital, Taichung, Taiwan; 13 Heart Rhythm Center, Division of Cardiology, Department of Medicine, Taipei Veterans General Hospital, Taipei, Taiwan; 14 Department of Epidemiology, University of Washington, Seattle, WA, USA; 15 Department of Medicine, University of Washington, Seattle, WA, USA; 16 MRC Epidemiology Unit, University of Cambridge School of Clinical Medicine, Cambridge, UK; 17 Department of Molecular Epidemiology, German Institute of Human Nutrition Potsdam-Rehbruecke, Nuthetal, Germany; 18 German Center for Diabetes Research (DZD), München-Neuherberg, Germany; 19 Institute of Nutritional Science, University of Potsdam, Potsdam, Germany; 20 Institute of Public Health and Clinical Nutrition, School of Medicine, University of Eastern Finland, Kuopio, Finland; 21 Population Health Unit, Finnish Institute for Health and Welfare, Helsinki, Finland; 22 Department of Public Health, University of Helsinki, Helsinki, Finland; 23 Saudi Diabetes Research Group, King Abdulaziz University, Jeddah, Saudi Arabia; 24 The Fatty Acid Research Institute, Sioux Falls, SD, USA; 25 Department of Population Health Nursing Science, College of Nursing, University of Illinois—Chicago, Chicago, IL, USA; 26 Department of Internal Medicine, Sanford School of Medicine, University of South Dakota, Sioux Falls, SD, USA; 27 Department of Epidemiology and Public Health, Graduate School of Medical Sciences, Kyushu University, Fukuoka, Japan; 28 Department of Medicine and Clinical Science, Graduate School of Medical Sciences, Kyushu University, Fukuoka, Japan; 29 Translational Gerontology Branch, National Institute on Aging, National Institute of Health, Baltimore, MD, USA; 30 Geriatric Unit, Azienda USL Toscana Centro, Florence, Italy; 31 Centre for Healthy Brain Ageing, School of Psychiatry, University of New South Wales, Sydney, NSW, Australia; 32 Mark Wainwright Analytical Centre, University of New South Wales, Sydney, NSW, Australia; 33 USDA/ARS Children’s Nutrition Research Center, Department of Pediatrics, Baylor College of Medicine, Houston, TX, USA; 34 Department of Public Health Sciences, University of Virginia, Charlottesville, VA, USA; 35 Center for Public Health Genomics, University of Virginia, Charlottesville, VA, USA; 36 Department of Laboratory Medicine and Pathology, University of Minnesota, Minneapolis, MN, USA; 37 Institute of Clinical Medicine, Internal Medicine, University of Eastern Finland, Kuopio, Finland; 38 Department of Medicine, Kuopio University Hospital, Kuopio, Finland; 39 Shanghai Institute of Nutrition and Health, University of Chinese Academy of Sciences, Chinese Academy of Sciences, Shanghai, China; 40 Key Laboratory of Systems Health Science of Zhejiang Province, Hangzhou Institute for Advanced Study, University of Chinese Academy of Sciences, Chinese Academy of Sciences, Hangzhou, China.; 41 Department of Neurobiology, Care Sciences and Society, Karolinska Institutet, Sweden; 42 Department of Medical Sciences, Uppsala University, Uppsala, Sweden; 43 Bordeaux Population Health Research Centre, INSERM, UMR 1219, University of Bordeaux, Bordeaux, France; 44 Department of Nutrition, Harvard T.H. Chan School of Public Health, Harvard Medical School, Boston, MA, USA; 45 Department of Medicine, Beth Israel Deaconess Medical Center, Harvard Medical School, Boston, MA, USA; 46 Department of Food Science and Nutrition, University of Thessaly, Karditsa, Greece; 47 The Friedman School of Nutrition Science and Policy, Tufts University, Boston, MA, USA; 48 Department of Medicine, University of Mississippi Medical Center, Jackson, MS, USA; 49 Institute of Genetic Epidemiology, Department of Data Driven Medicine, Faculty of Medicine and Medical Centre, University of Freiburg, Freiburg, Germany; 50 Division of Nephrology, Department of Medicine, University of Washington, Seattle, WA, USA; 51 Kidney Research Institute, University of Washington, Seattle, WA, USA; 52 Puget Sound VA Healthcare System, Seattle, WA, USA; 53 New York Academy of Medicine, New York, NY, USA; 54 School of Population Health, University of New South Wales, Sydney, NSW, Australia

## Abstract

**Objective:**

To assess the prospective associations of circulating levels of omega 3 polyunsaturated fatty acid (n-3 PUFA) biomarkers (including plant derived α linolenic acid and seafood derived eicosapentaenoic acid, docosapentaenoic acid, and docosahexaenoic acid) with incident chronic kidney disease (CKD).

**Design:**

Pooled analysis.

**Data sources:**

A consortium of 19 studies from 12 countries identified up to May 2020.

**Study selection:**

Prospective studies with measured n-3 PUFA biomarker data and incident CKD based on estimated glomerular filtration rate.

**Data extraction and synthesis:**

Each participating cohort conducted de novo analysis with prespecified and consistent exposures, outcomes, covariates, and models. The results were pooled across cohorts using inverse variance weighted meta-analysis.

**Main outcome measures:**

Primary outcome of incident CKD was defined as new onset estimated glomerular filtration rate <60 mL/min/1.73 m^2^. In a sensitivity analysis, incident CKD was defined as new onset estimated glomerular filtration rate <60 mL/min/1.73 m^2^ and <75% of baseline rate.

**Results:**

25 570 participants were included in the primary outcome analysis and 4944 (19.3%) developed incident CKD during follow-up (weighted median 11.3 years). In multivariable adjusted models, higher levels of total seafood n-3 PUFAs were associated with a lower incident CKD risk (relative risk per interquintile range 0.92, 95% confidence interval 0.86 to 0.98; P=0.009, I^2^=9.9%). In categorical analyses, participants with total seafood n-3 PUFA level in the highest fifth had 13% lower risk of incident CKD compared with those in the lowest fifth (0.87, 0.80 to 0.96; P=0.005, I^2^=0.0%). Plant derived α linolenic acid levels were not associated with incident CKD (1.00, 0.94 to 1.06; P=0.94, I^2^=5.8%). Similar results were obtained in the sensitivity analysis. The association appeared consistent across subgroups by age (≥60 *v* <60 years), estimated glomerular filtration rate (60-89 *v* ≥90 mL/min/1.73 m^2^), hypertension, diabetes, and coronary heart disease at baseline.

**Conclusions:**

Higher seafood derived n-3 PUFA levels were associated with lower risk of incident CKD, although this association was not found for plant derived n-3 PUFAs. These results support a favourable role for seafood derived n-3 PUFAs in preventing CKD.

## Introduction

Chronic kidney disease (CKD) affects about 700 million people worldwide, with an estimated global prevalence of one in 11 in the general population.^1^ ^2^ Patients with CKD are at higher risk of cardiovascular disease and death^3^ ^4^ because the condition could eventually progress to kidney failure that severely impacts health and quality of life.^5^ ^6^ Therefore, there is a need to identify factors that might prevent the onset and progression of CKD.

The consumption of long chain omega 3 polyunsaturated fatty acids (n-3 PUFAs) that are mostly obtained from seafood (including eicosapentaenoic acid (EPA), docosapentaenoic acid (DPA), and docosahexaenoic acid (DHA)) confers cardiometabolic benefits.^7^ Meta-analyses of randomised controlled trials have shown that increased n-3 PUFA intake improved arterial stiffness,^8^ lowered blood pressure,^9^ and reduced plasma triglycerides.^10^ ^11^ Because endothelial dysfunction, hypertension, and dyslipidaemia are CKD risk factors,^12^ ^13^ ^14^ n-3 PUFAs could protect against the development of CKD.

However, there are important limitations to the observational research addressing this important theory. Firstly, most previous studies assessed self-reported intake of n-3 PUFAs using dietary questionnaires and evaluated associations with CKD in cross sectional analyses, with inconsistent associations with prevalent CKD.^15^ ^16^ ^17^ ^18^ Only two longitudinal studies assessed self-reported intake of n-3 PUFAs and risk of incident CKD, and also reported inconsistent findings.^18^ ^19^ Secondly, the use of self-reported n-3 PUFA intake is subject to errors related to misreporting and inaccuracy of food composition databases.^20^ Circulating n-3 PUFA levels are valid and reliable biomarkers of intake as shown by several randomised controlled trials.^20^ ^21^ ^22^ ^23^ ^24^ Moreover, biomarkers reflect the dietary intake and also the underlying metabolism, and therefore indicate the bioavailable n-3 PUFA intake; this might explain why n-3 PUFA biomarkers have been reported to be superior to self-reported n-3 PUFA intake in predicting outcomes such as cardiovascular disease and all cause mortality.^25^ Thirdly, self-reported intake could not distinguish accurately between individual n-3 PUFAs, which might differ in their biological properties and effects on CKD.^26^ Finally, studies evaluating the association between α linolenic acid (ALA) biomarker level and incident CKD are lacking, even though ALA is the major dietary n-3 PUFA obtained from specific plant foods and oils that has been associated with lower cardiovascular outcome risk.^27^ ^28^

We pooled cohort studies conducted by different research teams to assess the association of n-3 PUFA biomarkers with incident CKD. This strategy allowed us to more precisely quantify the association with a larger sample size, distinguish between individual n-3 PUFAs, and avoid measurement errors related to self-reported dietary intake.

## Methods

### Study population

The study was conducted within the Fatty Acids and Outcomes Research Consortium (FORCE) (http://force.nutrition.tufts.edu), a consortium of studies with circulating or adipose tissue fatty acid biomarker measurements and ascertained chronic disease events.^28^ ^29^ ^30^ ^31^ ^32^ Studies were identified and study investigators were asked to participate based on previous FORCE projects, expert contacts, and literature searches. All prospective studies of adult participants with n-3 PUFA biomarkers measured in blood fractions or adipose tissue and with data on the primary outcome of incident CKD were eligible. A total of 50 potentially relevant studies were identified in May 2020, but 22 (44%) were not eligible because of lack of exposure or outcome data, and 5 (10%) studies were not included because of a limited number of patients with incident CKD (supplementary table S1). Of the remaining 23 cohorts, 19 (83%) participated in this project, while 4 (17%) declined. The study proposal and analytical plan were approved by FORCE, and all participating cohorts have ethics approval with appropriate data sharing agreements, which enabled the current project to be performed. Detailed cohort information is provided in the supplementary methods.

### Measurement of n-3 PUFA biomarkers

Biomarker levels of n-3 PUFAs (ALA, EPA, DPA, and DHA) were measured by each cohort in plasma phospholipids, erythrocyte phospholipids, total plasma, total serum, cholesterol esters, or several lipid compartments using gas chromatography (supplementary methods). Individual fatty acid levels were expressed as the proportion of total fatty acids.

### Outcome measurement

All outcomes were determined using estimated glomerular filtration rate (eGFR) at baseline and follow-up visits, with the same definitions in all the 19 participating cohorts. The primary outcome was incident CKD using the same definition as used by the Chronic Kidney Disease Genetics (CKDGen) Consortium.^33^ Briefly, incident CKD was defined as an eGFR that decreased to <60 mL/min/1.73 m^2^ during follow-up among participants with baseline eGFR≥60 mL/min/1.73 m^2^. eGFR was estimated using the 2009 Chronic Kidney Disease Epidemiology Collaboration equation with calibrated serum or plasma creatinine.^34^ If creatinine levels were not calibrated and were obtained with a Jaffé assay before 2009, then data were corrected by multiplying creatinine levels by 0.95 before eGFR calculation, as described previously.^35^ A more stringent definition of incident CKD was used in a sensitivity analysis: both eGFR<60 mL/min/1.73 m^2^ and <75% of baseline eGFR during follow-up among participants with baseline eGFR≥60 mL/min/1.73 m^2^.^36^

We also assessed two secondary outcomes: annual absolute change in eGFR from baseline to the last follow-up as a continuous variable; and ≥40% decrease in eGFR from baseline to the last follow-up.^37^ Unlike the primary outcomes, analysis of the secondary outcomes comprised all participants, including those with baseline eGFR<60 mL/min/1.73 m^2^.

### Cohort analyses

A prespecified and harmonised protocol with the same participant inclusion and exclusion criteria, exposures, outcomes, covariates, and analytical methods were sent to all cohorts to perform de novo analyses of individual data. For the outcomes of incident CKD and ≥40% decrease in eGFR, Cox proportional hazards models were used to estimate hazard ratios, considered to approximate relative risk, for cohorts with eGFR available in two or more follow-up visits. In cohorts with data available for only one follow-up eGFR, Poisson regression models were used to estimate relative risks. For the outcome of annual absolute change in eGFR from baseline to the last follow-up, multivariable linear regression was used. All analyses estimated robust standard errors.

Because n-3 PUFA biomarker levels were measured in different lipid compartments in the participating cohorts, they were evaluated as continuous variables using study specific interquintile range (difference between midpoint of lowest fifth (10th centile) and highest fifth (90th centile)).^29^ ^30^ ^31^ ^32^ To assess potential nonlinear associations, n-3 PUFA levels were also evaluated as variables (in fifths) using study specific cut-off points. In the primary multivariable model (model 1), analyses were adjusted for age, sex, race, clinical centre or field site, education, occupation, body mass index, smoking, alcohol intake, physical activity, prevalent coronary heart disease, and use of lipid lowering drugs at baseline, when applicable. In model 2, additional covariates that could confound or mediate the association of n-3 PUFAs with incident CKD were considered, including prevalent diabetes mellitus, urine albumin-creatinine ratio, systolic blood pressure, and use of antihypertensive drugs at baseline (see supplementary methods for definitions of all covariates). Participants with missing data on n-3 PUFAs were excluded from the analysis. Missing data on covariates were handled according to the usual practice of each cohort and study investigators (see supplementary methods for cohort specific details).

In each study, a statistical interaction was evaluated by fitting the n-3 PUFA biomarker (continuous), each potential effect modifier, and their cross product. The prespecified effect modifiers included age (≥60 *v* <60 years), race, hypertension, diabetes, coronary heart disease, and eGFR (60-89 *v* ≥90 mL/min/1.73 m^2^) at baseline. These modifiers were selected by considering their potential biological relevance.

### Data pooling and meta-analysis

Effect estimates from each participating cohort were provided to the lead author in standardised electronic forms and pooled using inverse variance weighted meta-analysis. Results from participating cohorts were pooled overall and within each specific type of lipid compartment. For studies with measures in multiple compartments, we prioritised the compartments in the overall pooled analysis on the basis of which best reflect long term intake, as prespecified in the following order: erythrocyte phospholipids, plasma phospholipids, cholesterol esters, and total plasma or serum.^22^ In sensitivity analyses, the overall pooled analyses were performed after excluding data from one cohort at a time or using alternative biomarker compartments. To assess potential nonlinear relations, exploratory analysis was conducted to model the association within each compartment using meta-regression with restricted cubic splines constructed from variables (in fifths) using study specific cut-off points.^28^ ^29^ ^30^ ^31^ ^32^ ^38^ Interaction terms were pooled using inverse variance weighted meta-regression with Bonferroni correction for multiple testing, and such analysis was considered exploratory. All meta-analyses were performed using Stata (version 16.0) or R (version 4.1.0), with two tailed P<0.05 considered statistically significant.

### Patient and public involvement

Patients and members of the public were not directly involved in this research owing to lack of funding, staff, and infrastructure to facilitate their involvement in this pooled analysis of data from 19 participating cohorts.

## Results

### Study cohorts

The primary outcome analysis included 25 570 participants without prevalent CKD from 19 cohorts in a total of 12 countries (table 1). Among the 19 cohorts, the mean age of participants ranged from 49 to 77 years and the mean body mass index from 23.2 to 28.3. The weighted median follow-up duration was 11.3 years. Sixteen cohorts recruited men and women, and most (n=15) recruited predominantly white participants (supplementary table S2). The mean baseline eGFR ranged from 76.1 to 99.8 mL/min/1.73 m^2^ (supplementary table S3), and the mean annual change in eGFR ranged from −6.2 to 0.3 mL/min/1.73 m^2^/year. In total, 4944 participants (19.3%) developed the primary outcome of incident CKD during follow-up (supplementary table S4).

**Table 1:** Baseline characteristics of 19 prospective cohorts that participated in analysis of association of n-3 PUFA biomarkers with incident chronic kidney disease (as part of FORCE Consortium)

Study	Country	Study design	Sample size (*n*)	Median follow-up (years)	Age, years (mean±SD)	Sex (% female)	BMI (mean±SD)	Biomarker measurement	
								Lipid compartment	Year of sampling
AOC	Netherlands	Cohort of patients with myocardial infarction	2026	3.4	68.9±5.4	20.6	27.7±3.6	Cholesterol esters	2002-2006
ARIC	USA	Population based cohort of middle aged adults	3526	25.8	53.9±5.6	47.7	27.0±4.6	Plasma phospholipids	1987-1989
CCCC	Taiwan	Population based cohort	1074	1.0	56.8±9.7	42.1	23.9±3.2	Total serum	1990-1991
CHS	USA	Population based cohort of older adults	1608	5.6	73.3±4.2	63.0	26.7±4.5	Plasma phospholipids	1992-1993
EPIC-Norfolk	United Kingdom	Population based cohort	926	13.8	59.2±7.5	50.2	26.0±3.5	Plasma phospholipids	1993–1997
EPIC-Potsdam	Germany	Population based cohort	253	19.6	49.0±7.9	56.1	25.3±3.4	Erythrocyte phospholipids	2008
FDPS	Finland	Randomised trial cohort of patients with impaired glucose tolerance at baseline	355	4.0	55.1±7.2	67.5	31.0±4.7	Total serum	1993-1998
FHS	USA	Population based cohort	1895	5.8	54.1±8.1	50.3	28.3±5.4	Erythrocyte phospholipids	2008
Hisayama	Japan	Population based cohort	2713	10.0	60.2±11.4	58.4	23.2±3.4	Total serum	2002-2003
InCHIANTI	Italy	Population based cohort	830	9.1	64.7±15.3	52.2	27.1±0.3	Total plasma	1998-2000
KIHD	Finland	Population based cohort of men	1082	20.0	52.0±5.2	0.0	26.7±3.2	Total serum	1991
MAS	Australia	Population based cohort of older adults without dementia at baseline	395	6.0	77.0±4.3	51.9	27.1±4.2	Total plasma	2005-2006
MESA	USA	Population based cohort	1682	9.5	58.7±9.1	53.3	27.8±5.4	Plasma phospholipids	2000-2002
METSIM	Finland	Population based cohort of men	1152	4.8	55.0±5.6	0.0	26.3±3.4	Erythrocyte phospholipids, plasma phospholipids, cholesterol esters	2006-2010
NHAPC	China	Population based cohort	2046	6.0	58.0±5.9	57.6	24.5±3.6	Erythrocyte phospholipids	2005
PIVUS	Sweden	Population based cohort of older adults	893	9.9	70.2±0.2	47.6	26.9±4.1	Plasma phospholipids	2001
ULSAM	Sweden	Population based cohort of men	1055	21.3	49.6±0.6	0.0	25.0±4.4	Cholesterol esters	1970-1973
WHIMS	USA	Randomised trial cohort of older postmenopausal women	1686	15.3	69.2±3.4	100.0	28.3±5.5	Erythrocyte phospholipids	1995
3C	France	Population based cohort	373	4.2	76.1±4.6	62.7	26.1±4.0	Erythrocyte phospholipids, total plasma*	1999-2001

AOC=Alpha Omega Cohort; ARIC=Atherosclerosis Risk in Communities Study; BMI=body mass index; CCCC=Chin-Shan Community Cardiovascular Cohort Study; CHS=Cardiovascular Health Study; EPIC-Norfolk=European Prospective Investigation into Cancer and Nutrition (Norfolk); EPIC-Potsdam=European Prospective Investigation into Cancer and Nutrition (Potsdam); FDPS=Finnish Diabetes Prevention Study; FHS=Framingham Heart Study; FORCE=Fatty Acids and Outcomes Research Consortium; InCHIANTI=Invecchiare in Chianti Study; KIHD=Kuopio Ischaemic Heart Disease Risk Factor Study; MAS=Memory and Ageing Study; MESA=Multi-Ethnic Study of Atherosclerosis; METSIM=Metabolic Syndrome in Men; n-3 PUFA=omega 3 polyunsaturated fatty acid; NHAPC=Nutrition and Health of Ageing Populations in China; PIVUS=Prospective Investigation of the Vasculature in Uppsala Seniors; SD=standard deviation; ULSAM=Uppsala Longitudinal Study of Adult Men; WHIMS=Women’s Health Initiative Memory Study; 3C=Three City Study.

* Data shown are for 373 participants with n-3 PUFA measurement in erythrocyte phospholipids, which were used in overall pooled analysis. Total plasma n-3 PUFA biomarkers were also measured in larger sample of 1019 participants.

Most of the participating cohorts measured fatty acid levels in erythrocyte or plasma phospholipids (n=11), followed by total plasma or serum (n=7), and cholesterol esters (n=3; table 1). The proportion of total fatty acids for each n-3 PUFA varied between different lipid compartments (supplementary table S5; supplementary fig S1).

### Seafood n-3 PUFAs and incident CKD

**Fig 1:**
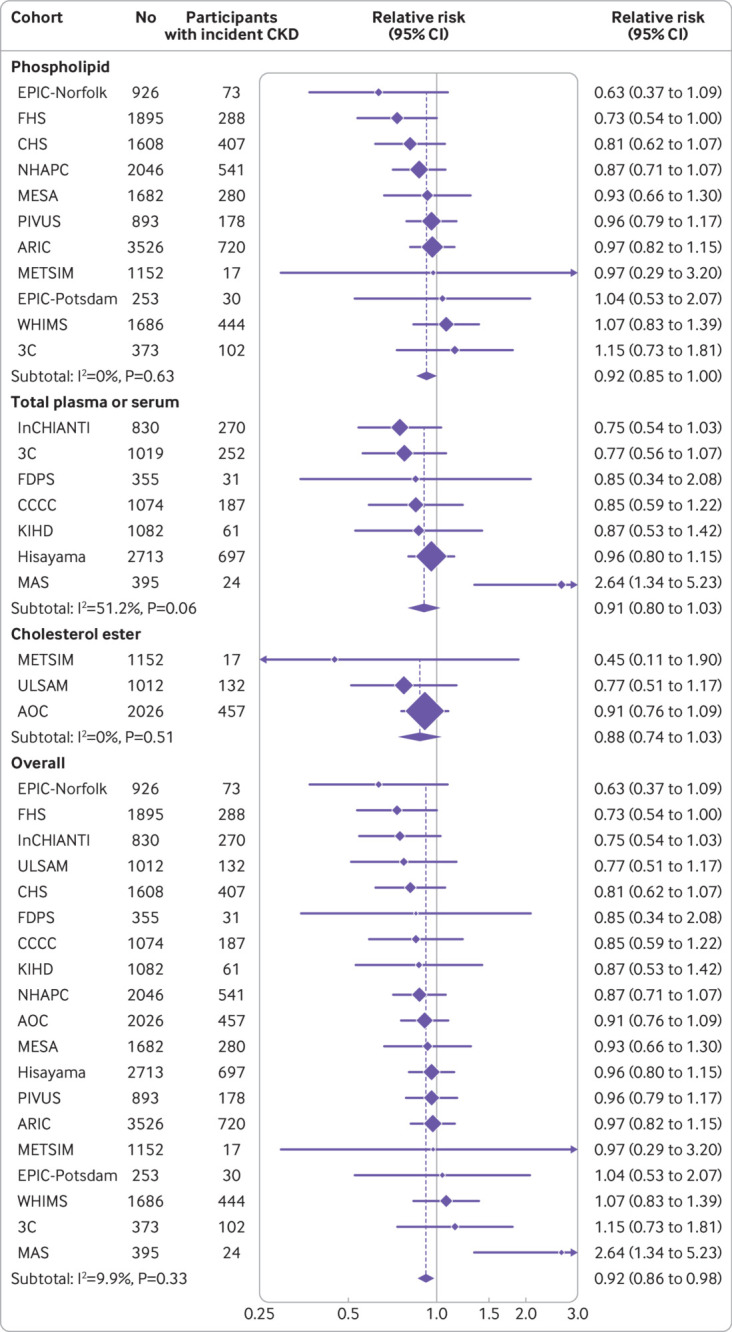
Association of total seafood n-3 PUFAs (eicosapentaenoic acid, docosapentaenoic acid, and docosahexaenoic acid) with incident CKD. Incident CKD was defined as an eGFR<60 mL/min/1.73 m^2^ during follow-up among participants with baseline eGFR≥60 mL/min/1.73 m^2^. Analyses were adjusted for age, sex, race, field centre if applicable, education, occupation, body mass index, smoking, physical activity, alcohol intake, prevalent coronary heart disease, and use of lipid lowering drugs. Study specific estimates per interquintile range (difference between midpoint of lowest fifth and highest fifth) of total n-3 PUFAs were pooled separately for different lipid compartments and overall. AOC=Alpha Omega Cohort; ARIC=Atherosclerosis Risk in Communities Study; CCCC=Chin-Shan Community Cardiovascular Cohort Study; CHS=Cardiovascular Health Study; CKD=chronic kidney disease; eGFR=estimated glomerular filtration rate; EPIC-Norfolk=European Prospective Investigation into Cancer and Nutrition (Norfolk); EPIC-Potsdam=European Prospective Investigation into Cancer and Nutrition (Potsdam); FDPS=Finnish Diabetes Prevention Study; FHS=Framingham Heart Study; InCHIANTI=Invecchiare in Chianti Study; KIHD=Kuopio Ischaemic Heart Disease Risk Factor Study; MAS=Memory and Ageing Study; MESA=Multi-Ethnic Study of Atherosclerosis; METSIM=Metabolic Syndrome in Men; n-3 PUFA=omega 3 polyunsaturated fatty acid; NHAPC=Nutrition and Health of Ageing Populations in China; PIVUS=Prospective Investigation of the Vasculature in Uppsala Seniors; WHIMS=Women’s Health Initiative Memory Study; ULSAM=Uppsala Longitudinal Study of Adult Men; 3C=Three City Study

Higher levels of total seafood derived n-3 PUFAs were associated with 8% lower risk of incident CKD per interquintile range with low heterogeneity in the observed associations between cohorts (relative risk 0.92, 95% confidence interval 0.86 to 0.98; P=0.009, I^2^=9.9%; table 2 and fig 1). Findings were not appreciably altered by further adjustment for covariates that could be potential confounders or mediators (0.91, 0.85 to 0.97; P=0.006, I^2^=17.2%). Similar results were obtained when the n-3 PUFAs were assessed categorically—participants with total seafood n-3 PUFA level in the highest fifth had 13% lower risk of incident CKD than those in the lowest fifth (0.87, 0.80 to 0.96; P=0.005, I^2^=0.0%; table 2 and supplementary fig S2). Protective associations were found for the individual seafood n-3 PUFAs (table 2), and associations appeared consistent across lipid compartments (fig 1; supplementary table S6). Little evidence could be found of nonlinear associations between total seafood n-3 PUFAs and incident CKD in any of the biomarker compartments (supplementary fig S3).

**Table 2:** Association of seafood n-3 PUFA biomarkers with primary outcome of incident CKD

n-3 PUFA biomarker (No of studies; participants with incident CKD)*	Model†	Per interquintile range			Highest fifth *v* lowest fifth	
		Relative risk (95% CI)	I^2^ (%)		Relative risk (95% CI)	I^2^ (%)
EPA (19; 4940)	1	0.94 (0.89 to 1.00)	0.0		0.92 (0.84 to 1.00)	0.0
	2	0.94 (0.88 to 0.99)	0.0		0.91 (0.83 to 0.99)	7.0
DPA (16; 4350)	1	0.94 (0.88 to 1.00)	0.0		0.89 (0.81 to 0.98)	0.0
	2	0.94 (0.80 to 1.01)	0.0		0.90 (0.82 to 0.99)	0.0
DHA (19; 4944)	1	0.93 (0.87 to 1.00)	27.0		0.89 (0.81 to 0.97)	0.0
	2	0.93 (0.87 to 0.99)	30.1		0.89 (0.81 to 0.97)	8.4
EPA+DPA+DHA‡ (19; 4939)	1	0.92 (0.86 to 0.98)	9.9		0.87 (0.80 to 0.96)	0.0
	2	0.91 (0.85 to 0.97)	17.2		0.88 (0.80 to 0.96)	0.0
ALA (19; 4940)	1	1.00 (0.94 to 1.06)	5.8		0.98 (0.89 to 1.07)	0.0
	2	0.99 (0.93 to 1.05)	0.0		0.97 (0.88 to 1.06)	0.0

Effect estimates were pooled using inverse variance weighted meta-analysis.

ALA=α linolenic acid; CKD=chronic kidney disease; DHA=docosahexaenoic acid; DPA=docosapentaenoic acid; EPA=eicosapentaenoic acid; n-3 PUFA=omega 3 polyunsaturated fatty acid.

* Small difference in number of participants with incident CKD was due to missing measurement for specific n-3 PUFA fatty acids in some cohorts.

† Model 1 adjusted for age, sex, race, clinical centre or field site, education, occupation, body mass index, smoking, alcohol intake, physical activity, prevalent coronary heart disease, and use of lipid lowering drugs, when applicable. Model 2 adjusted for all covariates in model 1 and also adjusted for prevalent diabetes mellitus, urine albumin-creatinine ratio, systolic blood pressure, use of angiotensin converting enzyme inhibitors or angiotensin receptor blockers, and use of other antihypertensive drugs.

‡ DPA was not available in three of the cohorts (CCCC—Chin-Shan Community Cardiovascular Cohort, InCHIANTI—Invecchiare in Chianti Study, and ULSAM—Uppsala Longitudinal Study of Adult Men), therefore the sum in these cohorts was calculated as EPA+DHA.

When incident CKD was defined using a more stringent definition (new onset of eGFR<60 mL/min/1.73 m^2^ and <75% of baseline eGFR) in a sensitivity analysis, the results were largely consistent with the primary outcome analysis. Higher levels of total seafood n-3 PUFAs were associated with a lower risk of incident CKD (0.91, 0.84 to 0.99 per interquintile range; P=0.009, I^2^=27.3%; supplementary table S7). In additional sensitivity analyses, the association of total seafood n-3 PUFAs with incident CKD remained significant after excluding data from one cohort at a time, or using alternative biomarker compartments in the overall pooled results (supplementary table S8).

### Seafood n-3 PUFAs and secondary outcomes

Total seafood n-3 PUFA level was not associated with the outcome of ≥40% decrease in eGFR in continuous analyses, but was associated with a lower risk (about 15%) for those in the highest fifth versus those in the lowest fifth (relative risk 0.85, 95% confidence interval 0.74 to 0.98; P=0.03, I^2^=44.1%; table 3).

**Table 3:** Association of seafood n-3 PUFA biomarkers with secondary outcome of ≥40% decrease in eGFR from baseline

n-3 PUFA biomarker (No of studies; participants with ≥40% decrease in eGFR)*	Model†	Per interquintile range				Highest fifth *v* lowest fifth	
		Relative risk (95% CI)	I^2^ (%)			Relative risk (95% CI)	I^2^ (%)
EPA (16; 2554)	1	0.99 (0.91 to 1.07)	0.0			0.85 (0.74 to 0.98)	19.5
	2	0.98 (0.91 to 1.06)	7.6			0.84 (0.73 to 0.97)	26.1
DPA (13; 2110))	1	0.92 (0.82 to 1.02)	42.5			0.85 (0.73 to 0.98)	21.8
	2	0.92 (0.82 to 1.03)	42.1			0.86 (0.74 to 1.00)	33.7
DHA (16; 2554)	1	0.93 (0.84 to 1.03)	52.2			0.88 (0.76 to 1.01)	46.2
	2	0.93 (0.83 to 1.03)	54.3			0.88 (0.76 to 1.02)	47.1
EPA+DPA+DHA‡ (16; 2552)	1	0.95 (0.86 to 1.04)	45.2			0.85 (0.74 to 0.98)	44.1
	2	0.94 (0.85 to 1.03)	46.4			0.85 (0.74 to 0.98)	45.3
ALA (16; 2553)	1	0.98 (0.89 to 1.07)	35.8			0.97 (0.85 to 1.12)	7.1
	2	0.94 (0.86 to 1.04)	18.9			0.95 (0.82 to 1.09)	0.0

Effect estimates were pooled using inverse variance weighted meta-analysis.

ALA=α linolenic acid; CKD=chronic kidney disease; DHA=docosahexaenoic acid; DPA=docosapentaenoic acid; eGFR=estimated glomerular filtration rate; EPA=eicosapentaenoic acid; n-3 PUFA=omega 3 polyunsaturated fatty acid.

* Number of cohorts contributing to this analysis was lower than the primary outcome because three cohorts were excluded due to low number of participants with ≥40% decrease in eGFR (FDPS—Finnish Diabetes Prevention Study, MAS—Memory and Ageing Study, and METSIM—Metabolic Syndrome in Men). The small difference in number of participants with ≥40% decrease in eGFR was due to missing measurement for specific n-3 PUFA fatty acids in some cohorts.

† Model 1 adjusted for age, sex, race, clinical centre or field site, education, occupation, body mass index, smoking, alcohol intake, physical activity, prevalent coronary heart disease, and use of lipid lowering drugs, when applicable. Model 2 adjusted for all covariates in model 1 and also adjusted for prevalent diabetes mellitus, urine albumin-creatinine ratio, systolic blood pressure, use of angiotensin converting enzyme inhibitors or angiotensin receptor blockers, and use of other antihypertensive drugs.

‡ DPA was not available in three of the cohorts (CCCC—Chin-Shan Community Cardiovascular Cohort, InCHIANTI—Invecchiare in Chianti Study, and ULSAM—Uppsala Longitudinal Study of Adult Men), therefore the sum in these cohorts was calculated as EPA+DHA.

Higher levels of total seafood n-3 PUFAs, especially DHA, were associated with a slower annual decline in eGFR (table 4). For instance, the annual decline in eGFR was 0.07 mL/min/1.73 m^2^ lower (95% confidence interval 0.02 to 0.13; P=0.007, I^2^=42.2%) for people with total seafood n-3 PUFA level in the highest fifth than those in the lowest fifth.

**Table 4:** Association of seafood n-3 PUFA biomarkers with secondary outcome of annual change in eGFR

n-3 PUFA biomarker (No of studies; total participants)*	Model†	Per interquintile range			Highest fifth *v* lowest fifth	
		Mean difference (95% CI)	I^2^ (%)		Mean difference (95% CI)	I^2^ (%)
EPA (19; 28 804)	1	0.02 (−0.01 to 0.05)	26.6		0.03 (−0.02 to 0.09)	37.2
	2	0.02 (−0.01 to 0.05)	28.3		0.04 (−0.01 to 0.09)	37.0
DPA (16; 25 102)	1	0.03 (−0.02 to 0.07)	46.4		0.03 (−0.03 to 0.09)	7.8
	2	0.02 (−0.02 to 0.06)	38.8		0.02 (−0.04 to 0.08)	2.6
DHA (19; 28 837)	1	0.05 (0.01 to 0.09)	36.6		0.08 (0.02 to 0.13)	32.9
	2	0.05 (0.01 to 0.08)	33.0		0.07 (0.01 to 0.12)	27.9
EPA+DPA+DHA‡ (19; 28 798)	1	0.04 (0.01 to 0.08)	32.0		0.07 (0.02 to 0.13)	42.2
	2	0.04 (0.00 to 0.07)	29.8		0.07 (0.01 to 0.12)	36.2
ALA (19; 28 826)	1	−0.03 (−0.06 to 0.00)	24.1		−0.05 (−0.10 to 0.01)	15.1
	2	−0.03 (−0.06 to 0.01)	20.3		−0.05 (−0.10 to 0.01)	3.4

Data shown are adjusted mean difference (95% CI) in the change of eGFR (mL/min/1.73 m^2^) per year. Effect estimates were pooled using inverse variance weighted meta-analysis.

ALA=α linolenic acid; DHA=docosahexaenoic acid; DPA=docosapentaenoic acid; eGFR=estimated glomerular filtration rate; EPA=eicosapentaenoic acid; n-3 PUFA=omega 3 polyunsaturated fatty acid.

* The small difference in number of participants with data on annual change in eGFR was due to missing measurement for specific n-3 PUFAs in some cohorts.

† Model 1 adjusted for age, sex, race, clinical centre or field site, education, occupation, body mass index, smoking, alcohol intake, physical activity, prevalent coronary heart disease, and use of lipid lowering drugs, when applicable. Model 2 adjusted for all covariates in model 1 and also adjusted for prevalent diabetes mellitus, urine albumin-creatinine ratio, systolic blood pressure, use of angiotensin converting enzyme inhibitors or angiotensin receptor blockers, and use of other antihypertensive drugs.

‡ DPA was not available in three of the cohorts (CCCC—Chin-Shan Community Cardiovascular Cohort, InCHIANTI—Invecchiare in Chianti Study, and ULSAM—Uppsala Longitudinal Study of Adult Men), therefore the sum in these cohorts was calculated as EPA+DHA.

### ALA and incident CKD

ALA was not associated with incident CKD, with low heterogeneity in the observed associations between cohorts (relative risk 1.00 per interquintile range, 95% confidence interval 0.94 to 1.06; P=0.94, I^2^=5.8%; table 2 and supplementary fig S4). No significant association was observed in analyses using a more stringent definition of incident CKD (supplementary table S7). No significant association was observed in the secondary outcomes of ≥40% decrease in eGFR (0.98, 0.89 to 1.07; P=0.08, I^2^=35.8%; table 3) and annual change in eGFR (mean difference −0.03 mL/min/1.73 m^2^ per interquintile range, 95% confidence interval −0.06 to 0.00; P=0.16, I^2^=24.1%; table 4).

### Effect modification analyses

The association of n-3 PUFAs with the primary outcome of incident CKD was not modified by key demographic and clinical characteristics at baseline, including age, race, hypertension, diabetes, coronary heart disease, and eGFR (P for pooled interaction terms >0.05). No difference in association was observed based on study location, follow-up duration, and sample size (supplementary tables S9-11).

## Discussion

### Principal findings

This de novo pooled analysis of 19 cohort studies gathered data from more than 25 000 patients as part of the FORCE consortium. We found that higher levels of seafood n-3 PUFA biomarkers were associated with a modestly lower risk of incident CKD and slower decline in renal function, whereas these associations were not found with higher levels of plant n-3 PUFAs (ALA). The results were consistent across a range of sensitivity analyses, highlighting the robustness of the findings. Similar trends were observed using secondary outcomes of >40% decrease in eGFR and annual changes in eGFR. These findings support a favourable role for increased intake of seafood n-3 PUFAs for the primary prevention of CKD.

### Comparison with previous studies

Randomised controlled trials found increased intake of seafood n-3 PUFAs reduced blood pressure, a key risk factor for CKD development.^9^ Conversely, ALA generally exhibits weaker to no effect on metabolic risk factors such as lipid, glucose, and inflammatory profile in randomised controlled trials compared with EPA and DHA.^39^ Furthermore, findings from previous experimental animal studies provide strong biological plausibility similar to our results of the beneficial effects of seafood n-3 PUFAs on CKD.^40^ ^41^ ^42^ For example, in a mouse model of nephrectomy induced chronic renal failure, supplementation with EPA and DHA for 12 weeks mitigated tubulointerstitial injury through reductions in fibrosis, inflammation, and oxidative stress.^40^ Increased endogenous production of DHA by a transgenic technique also protected against the development of kidney fibrosis and inflammation in mice with unilateral ureter obstruction induced nephropathy.^41^ Renoprotective mechanisms might be mediated through the endogenous conversion of n-3 PUFAs to specialised inflammation resolving mediators such as resolvins.^43^ ^44^ Most previous experiments focused on EPA and DHA, but less is known about DPA, although limited evidence also suggests DPA has favourable properties on cardiometabolic profile.^45^ ^46^

Although our findings do not prove a causal relation between seafood n-3 PUFAs and CKD risk, they are supportive and consistent with current clinical guidelines that recommend adequate intake of seafood as part of healthy dietary patterns, especially when seafood replaces the intake of less healthy foods.^47^ ^48^ ^49^ Although ALA could be enzymatically transformed to the longer chain n-3 PUFAs like EPA and DHA, such endogenous conversion only occurs at a low rate^27^ and our findings suggest intake of this plant derived n-3 PUFA alone might not maintain renal health. However, although ALA is an essential fatty acid, circulating ALA generally has a weaker correlation with ALA intake compared with the seafood derived n-3 PUFAs,^22^ ^50^ ^51^ potentially explained by a higher oxidation rate of ALA compared with other fatty acids.^52^ Therefore, if ALA intake were associated with incident CKD, we might not be able to detect such an association using an ALA biomarker. In our study, the levels of EPA and DHA probably mostly reflected variations in dietary intake from seafood, rather than n-3 PUFA supplements, because relatively small proportions of the general population used n-3 PUFA supplements, especially for studies that were conducted before the early 2000s when fish oil supplements were infrequently used (most of the included cohorts).^53^ Current dietary guidelines generally recommend at least two servings per week of oily fish to provide around 250 mg/day of long chain n-3 PUFAs for the general population.^27^ ^49^

Given the renoprotective effects of seafood n-3 PUFAs observed in experimental animal studies, human trials have also assessed the use of n-3 PUFA supplementation to prevent decline in kidney function among patients with existing CKD or kidney failure. A recent systematic review of randomised controlled trials found n-3 PUFA supplementation could improve lipid profile and reduce oxidative stress, but not blood pressure in patients with CKD.^54^ Another recent systematic review of randomised controlled trials found n-3 PUFA supplementation could prevent the progression to kidney failure in patients with CKD who were not receiving renal replacement therapy, although the certainty of evidence is low.^55^ Therefore, current clinical guidelines do not recommend the use of n-3 PUFA supplementation to prevent further decline in renal function for those with existing CKD, although using n-3 PUFA supplementation to treat hypertriglyceridaemia is recommended.^56^ Our findings highlight the need for large randomised controlled trials to assess increased intake of seafood n-3 PUFAs for the primary prevention of CKD and decline in renal function. One such randomised controlled trial of 1312 patients with type 2 diabetes found that fish oil supplementation (daily dose of 1 g, containing 465 mg EPA and 375 mg DHA) did not affect the change in eGFR over five years.^57^ Several other randomised controlled trials have also reported null or small beneficial effects of n-3 PUFA supplementation on kidney function, but these were secondary outcomes or post hoc analyses and were not powered to detect incident CKD as a primary outcome.^58^ ^59^ Further, randomised controlled trials of n-3 PUFA supplementation often included participants with sufficient seafood intake, in which additional n-3 PUFA supplementation might not confer additional cardiometabolic benefits.^60^ Therefore, future randomised controlled trials could target increased seafood intake, instead of n-3 PUFA supplementation, because consumption of seafood can replace the intake of less healthy foods. Finally, all these trials tested n-3 PUFA supplementation mainly in high risk populations, limiting the applicability of the study findings to the general population.

### Strengths and limitations of this study

Our study has several strengths. Our literature search only identified two high quality prospective observational analyses of the association of n-3 PUFA biomarkers with incident CKD or decline in kidney. These two studies were included in our pooled analysis with updated patient numbers and harmonised analytical protocols.^61^ ^62^ Our findings expand upon these previous individual cohort based analyses with a sevenfold larger sample size and enhanced statistical power to more precisely quantify the association of n-3 PUFAs with CKD. The large sample size also enabled a detailed evaluation of potential effect modifiers of the association between n-3 PUFAs and CKD. By collaborating with participating cohorts using a standardised approach to define exposures, covariates, and outcome variables, we reduced potential heterogeneity that is inherent in a publication based meta-analysis. Our collaborative approach enabled the inclusion of most of the cohorts with available exposure and outcome data, which reduced the likelihood of publication bias.

The use of n-3 PUFA biomarkers allowed us to distinguish between individual n-3 PUFAs that could have differing biological effects,^26^ and avoid measurement errors related to self-reported dietary intake. Conducting analyses across cohorts from diverse demographic, dietary, and medical backgrounds with different incident CKD rates enhanced the generalisability of our findings, and the use of several related secondary outcomes and different sensitivity analyses enhanced the robustness of the findings. However, more studies are needed to further understand the generalisability of our findings, especially in countries experiencing high incidence of CKD such as China and India.

Our study also has some limitations. The n-3 PUFA biomarkers and covariates were only measured once at baseline and this might lead to increasing random misclassification over time, which would tend to bias the associations towards the null. However, n-3 PUFA biomarkers have good long term reproducibility as determined in a previous study with repeated within person measurements across 13 years.^63^ While we standardised our methods across cohorts as much as possible, fatty acid assays, determination of outcomes, and covariate measurements varied by cohort and might contribute to the heterogeneity in our findings. Among different prespecified covariates, many participating cohorts did not have data for urine albumin-creatinine ratio. However, in cohorts that measured urine albumin-creatinine ratio, association of n-3 PUFA levels with incident CKD was similar whether it was adjusted for or not (results not shown). Although, the possibility of residual confounding by other unmeasured or imprecisely measured covariates could not be excluded, the differential association of plant derived ALA and seafood n-3 PUFAs with incident CKD suggests residual confounding was unlikely to entirely account for our findings. In this analysis, data were not adjusted for n-6 PUFA biomarker levels because experimental evidence on their relation with kidney function was limited. Because n-6 PUFA levels are associated with a lower CVD risk,^30^ future studies assessing their association with incident CKD are warranted, pending more experimental work assessing their possible impact on CKD.

### Conclusion

In this large de novo pooled analysis across 19 cohorts with more than 25 000 patients, higher seafood n-3 PUFA levels were associated with a lower incident CKD risk and a slower decline in renal function. Although the magnitude of these associations was modest, our findings suggest adequate consumption of seafood and oily fish should be part of healthy dietary patterns. Additionally, further randomised controlled trials are warranted to assess the potential beneficial role of seafood n-3 PUFAs in preventing and managing CKD.

**What is already known on this topic**

Animal studies suggest omega 3 polyunsaturated fatty acids (n-3 PUFAs) have beneficial effects on kidney function, but evidence from human studies is limited
Most previous studies on chronic kidney disease (CKD) assessed self-reported intake of n-3 PUFAs using dietary questionnaires, which are subject to errors related to misreporting and inaccuracy of food composition databases

**What this study adds**

Higher seafood n-3 PUFA levels (eicosapentaenoic acid, docosapentaenoic acid, and docosahexaenoic acid) were associated with a lower incident CKD risk and a slower decline in renal function
Plant derived n-3 PUFA level (α linolenic acid) was not associated with incident CKD
Findings are consistent with dietary guidelines recommending seafood and oily fish consumption as part of healthy dietary patterns, and provide strong evidence for further trials analysing seafood derived n-3 PUFAs in CKD prevention

## Data Availability

Individual participant data are owned by individual participating cohorts and are available to researchers on consent from participating cohorts. For further queries or requests, please contact force@tufts.edu. Further details are available at the FORCE website: http://force.nutrition.tufts.edu.

## Web extra

Extra material supplied by authors

Web appendix: Supplementary online content

## Notes

Contributors: KLO and JHYW designed the study and drafted the manuscript. KLO, MM, KAR, NH, XFP, RM, AT, AK, IHdB, DSS, DM, and JHY contributed to the development of study proposal and protocol. MM, CMR, HK, LMS, ACvW, JMG, EKH, YYC, KLC, AMF, RNL, FI, NGF, NJW, AB, SJ, OK, MBS, VDdM, JT, MU, JL, NT, WSH, KY, YH, TN, TT, LF, SBa, JKV, AV, TJ, AT, AP, SBu, PSS, MKS, SSR, MYT, ACW, MLaa, MLan, XY, LS, HL, XL, CN, JA, UR, LL, MLG, CS, and CH contributed to cohort specific data (principal investigators, analysts, associates, and trainees). KLO and LH performed the pooled analysis of cohort data. KLO, MM, KAR, XFP, WSH, FQ, AT, AK, IhdB, DSS, DM, and JHYW contributed to the primary writing team. All authors contributed to the data interpretation, critically revised the manuscript and approved the final version for publication. KLO is the guarantor of this manuscript and accepts full responsibility for the work and the conduct of the study, had access to the data, and controlled the decision to publish. The corresponding author attests that all listed authors meet authorship criteria and that no others meeting the criteria have been omitted.

The lead author (KLO) affirms that the manuscript is an honest, accurate, and transparent account of the study being reported; that no important aspects of the study have been omitted; and that any discrepancies from the study as originally planned in the protocol have been explained.

Dissemination to participants and related patient and public communities: We plan to share the results of this study through press releases, presentations, institutional newsletters, and multiple social media platforms, including Twitter and LinkedIn.

Provenance and peer review: Not commissioned; externally peer reviewed.
