# Supplementary material for: Association of omega 3 polyunsaturated fatty acids with incident chronic kidney disease: pooled analysis of 19 cohorts
Source: BMJ. 2023 Jan 18;380:e072909. doi: 10.1136/bmj-2022-072909 (PMC9846698; doi:10.1136/bmj-2022-072909)

Supplementary Online Content

Ong KL et al. Association of omega-3 polyunsaturated fatty acids with incident chronic kidney disease: a pooled analysis of 19 cohorts

Supplementary Methods. Detailed cohort information and methods

Supplementary Table S1. Invited studies not participating in the analysis

Supplementary Table S2. Distribution of categorical covariates at baseline

Supplementary Table S3. Distribution of continuous covariates at baseline

Supplementary Table S4. Number of incident cases for each of the primary and secondary outcomes across the 19 participating cohorts

Supplementary Table S5. Baseline levels of omega-3 polyunsaturated fatty acid biomarkers

Supplementary Table S6. Association of seafood n-3 PUFA biomarkers measured in different lipid compartments with the primary outcome of incident CKD

Supplementary Table S7. Association of n-3 PUFA biomarkers with incident CKD using a more stringent definition of incident CKD

Supplementary Table S8. Association of seafood n-3 PUFA biomarkers with incident CKD in sensitivity analyses

Supplementary Table S9. Association of n-3 PUFA biomarkers with incident CKD by study location

Supplementary Table S10. Association of n-3 PUFA biomarkers with incident CKD by study follow-up duration

Supplementary Table S11. Association of n-3 PUFA biomarkers with incident CKD by study sample size

Supplementary Figure S1. Levels of n-3 PUFA biomarkers across different compartments in the 19 participating cohorts

Supplementary Figure S2. Association of quintiles of seafood n-3 PUFA biomarkers with incident CKD

Supplementary Figure S3. Relationship between total seafood n-3 PUFA (EPA+DPA+DHA) and risk of incident CKD as assessed using restricted cubic splines

Supplementary Figure S4. Association of α-linolenic acid with incident CKD

Supplementary Methods. Detailed cohort information and methods

1. AOC: Alpha Omega Cohort

General information: AOC is a cohort of 4,837 non-hospitalised patients who experienced a myocardial infarction up to 10 years before enrolment.[1] The study includes a trial phase (Alpha Omega Trial, 3-year intervention with low doses of n-3 fatty acids, until 2009). It is now used as prospective cohort study for risk prediction in post-MI patients. The patients were recruited in collaboration with cardiologists from 32 Dutch hospitals. At baseline (2002-2006), data were collected on diet, lifestyle, cardiovascular risk factors, medical history, and medication use. Subjects were physically examined by trained research nurses, which included anthropometry, blood pressure, heart rate, and blood sampling. Examinations were repeated after 20 months (midterm examination in n=800) and 40 months (final examination). Patients have been continuously followed for cause-specific mortality, also after the trial ended.

Among 4,837 adults, there was 2,407 adults with available data on fatty acid biomarkers and eGFR at baseline and follow-up visits, who were included in the analysis for the secondary outcomes. For primary outcomes of incident chronic kidney disease, after excluding 381 adults with prevalent CKD at baseline, a total of 2,026 adults were eligible for the current analysis.

Measurement of fatty acid biomarker concentrations: Plasma cholesterol esters were measured at the Division of Human Nutrition, Wageningen University, the Netherlands using stored blood samples from 2002-2006. Total lipids were extracted from the plasma samples with an isopropanol/hexane mixture (2:3, v/v) and centrifuged. The upper hexane layer was removed and dried under nitrogen atmosphere. Extracted total lipids from plasma samples were separated into cholesterol esters by solid phase extraction silica columns (Sopachem Isolute 460-0050-B, Sopachem BV, the Netherlands). Fatty acids were trans-esterified into fatty acid methyl esters (using boron trifluoride-methanol) and analysed by gas chromatography. Samples were injected into gas chromatograph with flame-ionisation detector (Agilent, Santa Clara, CA). In total, 38 fatty acids were identified and quantified, ranging from 12:0 to 24:1. Fatty acids were identified by comparing retention times with fatty acid standards, and expressed as weight percentage relative to total fatty acids (% total fatty acids). Intra-assay coefficients of variation (CVs) were 1.0%, 1.5% and 1.4% for ALA, EPA and DHA respectively whereas the corresponding inter-assay CVs were 4.7%, 4.9% and 6.7% respectively.

Physical activity assessment: Physical activity was assessed in METhours/week leisure activities.

Statistical methods for incident CKD analysis: Poisson regression

Missing data in covariates: There were 7% missing data in alcohol consumption, and <1% missing data in body mass index, education, physical activity and systolic blood pressure. Missing data in covariates were imputed using sex-specific means/medians for continuous variables and mode for categorical variables.

Funding support and acknowledgements: The AOC study was supported by the Netherlands Heart Foundation (grant no. 2000T401), US National Institutes of Health (NIH/NHLBI and ODS, grant no. R01HL-076200) and Unilever R&D, Vlaardingen. Anniek van Westing was funded by the Jaap Schouten Foundation (grant no. JSF_SU_10_2018). The authors express their gratitude to the Alpha Omega Trial participants.

2. ARIC: Atherosclerosis Risk in Communities Study

General information: The ARIC study is a prospective population-based cohort study of middle-aged adults (45-64 years of age at baseline) initiated to investigate the etiology of atherosclerosis and its clinical sequelae and variation in cardiovascular risk factors, medical care, and disease by race, sex, place and time.[2] Participants were recruited at four field centres (Forsyth County, NC; Jackson, MS; Washington County, MD; Minneapolis, MN). The cohort consists of 15,792 middle-aged men and women, recruited in 1987-1989 (visit 1). Participants attended follow-up visits in 1990-1992 (visit 2), 1993-1995 (visit 3), 1996-1998 (visit 4), 2011-2013 (visit 5), 2016-2017 (visit 6), visit 7 (2018-2019), and visit 8 (2020).

Among 3,793 adults with fatty acid biomarker measurements, we excluded 136 adults with missing eGFR values at all follow-up visits, 59 adults with implausible total energy intake (<500 kcal or >3500 kcal for women; <700 kcal or >4500 kcal for men), and 43 (1.1%) adults with missing covariates, resulting in a total of 3,555 adults in the analysis for the secondary outcomes. For primary outcomes of incident chronic kidney disease, after excluding 29 adults with prevalent CKD at baseline, a total of 3,526 adults were eligible for the current analysis.

Measurement of fatty acid biomarker concentrations: Fatty acids were measured in EDTA plasma that had been frozen at -70˚C. Fatty acid assays were performed at the Collaborative Studies Clinical Laboratory at Fairview-University Medical Center (Minneapolis, MN) as previously described by Cao et al (*Clin Chem*. 2006;52:2265-72). Lipids were extracted with chloroform/methanol and separated by thin layer chromatography. Fatty acid methyl esters were prepared from the phospholipid fraction and separated by gas chromatography using an HP-5890 gas chromatograph (Hewlett- Packard, Palo Alto, CA) with a 100-m capillary Varian CP7420 column. We identified 29 fatty acids. The concentration of each fatty acid was expressed as to percentage of total fatty acids.

Physical activity assessment: Physical activity was assessed using the Baecke questionnaire to obtain the physical activity index, which incorporated frequency, duration, and intensity of each type of activity on average over the past year.

Statistical methods for incident CKD analysis: Cox regression

Missing data in covariates: As described in General information section above, participants with missing data (1.1%) in covariates were excluded from the analysis.

Funding support and acknowledgements: The ARIC study was supported by the National Heart, Lung, and Blood Institute, National Institutes of Health, Department of Health and Human Services (HHSN268201700001I, HHSN268201700002I, HHSN268201700003I, HHSN268201700004I, and HHSN268201700005I). The content is solely the responsibility of the authors and does not necessarily represent the official views of the National Institutes of Health. The authors express their gratitude to the ARIC participants and study staff.

3. CCCC: Chin-Shan community cardiovascular cohort

General information: The CCCC study is a prospective population-based cohort study of people more than or equal to 35 years old at baseline to evaluate the cardiovascular disease occurrence and related risk factors.[3,4] Participants were recruited at one center during 1990 and 1991 in the Chin-Shan community, New Taipei City in Taiwan, from the samples of community household lists. The cohort consisted of 3,602 non-institutional men and women. A total of 1834 adults with available data on circulating fatty acids and available follow-up person-year data were eligible for the current analysis.

Among 1,834 adults, there was 1,718 adults with available data on fatty acid biomarkers and eGFR at baseline and follow-up visits, who were included in the analysis for the secondary outcomes. For primary outcomes of incident chronic kidney disease, after excluding 644 adults with prevalent CKD at baseline, a total of 1,074 adults were eligible for the current analysis.

Measurement of fatty acid biomarker concentrations: A 10-mL tube of EDTA-anticoagulated blood was collected, refrigerated at the site centres, then the blood was centrifuged at 800 x g for 10 min. The resulting plasma was separated and dispensed into several aliquots and frozen at -70°C for analysis for fatty acid content by the same technician. After thawing, 0.5 mL of plasma was extracted with 0.5 mL methanol, followed by 1.0 mL chloroform under a nitrogen atmosphere for lipid extraction. A 5890 gas chromatograph (Hewlett Packard, Avondale, PA, USA) equipped with a 30m-FFAT WCOT glass capillary column (J & W Scientific, Folsom, CA, USA) and a flame-ionisation detector was performed in separated methyl esters, and the 29 individual fatty acid peaks were ascertained by comparing the retention time of each peak relative to the retention times of synthetic standards of known fatty acid components. The relative amount of each fatty acid (% of total fatty acids) was quantified by integrating the area under the peak, and

dividing the result by the total area for all fatty acids. The inter-assay CVs for ALA, EPA and DHA were 6.5%, 4.0% and 7.5% respectively.

Physical activity assessment: Data for physical activity was collected by Chin-Shan Community Cardiovascular Cohort Questionnaire and was defined as participants who had done exercise (the exercise that can make you sweat / at least lasting for 20 minutes) once per week or had a laborious job.

Statistical methods for incident CKD analysis: Poisson regression

Missing data in covariates: There was no missing data for covariates.

Funding support and acknowledgements: This CCCC study was supported by the grants of the National Science Council and the Ministry of Science and Technology, Taiwan (MOST 106-2314-B-002-158-MY3, NSC 102-2314-B-002-080 -MY2, NSC 100-2314-B-002-113-MY3, NSC 97-2314-B-002-130-MY3), National Taiwan University Hospital (NTUH 106-S3453) and National Taiwan University (NTU-101R7622-3). The content is solely the responsibility of the authors and does not necessarily represent the official views of the funding offices. The authors express their gratitude to the CCCC participants.

4. CHS: Cardiovascular Health Study

General information: The CHS study is a prospective population-based cohort study of people ≥65 years old at baseline initiated to evaluate risk factors for the development and progression of cardiovascular disease.[5] Participants were recruited at four field centers (Forsyth County, NC; Sacramento County, CA; Washington County, MD; Pittsburgh, PA) from random samples of Medicare eligibility lists. The cohort consists of 5,201 non-institutionalised men and women, recruited in 1989-1990, plus an additional 687 black participants recruited in 1992-93.

Among 5,888 adults, there was 2,643 adults with available data on fatty acid biomarkers and eGFR at baseline and follow-up visits, who were included in the analysis for the secondary outcomes. For primary outcomes of incident chronic kidney disease, after excluding 1035 adults with prevalent CKD at baseline, a total of 1,608 adults were eligible for the current analysis.

Measurement of fatty acid biomarker concentrations: Plasma phospholipid fatty acids were measured at the Fred Hutchinson Cancer Research Center (Seattle, WA) using stored blood samples from 1992-1993. Total lipids were extracted from plasma using the methods of Folch et al.[6] A one dimensional thin-layer chromatography was used to separate phospholipids from neutral lipids. Phospholipids fraction was directly trans-esterified using the Lepage and Roy method to prepare fatty acid methyl esters, and individual fatty acid methyl esters were separated using gas chromatography (Agilent 5890 Gas Chromatograph flame ionisation detector, Agilent Technologies, Palo Alto, CA; fused silica capillary column SP-2560 [100m x 0.25mm, 0.2µm], Supelco Belefonte, PA; initial 160 degrees Celsius for 16 min, ramp 3 degrees Celsius/min to 240 degrees Celsius, hold 15 minutes). For this analysis, levels of each individual fatty acid are expressed as a weight percentage of total phospholipid fatty acids analysed. Inter-assay CVs were 2.1% for EPA, 1.5% for DPA, 1.6% for DHA, and 3.1% for ALA.

Physical activity assessment: Physical activity was assessed at baseline using self-reported questionnaire. The total number of blocks walked over one week was used as the main measure of physical activity.

Statistical methods for incident CKD analysis: Semi-parametric Cox regression

Missing data in covariates: There were occasional missing values (<1% for most covariates) and multiple imputation was performed for missing data in covariates.

Funding support and acknowledgements: The CHS study was supported by contracts 75N92021D00006, HHSN268201200036C, HHSN268200800007C, HHSN268201800001C, N01HC55222, N01HC85079, N01HC85080, N01HC85081, N01HC85082, N01HC85083, N01HC85086, and grants U01HL080295, U01HL130114 and from

NHLBI, with additional contribution from the National Institute of Neurological Disorders and Stroke (NINDS). Additional support was provided by R01AG023629 from the National Institute on Aging (NIA). A full list of principal CHS investigators and institutions can be found at CHS-NHLBI.org. The authors express their gratitude to the CHS participants.

5. EPIC-Norfolk: European Prospective Investigation into Cancer and Nutrition (EPIC) study (Norfolk)

General information: The EPIC-Norfolk study (DOI 10.22025/2019.10.105.00004) is a prospective study of 25,639 men and women aged 40-79 years in Norfolk, UK similar in characteristics to UK general population samples, who participated in a baseline survey in 1993-1997.[7,8]

Among 25,639 adults, there were 7,384 adults with available data on fatty acid biomarkers measured in plasma phospholipid. Among them, eGFR at baseline and follow-up visits were available in 1,027 adults. Thus, the EPIC-Norfolk Study analysed 1,027 adults in this current study. For primary outcomes of incident chronic kidney disease, after excluding 101 adults with prevalent CKD at baseline, a total of 926 adults were eligible.

Measurement of fatty acid biomarker concentrations: The original assays on 8,000 samples were carried out in the WHO International Agency for Cancer Research laboratories, Lyon, France. Because of laboratory constraints in Lyon, an additional 2,000 samples were analysed in Quotient Laboratories, UK, using the same methods and quality control standards. Citrated plasma straws were retrieved from liquid nitrogen storage, thawed at room temperature and 20 µg of di-palmitoyl-D31-phosphatidylcholine (Sigma, St. Louis, MO) internal standard was added to each 200 µL plasma sample. Following extraction of total lipids with chloroform/methanol, phospholipids were further purified by adsorption chromatography (LC-Si SPE, Supelco/Sigma, St. Louis, MO), transmethylated to fatty acid methyl esters and extracted with hexane. Analysis was carried out by gas chromatography with flame ionisation detection (220°C) using a 30 m x 0.32 mm x 0.2 µm SP2340 fused silica capillary column (Supelco/Sigma, St. Louis, MO). Carrier gas was Helium at a constant flow of 1.3 mL/min. Samples of 0.5 µl were introduced onto the column via on-column injection. The column was held initially for 1 min at 65°C, then programmed at 5°C/min to 135°C, then at 2°C/min to 200°C, and finally at 10°C/min to 220°C. Run time was 60 min.

Identification of individual fatty acid methyl esters was based on comparison with retention times of authentic standards (Sigma, St. Louis, MO). Plasma concentrations were measured by comparison of peak areas of individual fatty acids with the peak area of the palmitoyl-D31-fatty acid methyl ester internal standard using individual calibration curves for each of the 22 fatty acid methyl esters measured. The chromatographic peak for palmitoyl-D31-fatty acid methyl ester elutes about 1 minute earlier than non-labelled palmitoyl fatty acid methyl ester in a zone free of interference from other peaks. Each chromatogram was integrated automatically and checked for accuracy and specificity by a laboratory technician. The analytical method allowed for the analysis of 22 individual fatty acids with chain lengths between 14 and 22 carbons belonging to 6 classes of fatty acids: saturated even chain, SFA (14:0, 16:0, 18:0); odd chain fatty acids (15:0, 17:0); omega-6 polyunsaturated, n-6 PUFA (18:2n-6, 18:3n-6, 20:2n-6, 20:3n-6, 20:4n-6, 22:4n-6, and 22:5n-6); omega-3 polyunsaturated, n-3 PUFA (18:3n-3, 20:5n-3, 22:5n-3, 22:6n-3); mono-unsaturated, MUFA, (16:1n-7, 18:1n-7cis, 18:1n-9cis, 20:1n-9) and trans-fatty acids (16:1n9trans, 18:1n9trans). Results were reported as µmolar plasma concentrations and mol% for each individual fatty acid, taking into account the large difference in molecular mass between the shortest (myristic acid, 14:0) and longest (docosapentaenoic acid, 22:5n-3) chain fatty acid measured. Analytical quality control was carried out by the daily use of standard quality control plasma samples. The CVs for the major fatty acids were between 3% to 13%.

Citrated plasma straws retrieved from storage were thawed, di-palmitoyl-D31-phosphatidylcholine Sigma) internal standard was added to each plasma sample, total lipids extracted, and purified by adsorption chromatography (LC-Si SPE, Supelco/Sigma). Plasma phospholipids were analysed by gas chromatography. Concentrations were measured by comparison of peak areas of individual FAs with the peak area of the internal standard using individual calibration curves.

Physical activity assessment: Physical activity was assessed at baseline using self-reported leisure and work physical activity questionnaire.

Statistical methods for incident CKD analysis: Poisson regression

Missing data in covariates: Among 926 participants with available data on the fatty acid biomarkers and CKD incidence over the follow-up, 23 (2.5%) participants recorded missing information on one or more covariates needed in the most adjusted models. Missing information was imputed with chained equations based on regression models including all the relevant variables. Ten imputed datasets were generated, and a single imputed dataset was used for this project after confirming little variability in results from ten imputed datasets.

Funding support and acknowledgements: The EPIC-Norfolk study has received funding from the Medical Research Council (MR/N003284/1 MC-UU_12015/1 and MC_UU_00006/1) and Cancer Research UK (C864/A14136). FI, NGF, and NJW were funded by the United Kingdom Medical Research Council Epidemiology Unit core grant (MC_UU_00006/1 and MC_UU_00006/3); NGF and NJW acknowledge National Institute for Health Research (NIHR) Biomedical Research Centre Cambridge (IS-BRC1215-20014). NGF is an NIHR Senior Investigator. The authors express their gratitude to the EPIC-Norfolk participants.

6. EPIC-Potsdam: European Prospective Investigation into Cancer and Nutrition (EPIC) study (Potsdam)

General information: The EPIC-Potsdam study is part of the multi-centre prospective cohort study EPIC.[9] In Potsdam, Germany, 27,548 subjects (16,644 women aged mainly 35-65 years and 10,904 men aged mainly 40-65 years) from the general population were recruited between 1994 and 1998. A case-cohort study within the prospective EPIC-Potsdam study was designed. We randomly selected 2,500 individuals from all participants of the EPIC-Potsdam study population who provided blood samples (n=26,444) for a subcohort. Cases comprised all incident type 2 diabetes cases, which were diagnosed until 2009 (n=820).

From the initial case-cohort, 371 participants came for the follow-up visit (EPIC-DZD study) into the study centre and of these, 259 participants had available creatinine measurements from baseline and follow-up visits and fatty acid biomarker measurements from baseline, who were included in the analysis for the secondary outcomes. For primary outcomes of incident chronic kidney disease, after excluding 6 adults with prevalent CKD at baseline, a total of 253 adults were eligible for the current analysis.

Measurement of fatty acid biomarker concentrations: Thirty milliliters of blood were taken from each participant during baseline examination and were centrifuged at 1,000 g for 10 min at 4°C. Plasma, serum, red blood cells, and buffy coat were removed and stored at -80°C. The erythrocyte membrane fatty acids were analysed at the Laboratory of the Dutch National Institute for Public Health and Environment between February and June 2008. Briefly, fatty acid methyl esters (FAME) were separated on a GC-3900 gas chromatograph (Varian Inc., Middelburg, Netherlands) equipped with a 100 m x 0.25 mm ID WCOT-fused silica capillary column and flame ionisation detector with separation of FAME peaks based on mixed FAME standards (Sigma Aldrich, St Louis, USA). The Galaxie software version 1.9.3.2 (Varian Inc.) was used for quantification and identification of peaks. The FAs were expressed as the percentage of total FAs present in the chromatogram. Intraassay CVs (%): ALA: 10.2, EPA: 3.1, DPA: 2.2, DHA: 2.4

Physical activity assessment: Physical activity was assessed via self-reports made in a computer-guided interview. We considered sport activities and biking as leisure time physical activities, both calculated as the average time spent per week during the twelve months before baseline recruitment.

Statistical methods for incident CKD analysis: Poisson regression

Missing data in covariates: There was only one missing data in systolic blood pressure, which were excluded from the analysis.

Funding support and acknowledgements: The EPIC-Potsdam study was supported by the German Federal Ministry of Science (01 EA 9401) and the European Union (SOC 95201408 05F02) for the recruitment phase. The follow-up of the EPIC-Potsdam Study was supported by the German Cancer Aid (70-2488-Ha I) and the European Community (SOC

98200769 05F02). This work was furthermore supported by a grant from the German Ministry of Education and Research (BMBF) and the State of Brandenburg (DZD grant 82DZD00302). The present analysis was also supported by a grant from the German Research Foundation (DFG, SCHU 1516/5-1). The authors thank the Human Study Centre (HSC) of the German Institute of Human Nutrition Potsdam-Rehbrücke, namely the trustee and the data hub for the processing, and the participants for the provision of the data, the biobank for the processing of the biological samples and the head of the HSC, Manuela Bergmann, for the contribution to the study design and leading the underlying processes of data generation. The authors thank Hans Cremers for the excellent performance of the FA analysis.

7. FDPS: Finnish Diabetes Prevention Study

General information: The Finnish DPS is a randomised study aimed at preventing type 2 diabetes through intensive lifestyle intervention, carried out at five study clinics in Finland.[10,11] The randomised trial started in November 1993, the recruitment period lasted until June 1998 and the intervention period lasted until the end of 2001. The last follow-up data were collected in 2009 with a follow-up over 13 years from baseline. Participants were recruited at five field centers (Kuopio, Helsinki, Tampere, Turku and Oulu). The cohort consisted of 522 non-institutionalised men and women. Originally, subjects at baseline were all with impaired glucose tolerance defined by repeated oral glucose tolerance tests (OGTT) (mean value of 2-hour plasma glucose concentrations 7.8-11.0 mmol/L in two OGTTs). For the fatty acid measurements, only subjects who were non-diabetic by the revised WHO 1999 criteria at baseline and had stored serum samples available from the active study period (n=407).

There were 388 adults with available data on fatty acid biomarkers and eGFR at baseline and follow-up visits, who were included in the analysis for the secondary outcomes. For primary outcomes of incident chronic kidney disease, after excluding 33 adults with prevalent CKD at baseline, a total of 355 adults were eligible for the current analysis.

Measurement of fatty acid biomarker concentrations: The total serum fatty acid composition was measured by TETHYS Bioscience Inc. (Emeryville, CA) in 2010, using stored (-80°C) serum samples taken during the active intervention period.[12] The lipids from serum were extracted in the presence of authentic internal standards by using chloroform:methanol (2:1 v/v).[6] The total lipid extract was trans esterified in 1% sulfuric acid in methanol in a sealed vial under a nitrogen atmosphere at 100°C for 45 min. The resulting extract was neutralised with 6% potassium carbonate and the fatty acid methyl esters were extracted with hexane. The isolated fatty acid methyl ester extract was then prepared for gas chromatography by sealing the hexane extracts under nitrogen. The fatty acid methyl esters were separated and quantified by capillary gas chromatography (Agilent Technologies model 6890) equipped with a 30 m DB 88MS capillary column (Agilent Technologies) and a flame ionisation detector. Proportions of fatty acids are expressed as molar percentages (mol/mol of all fatty acids). The intra- and inter-assay CV% for individual fatty acids were ≤10% and ≤12%, respectively.

Physical activity assessment: Physical activity was assessed via self-reported questionnaires.

Statistical methods for incident CKD analysis: Cox regression

Missing data in covariates: Participants with missing data in covariates were excluded from the analysis (<5%).

Funding support and acknowledgements: The FDPS study has been financially supported by the Academy of Finland (128315, 129330), Ministry of Education, Novo Nordisk Foundation, Yrjö Jahnsson Foundation, Juho Vainio Foundation, Finnish Diabetes Research Foundation, Finnish Foundation for Cardiovascular Research, Unilever, and Competitive Research Funding from Tampere, Kuopio and Oulu University Hospitals. The study sponsors had no role in the design and conduct of the study; the collection, analysis and interpretation of the data; or the preparation, review or approval of the manuscript. The authors express their gratitude to the FDPS participants.

8. FHS: Framingham Heart Study

General information: Our analysis focused on the Framingham Heart Study (FHS) Offspring sample, a population based longitudinal study of families living in Framingham, Massachusetts.[13] The offspring study was initiated in 1971 and consisted of a sample of 5,124 individuals, offspring of the original cohort and their spouses.

The offspring started with 5,124 individuals in 1971. Exam 8 (in 2008) consisted of 3,021 individuals, having lost 2,003 individuals due to death and loss to follow-up. Fatty acid data was available for 2,692 adults of the 3,021. Of these 2,692 individuals, there were 2,041 adults with available data on eGFR at baseline (Exam 8) and follow-up (Exam 9) visits, who were included in the analysis for the secondary outcomes. For primary outcomes of incident chronic kidney disease, after excluding 146 adults with prevalent CKD at baseline, a total of 1,895 adults were eligible for the current analysis.

Measurement of fatty acid biomarker concentrations: The fatty acid composition of red blood cell (RBC) samples was analysed by gas chromatography equipped with a SP 2560 capillary column after direct transesterification for 10 minutes in boron trifluoride/ methanol and hexane at 100°C as previously described. This technique generates fatty acids primarily from RBC glycerophospholipids. RBCs were isolated from blood drawn after a 10-12 h fast and frozen at -80 °C immediately after collection. All fatty acids present at >1% abundance had CVs of ≤7%.

Physical activity assessment: Self-reported values on typical activities and rest hours per day were gathered as part of the Physical Activity Questionnaire. Metabolic equivalents per week were computed using standard scoring approaches.

Statistical methods for incident CKD analysis: Poisson regression

Missing data in covariates: Participants with missing data in covariates were excluded from the analysis (<5%).

Funding support and acknowledgements: The FHS study is conducted and supported by the National Heart, Lung and Blood Institute (NHLBI) and in collaboration with Boston University (Contract No. N01-HC-25195). The authors express their gratitude to the FHS participants.

9. Hisayama: Hisayama Study

General information: The Hisayama Study is an ongoing, population-based prospective cohort study of cardiovascular disease and its risk factors in the town of Hisayama, a suburb in the metropolitan in Japan.[14] A total of 3,293 residents who were aged 40 years or older, and had no missing values for serum fatty acid levels were enrolled in the present study. There were 2,934 adults with available data on fatty acid biomarkers and eGFR at baseline and follow-up visits, who were included in the analysis for the secondary outcomes. For primary outcomes of incident chronic kidney disease, after excluding 221 adults with prevalent CKD at baseline, a total of 2,713 adults were eligible for the current analysis.

Measurement of fatty acid biomarker concentrations: Serum fatty acids levels were assayed by gas chromatography (SRL, Tokyo, Japan). Briefly, total lipids in plasma were extracted according to the Folch's procedure[6], followed by hydrolysis to free fatty acids. Free fatty acids were esterified with potassium methoxide/methanol and boron trifluorideemethanol. The methylated fatty acids were analysed using GC-17A gas chromatograph (Shimadzu Corporation, Kyoto, Japan) with omegawax-250 capillary column (SUPELCO, Sigmae Aldrich Japan, Tokyo, Japan). Reproducibility (i.e. the coefficient of variation) of the determination of serum EPA, DHA, and ALA levels by this method was reported to be 4.4%, 2.3%, and 3.8%, respectively.[15]

Physical activity assessment: Physical activity was assessed as frequency of leisure-time exercise/week.

Statistical methods for incident CKD analysis: Cox regression

Missing data in covariates: There were missing data on alcohol consumption (2.9%) and urine albumin-creatinine ratio (0.8%). For missing covariate data, dummy variable indicating missing value was used.

Funding support and acknowledgements: This study was supported in part by Grants-in-Aid for Scientific Research A (JP16H02692), B (JP17H04126, JP18H02737,and JP19H03863), C (JP18K07565, JP18K09412, JP19K07890, JP20K10503,and JP20K11020), Early-Career Scientists (JP18K17925) , and Research Activity Start-up (JP19K23971) from the Ministry of Education, Culture, Sports, Science and Technology of Japan; by Health and Labour Sciences Research Grants of the Ministry of Health, Labour and Welfare of Japan (20FA1002); and by the Japan Agency for Medical Research and Development (JP20dk0207025, JP20km0405202, JP20fk0108075). The authors express their gratitude to the Hisayama residents and the staff of the Division of Health and Welfare of Hisayama for their cooperation in this study.

10. InCHIANTI: Invecchiare in Chianti

General information: The InCHIANTI study is a population-based epidemiological study aimed at evaluating the factors that influence mobility in the older population living in the Chianti region in Tuscany, Italy.[16] 1,616 residents were selected from the population registry of Greve in Chianti (a rural area: 11,709 residents with 19.3% of the population greater than 65 years of age), and Bagno a Ripoli (Antella village near Florence; 4,704 inhabitants, with 20.3% greater than 65 years of age). The participation rate was 90% (n=1,453), and the subjects ranged between 21-102 years of age. Following the baseline visit between 1998-2000, there were four follow up visits at 2001-2003, 2004-2006, 2007-2009, and 2013-2014.

Among 1,453 adults, there was 1,177 adults with available data on fatty acid biomarkers and eGFR at baseline. After excluding 80 adults (6.8%) with missing data in the covariates, there were 962 participants with data on follow-up eGFR, who were included in the analysis for the secondary outcomes. For primary outcomes of incident chronic kidney disease, after excluding 132 adults with prevalent CKD at baseline, a total of 830 adults were eligible for the current analysis.

Measurement of fatty acid biomarker concentrations: Plasma fatty acids measurement method has been described previously.[17] Briefly, blood samples were collected in the morning after a 12-hr overnight fast. Aliquots of plasma were immediately obtained and stored at -80°C. Fatty acid methyl esters (FAME) were prepared through transesterification using Lepage and Roy's method with modification.[6,18] Separation of FAME was carried out on an HP-6890 gas chromatograph (Hewlett-Packard, Palo Alto, CA) with a 30-m fused silica column (HP-225; Hewlett-Packard). FAMEs were identified by comparison with pure standards (NU Chek Prep, Inc., Elysian, MA). For quantitative analysis of fatty acids as methyl esters, calibration curves for FAME (ranging from C14:0 to C24:1) were prepared by adding six increasing amounts of individual FAME standards to the same amount of internal standard (C17:0; 50xg). The correlation coefficients for the calibration curves of fatty acids were in all cases higher than 0.998 in the range of concentrations studied. Fatty acid concentration was expressed as a percentage of total fatty acids. The coefficient of variation for all fatty acids was on average 1.6% for intraassay and 3.3% for inter-assay.

Physical activity assessment: Physical activity in the previous year was assessed as i) sedentary (hardly any physical activity, ii) mostly sitting/some walking, iii) Light exercise 2-4 hr/week, iv) moderate 1-2 hr/week, v) moderate exercise >3 hr/week, vi) intensive exercise many times/week and vii) walk over 5 km/day for 5 days for at least 5 years.

Statistical methods for incident CKD analysis: Cox regression

Missing data in covariates: As described in the "General information" section above, participants with missing data in covariates were already excluded from the analysis.

Funding support and acknowledgements: The InCHIANTI study baseline (1998-2000) was supported as a "targeted project" (ICS110.1/RF97.71) by the Italian Ministry of Health and in part by the U.S. National Institute on Aging (Contracts: 263 MD 9164 and 263 MD 821336); the InCHIANTI Follow-up 1 (2001-2003) was funded by the U.S. National Institute on Aging (Contracts: N.1-AG-1-1 and N.1-AG-1-2111); the InCHIANTI Follow-ups 2 and 3 studies (2004-2010) were financed by the U.S. National Institute on Aging (Contract: N01-AG-5-0002);supported in part by the Intramural Research Program of the National Institute on Aging, National Institutes of Health, Baltimore, Maryland. The authors express their gratitude to the InCHIANTI participants.

11. KIHD: Kuopio Ischaemic Heart Disease Risk Factor Study

General information: The KIHD study was designed to investigate risk factors for cardiovascular diseases, atherosclerosis, and related outcomes in a population-based, randomly selected sample of men from eastern Finland.[19] The baseline examinations were carried out in 1984-1989. A total of 2,682 men who were 42, 48, 54 or 60 years old at baseline (82.9% of those eligible) participated. The 20-year examinations were conducted in 2005-2008 for 1,241 men (79.7% of the eligible).

Among 2,682 men, there was 1,091 men with available data on fatty acid biomarkers and eGFR at baseline and follow-up visits, who were included in the analysis for the secondary outcomes. For primary outcomes of incident chronic kidney disease, after excluding 9 men with prevalent CKD at baseline, a total of 1,082 men were eligible for the analysis.

Measurement of fatty acid biomarker concentrations: Venous blood samples were collected between 8AM and 10AM after an overnight fast. Serum total fatty acids were determined from frozen samples with a NB-351 capillary column (HNU-Nordion, Helsinki, Finland) by a Hewlett-Packard 5890 Series II gas chromatograph (Hewlett-Packard Company, Avondale, PA, USA, since 1999 Agilent Technologies Inc., USA) with a flame ionisation detector. Serum was extracted with chloroform-methanol and fatty acids were methylated with methanol and sulphuric acid prior to gas chromatography. Each analyte had an individual reference standard and the analytes were quantified with an internal standard method using eicosane. The coefficient of variation (CV%) for repeated measurements of fatty acids was 12.8% for ALA, 9.4% for EPA, 12.7% for DPA, and 11.9% for DHA.

Physical activity assessment: Physical activity was evaluated based on the 12-month leisure-time physical activity questionnaire and expressed as kcal/day.

Statistical methods for incident CKD analysis: Poisson regression

Missing data in covariates: The cohort mean was used to replace missing values in covariates (n=4).

Funding support and acknowledgements: The KIHD study was supported mainly by the funding from the Academy of Finland to Jukka T. Salonen. The authors express their gratitude to the KIHD participants.

12. MAS: Memory and Ageing Study

General information: The Sydney Memory and Ageing Study (MAS) is a longitudinal study which began in 2005 with the aims of investigating rates and predictors of healthy cognitive ageing, mild cognitive impairment (MCI) and dementia in older Australians.[20] Over the last 14 years, MAS has collected biomarker, genetic/epigenomic, neuroimaging, cognitive, proteomics/lipidomics, health, and lifestyle data to determine what factors are associated with cognitively normal ageing and progression to MCI or dementia. At baseline, there were 1,037 older adults without dementia, aged 70-90 recruited from Eastern Sydney, Australia.

Among 1,037 adults, there were 395 adults with available data on fatty acid biomarkers and eGFR at baseline and follow-up visits without any missing data on covariates, who were included in the analysis for both primary and secondary outcomes as there were no participants with prevalent CKD at baseline.

Measurement of fatty acid biomarker concentrations: Twenty-nine free, bound and total fatty acids in plasma was quantified using negative ion chemical ionisation gas chromatography - mass spectrometry (GCMS) approach as originally reported by Quehenberger O et al who developed a rapid protocol for the extraction of free and bound fatty acids from complex biological samples including plasma.[21] Fatty acids were quantified using the stable isotope dilution method, which was based on the principle that each target fatty acid was compared to a deuterated analog with similar chemical and structural properties which was added to all samples and standards at identical amounts. This allowed accurate quantification and also compensated for any losses during sample preparation and analysis. Enrichment and

extraction of free fatty acids were achieved through addition of acidified methanol and isooctane and then deuterated fatty acids which acted as internal standards for quantification and to compensate for any losses during sample preparation. Samples were shaken and the two resulting solvent phases separated. The upper isooctane layer containing the free fatty acid fraction was removed, and extraction steps repeated and resulting extractions pooled. Samples were then evaporated to dryness. Hydrolysis of the remaining methanol fraction containing the esterified or bound fatty acid fraction was completed using KOH and incubation for 1 hr at 37°C, followed by extraction of the released fatty acid using isooctane procedure described above. All samples were then derivatised to pentafluorobenzyl esters and reconstituted in isooctane before analysis. Standard curves of primary (unlabelled) fatty acid standards with added internal standards were run alongside samples. The ratios of unlabelled to labelled standards from the standard curves were used to quantify levels of fatty acids in plasma samples. Total fatty acid levels were calculated as sum of free and bound fatty acids, and then % of individual fatty acids of interest of total values were calculated for ALA, DHA, DPA and EPA. Intra-assay CVs were generally <15% and inter-assay CVs <5% for all fatty acids tested.

Physical activity assessment: The physical activity index represented the number of physical activities participated in, regardless of the amount or intensity of participation. The maximum score was 10 points. Data was categorised into three groups.

Statistical methods for incident CKD analysis: Poisson regression

Missing data in covariates: There were 20 (5.1%) participants with missing data on the use of any anti-hypertensive drugs and use of lipid-lowering drugs. There was no missing data for other covariates. Imputation was performed for missing data.

Funding support and acknowledgements: The MAS study has been funded by three National Health & Medical Research Council (NHMRC) Program Grants (ID No. ID350833, ID568969, and APP1093083). The fatty acid analysis was funded by the NHMRC Investigator Grant (APP1196150). The authors thank the participants and their informants for their time and generosity in contributing to this research. The authors also acknowledge the MAS research team: https://cheba.unsw.edu.au/research-projects/sydney-memory-and-ageing-study. Blood samples were collected by South Eastern Area Laboratory Service (SEALS). The authors express their gratitude to the MAS participants.

13. MESA: Multi-Ethnic Study of Atherosclerosis

General information: The Multi-Ethnic Study of Atherosclerosis (MESA) is a study of the characteristics of subclinical cardiovascular disease (disease detected non-invasively before it has produced clinical signs and symptoms) and the risk factors that predict progression to clinically overt cardiovascular disease or progression of the subclinical disease.[22] MESA researchers study a diverse, population-based sample of 6,814 asymptomatic individuals of European, African, Hispanic, and Chinese American ancestry ascertained across six field centres across the United States. Baseline data for the current analyses were taken from the first clinic exam conducted in 2000-2002. In addition to yearly phone calls, follow-up clinic exams are conducted approximately every two years, and at the time the current analyses were conducted incident diabetes was available until the fifth clinic exam conducted in 2010-2012.

Among 6,814 adults, there was 1,841 adults with available data on fatty acid biomarkers and eGFR at baseline and follow-up visits, who were included in the analysis for the secondary outcomes. For primary outcomes of incident chronic kidney disease, after excluding 159 adults with prevalent CKD at baseline, a total of 1682 adults were eligible for the current analysis.

Measurement of fatty acid biomarker concentrations: Fatty Acid analysis was performed on plasma, that had been stored at -70°C, using the method previously described.[23] For the extraction of phospholipid fatty acids, plasma was diluted in saline and lipids were extracted from with a mixture of chloroform:methanol, and cholesterol, triglycerides and phospholipid subclasses were separated on a silica thin-layer chromatography plate in a solvent mixture of petroleum ether, diethyl ether, and glacial acetic acid. The band of phospholipids is harvested for the formation of methyl esters. Fatty acid methyl esters are prepared with 14% boron trifluoride in methanol, incubated at 80°C for 90 minutes, and extracted with petroleum ether. The final product is dissolved in heptane and injected onto a capillary

Varian CP7420 100-m column with a Hewlett Packard 5890 gas chromatograph equipped with a HP6890A autosampler. The gas chromatograph is configured for a single capillary column with a flame ionisation detector and interfaced with HP ChemStation software. Separation of individual fatty acids was obtained over an 80-minute run. Fatty acids are expressed as a percent of total fatty acids. The following CVs were obtained on 20 blind duplicates: ALA= 2.4%; EPA= 3.3%; DPA = 2.9%, DHA= 2.7%.

Physical activity assessment: The MESA Typical Week Physical Activity Survey (TWPAS) was adapted from the Cross-Cultural Activity Participation Study and designed to measure weekly duration and frequency of various leisure physical activities. The survey has 28 items in categories of household chores, lawn/yard/garden/farm, care of children/adults, transportation, leisure walking, dancing and sport activities, conditioning activities, leisure activities, and occupational and volunteer activities. Questions differentiated between light-, moderate-, and heavy-intensity activities. Minutes of activity were summed for each activity and multiplied by metabolic equivalent (MET) level.

Statistical methods for incident CKD analysis: Cox regression

Missing data in covariates: Participants with missing data in covariates were excluded from the analysis.

Funding support and acknowledgements: MESA and the MESA SHARe project are conducted and supported by the National Heart, Lung, and Blood Institute (NHLBI) in collaboration with MESA investigators. Support for MESA is provided by contracts 75N92020D00001, HHSN268201500003I, N01-HC-95159, 75N92020D00005, N01-HC-95160, 75N92020D00002, N01-HC-95161, 75N92020D00003, N01-HC-95162, 75N92020D00006, N01-HC-95163, 75N92020D00004, N01-HC-95164, 75N92020D00007, N01-HC-95165, N01-HC-95166, N01-HC-95167, N01-HC-95168, N01-HC-95169, UL1-TR-000040, UL1-TR-001079, UL1-TR-001420. Also supported in part by the National Center for Advancing Translational Sciences, CTSI grant UL1TR001881, and the National Institute of Diabetes and Digestive and Kidney Disease Diabetes Research Center (DRC) grant DK063491 to the Southern California Diabetes Endocrinology Research Center. The authors thank the other investigators, the staff, and the participants of the MESA study for their valuable contributions. A full list of participating MESA investigators and institutions can be found at http://www.mesa-nhlbi.org .

14. METSIM: METabolic Syndrome in Men

General information: The METSIM study includes 10,197 men, aged from 45 to 73 years at entry, randomly selected from the population register of the Kuopio town, Eastern Finland, and examined in 2005-2010 (baseline).[24-26] The aim of the study is to investigate nongenetic and genetic factors associated with type 2 diabetes and cardiovascular disease, and with cardiovascular risk factors in both cross-sectional and longitudinal settings.

Fatty acid composition of erythrocyte membranes and plasma lipids was available in 1,364 participants at baseline. There were 1,158 adults with available data on fatty acid biomarkers and eGFR at baseline and follow-up visits, who were included in the analysis for the secondary outcomes. For primary outcomes of incident chronic kidney disease, after excluding 6 adults with prevalent CKD at baseline, a total of 1,152 adults were eligible for the current analysis.

Measurement of fatty acid biomarker concentrations:
Phospholipids, cholesterol esters and triglycerides: Lipids were extracted from plasma sample with chloroform-methanol (2:1) and lipid fractions were separated with an aminopropyl column. FAs in lipid fractions were trans-methylated with 14% borontrifluoride in methanol. Finally, fatty acid methyl esters were analysed by 7890A gas-chromatograph (Agilent Technologies, Inc., Wilmington, DE, USA) equipped with a 25-m free FA phase column (Agilent Technologies). Cholesteryl nonadecanoate (Nu Chek Prep, Inc., Elysian, MA, USA), trinonadecanoin and phosphatidylcholine dinonadecanoyl (Larodan Fine Chemicals, Malmo, Sweden) served as internal standards. The intra-assay CVs for the n-3 fatty acids were between 0.3-3.6% and the inter-assay CVs between 2.0-14.0%

Erythrocyte membranes: Erythrocytes were separated from EDTA-blood and then hemolysed in the tris-HCl buffer (pH 7.6, 10 mmol/L). Fatty acid methyl esters were prepared by direct trans-esterification using acetyl chloride and analysed by 7890A gas-chromatograph (Agilent Technologies, Inc., Wilmington, DE, USA) equipped with a 25-m free FA phase column (Agilent Technologies). Pure standards (NU Chek Prep Inc) were used to identify FA methyl esters and to

prepare calibration curves. Heptadecanoic acid methyl ester (17:0) served as an internal standard. The intra-assay CVs for the n-3 fatty acids were between 1.1-3.3% and the inter-assay CVs between 4.2-9.9%

Physical activity assessment: Leisure time physical activity was assessed by self-report at baseline using 4 categories:
1st=a little or none
2nd= Physical exercise in context of other hobbies or physical exercise occasionally
3rd= Physical exercise ≤2 times a week at least 30 min at a time
4th= Physical exercise regularly ≥3 times a week at least 30 min at a time

Statistical methods for incident CKD analysis: Poisson regression

Missing data in covariates: There was only one missing value (0.1%) in prevalent diabetes, which was excluded from the analysis.

Funding support and acknowledgements: The METSIM Study was funded by the grants from The European Union, the Academy of Finland, and the Juselius Foundation. The authors express their gratitude to the METSIM participants.

15. NHAPC: Nutrition and Health of Aging Populations in China

General information: The NHAPC study is a prospective population-based cohort study of people 50-70 years old in Beijing and Shanghai at baseline initiated to evaluate risk factors for metabolic disease.[27-29] Participants were recruited at two large cities representing the north and the south of China, respectively, two urban districts and one rural district in each city (Dongcheng, Beijing; Xuanwu, Beijing; Changping, Beijing; Luwan, Shanghai; Zhabei, Shanghai; Fengxian, Shanghai) from random samples of Medicare eligibility lists. The cohort consists of 3,289 non-institutionalised men and women, recruited in 2005, 2,529 participants were successfully followed for 6 years.

Among 3,289 adults, there were 2,240 adults with available data on fatty acid biomarkers and eGFR at baseline and follow-up visits. After excluding 100 adults with outliers in any of the fatty acids (defined as < 25th percentile - (1.5 x interquartile range) or >75th percentile + (1.5 x interquartile range)), 2,140 adults were included in the analysis for the secondary outcomes. For primary outcomes of incident chronic kidney disease, 94 adults with prevalent CKD at baseline were excluded, a total of 2,046 adults were eligible for the current analysis.

Measurement of fatty acid biomarker concentrations: Erythrocyte membrane fatty acids were measured at the Mass Spectrometry Subplatform in the Public Technical Service Center of Shanghai Institute of Nutrition and Health, Chinese Academy of Sciences (Shanghai, China) using stored blood samples in 2005. Total lipids were extracted from plasma using the methods of Folch et al.[6] Erythrocyte samples were first extracted by hexane and isopropanol and then trans-methylated with methanol and sulfuric acid. Fatty acid methylesters were subsequently analysed by gas chromatography-cationic flame system (Agilent 6890GC, Bellefonte, Pennsylvania); The chromatographic column is a fused quartz capillary column SP2560(Supelco, Belefonte, Pennsylvania). According to the internal standard and known mass of fatty acid methyl ester mixture (GLC569B; NuCheck Prep, Elysium, Minnesota) as the standard to quantification. All fatty acids were processed at the Laboratory in Shanghai Institute of Nutrition and Health (Shanghai, China). For this analysis, the relative content of each fatty acid is expressed as the ratio of its area under the peak to the area under the peak of the total fatty acid. The CVs for de novo fatty acid assays ranged from 0.34% for 18:1n29 to 9.8% for 16:1n27. Intraassay coefficients of variation (CV; percentages) were 2.7% for ALA, 25.8% for EPA, 3.6% for DPA, 3.4% for DHA, and 0.7% for linoleic acid. The corresponding inter-assay CV were 7.8, 9.3, 14.1, 10.0, and 7.7%, respectively, for these fatty acids.

Physical activity assessment: Physical activity levels were assessed using a shortened version of the International Physical Activity Questionnaire, which collected the frequency and average duration of physical activity such as heavy, medium, light and sedentary activities over the past 7 days.

Statistical methods for incident CKD analysis: Poisson regression

Missing data in covariates: There was no missing data for covariates.

Funding support and acknowledgements: The NHAPC study was funded by grants KSCX2-2-25, 04DZ14007, 200306 and 973 Program 2006CB503900 from Chinese Academy of Sciences, from the Science and Technology Commission of Shanghai Municipality, from the Shanghai-Unilever Research Development Fund and the Ministry and Technology of China, respectively. The content is solely the responsibility of the authors and does not necessarily represent the official views of the National Institutes of Health. The authors express their gratitude to the NHAPC participants.

16. PIVUS: Prospective Investigation of the Vasculature in Uppsala Seniors

General information: The PIVUS study started in 2001 with the primary aim to investigate the predictive power of different measurements of endothelial function and arterial compliance in a random sample of just over 1,000 subjects aged 70 living in the community of Uppsala.[30] As secondary aims, the study also included measurements of cardiac function and structure by ultrasound and magnetic resonance imaging, evaluation of atherosclerosis by ultrasound and magnetic resonance imaging, 7-day food intake recordings, detailed electrocardiogram analysis, cardiovascular autonomic function, body composition by DXA, DNA analysis and lung function, as well as a number of biochemical markers.

There were up to 1,016 adults with available data on fatty acid biomarkers and eGFR at baseline and follow-up visits, who were included in the analysis for the secondary outcomes. For primary outcomes of incident chronic kidney disease, after excluding 123 adults with prevalent CKD at baseline, a total of 893 adults were eligible for the current analysis.

Measurement of fatty acid biomarker concentrations: Fatty acid composition in cholesterol esters and phospholipids were measured by gas chromatography. Serum (0.5 mL) was mixed with 2.5 mL methanol, 5 mL chloroform (with 0.005% added butylated hydroxytoluene, BHT) and 7.5 mL NaH_2PO_4 (0.2 mol/L) and stored in 4°C over night for lipid extraction. The chloroform phase was then removed with a syringe and evaporated to dryness on a 30°C heating block using nitrogen gas. The lipid residue was dissolved in chloroform and the lipid fractions were separated by thin-layer chromatography (TLC); the adsorbent containing POPOP as fluorescent agent. The TLC-plates were eluted at room temperature with the solvent system petroleum ether/diethyl ether/acetic acid (81:18:1 by volume). The lipid fractions were visualised in UV light and the spots containing cholesterol esters and phospholipids were scraped off into vials and methylated at 60°C overnight after addition of 2 mL H_2SO_4 (5%) in methanol. The fatty acid methyl esters were extracted into 3 mL petroleum ether (0.005% BHT) after addition of 1.5 mL distilled water. The phases were separated after thorough mixing and centrifugation at 1,500g for 10 min. The petroleum ether phase was pipetted off and the solvent was evaporated under nitrogen gas on a 30°C heating block. The fatty acid methyl esters were dissolved in 120 µL hexane and placed in vials. The fatty acid methyl esters were separated by gas-liquid chromatography on a 30-m glass capillary column coated with Thermo TR-FAME (Thermo Electron Corporation, USA) with helium gas as a carrier gas. An Agilent Technologies system consisting of model GLC 6890N, autosampler 7683 and Agilent ChemStation was used. The temperature was programmed to 150-260°C. The fatty acids were identified by comparing each peak´s retention time with fatty acid methyl ester standards Nu Check Prep (Elysian, MN, USA). Fatty acids are presented as the relative sum of the fatty acids analysed in each compartment. For concentrations below detection limit, proportions were imputed as a random value between 0 and the minimum quantified proportion in the cohort.

Physical activity assessment: Physical activity levels were assessed using self-reported activities.

Statistical methods for incident CKD analysis: Poisson regression

Missing data in covariates: Participants with missing data in covariates were excluded from the analysis (12%).

Funding support and acknowledgements: The PIVUS study was supported by Uppsala University Hospital; The Swedish Research Council for Health, Working Life and Welfare; The Swedish Research Council (K2015-54X-22081-04-3); and The Swedish Research Council for Environment, Agricultural Sciences and Spatial Planning (2016-01639). The authors express their gratitude to the PIVUS participants.

17. ULSAM: Uppsala Longitudinal Study of Adult Men

General information: The ULSAM study is an ongoing, longitudinal, epidemiologic study based on all available men, born between 1920 and 1924 and living in Uppsala County, Sweden.[31] The men were investigated at the ages of 50, 60, 70, 77, 82, 88 and 93 years. The reinvestigations in ULSAM were based on the previous investigations. Full screening and official registry data are available in our databases and more data is continuously added.

Among 2,294 adults, there were up to 1,055 adults with available data on fatty acid biomarkers and eGFR at baseline and follow-up visits, who were included in the analysis for the secondary outcomes. For primary outcomes of incident chronic kidney disease, after excluding 0 adults with prevalent CKD at baseline, a total of up to 1,055 adults were eligible for the current analysis.

Measurement of fatty acid biomarker concentrations: For analysis of the fatty acid composition of the serum cholesterol esters, serum was extracted with a hexane-isopropanol solution (1+4). Cholesterol esters were separated from the extract by thin layer chromatography before inter-esterification (acidic methanol at 85°C, 2 h), and free cholesterol liberated in the reaction was removed by an aluminium oxide column to avoid contamination of the gas liquid chromatography column. The percentage composition of methylated fatty acids 14:0 to 22:6 was determined by gas chromatography (a 25 m NB-351 silica capillary column, i.d. 0.32 mm, phase layer 0.20 mm) with use of a flame ionisation detector and with helium as carrier gas. Every 25th sample was a serum control pool. The precision of the between-series analysis (n=35) varied from 2% (large peaks) to 10% (smaller peaks) and between successive gas chromatography runs (n=17) from 0.2 to 5% (CV).

Physical activity assessment: Physical activity was assessed by self-reported questionnaire, and participants were classified into sedentary, moderate, strenuous and athletic categories according to the duration and vigorousness of their physical activity.

Statistical methods for incident CKD analysis: Poisson regression

Missing data in covariates: Participants with missing data in covariates were excluded from the analysis (14%).

Funding support and acknowledgements: The Uppsala Longitudinal Studies of Adult Men 50 and 70 were funded by the Swedish Research Council for Health, Working Life and Welfare; Uppsala City Council; and The Swedish Research Council (K2015-54X-22081-04-3); and The Swedish Research Council for Environment, Agricultural Sciences and Spatial Planning (2016-01639). The authors express their gratitude to the ULSAM participants.

18. WHIMS: Women's Health Initiative Memory Study

General information: WHIMS was a randomised trial which examined the effects of postmenopausal hormone therapy on cognitive function in women aged 65-80 years.[32,33] Recruitment began in 1995.

Among 7,479 adults, there was 1,785 adults with available data on fatty acid biomarkers and eGFR at baseline and follow-up visits, who were included in the analysis for the secondary outcomes. For primary outcomes of incident chronic kidney disease, after excluding 99 adults with prevalent CKD at baseline, a total of 1,686 adults were eligible for the current analysis.

Measurement of fatty acid biomarker concentrations: The fatty acid composition of red blood cell (RBC) samples was analysed by gas chromatography equipped with a SP 2560 capillary column after direct transesterification for 10 minutes in boron trifluoride/ methanol and hexane at 100°C as previously described.[34] This technique generates fatty acids primarily from RBC glycerophospholipids. During the aliquoting phase, the RBC samples were stored improperly at -20°C for a period of approximately 2 weeks, causing oxidative degeneration of the PUFAs before measurement. The original FA levels were estimated with multiple imputations using independent data on fatty acid degradation and length of time the samples were exposed to -20°C.[34] All fatty acids present at >1% abundance had CVs of ≤6.5%.

Physical activity assessment: Self-reported information on sedentary behaviour and usual physical activity was assessed as Mets/week.

Statistical methods for incident CKD analysis: Poisson regression

Missing data in covariates: Participants with missing data in covariates were excluded from the analysis (<5%).

Funding support and acknowledgements: The WHI program is funded by the National Heart, Lung, and Blood Institute, National Institutes of Health, U.S. Department of Health and Human Services through contracts HHSN268201600018C, HHSN268201600001C, HHSN268201600002C, HHSN268201600003C, and HHSN268201600004C. The authors thank the WHI investigators and staff for their dedication, and the study participants for making the program possible. A listing of WHI investigators can be found at http://www.whi.org/researchers/Documents%20%20Write%20a%20Paper/WHI%20Investigator%20Short%20List.pdf.

19. 3C Study: Three City Study (3C)

General information: The Three-City (3C) study is an ongoing multi-centre prospective cohort study of vascular risk factors for dementia which started in 1999-2000 and included 9,294 community dwellers in three French cities: Bordeaux (n=2,104), Dijon (n=4,931) and Montpellier (n=2,259).[35] Individuals living in one of these cities, aged 65 years and over and not institutionalised were eligible for recruitment into the 3C study. The protocol of the 3C study has been approved by the Consultative Committee for the Protection of Persons participating in Biomedical Research of the Kremlin-Bicêtre University Hospital (Paris). All participants gave their written informed consent. The baseline data collection included socio-demographic and lifestyle characteristics, symptoms and complaints, main chronic conditions, medication use, neuropsychological testing, clinical examination including blood pressure measurement, electrocardiogram and blood sampling. Total plasma fatty acids were measured at baseline from fasting blood samples among 1,416 individuals from the Bordeaux centre. Moreover, fatty acid composition of red blood cell membrane phospholipids were measured at baseline from fasting blood samples among 670 individuals from the Bordeaux and Montpellier centres.

Among 2,104 adults from 3C Bordeaux study centre, there was 1,130 adults with available data on plasma fatty acid biomarkers and eGFR at baseline and follow-up visits, who were included in the analysis for the secondary outcomes. For primary outcomes of incident chronic kidney disease, after excluding 111 adults with prevalent CKD at baseline, a total of 1,019 adults were eligible for the current analysis of plasma fatty acid biomarkers.

Among 4,363 adults from 3C Bordeaux and Montpellier study centres, 710 adults were involved in an ancillary study on erythrocyte fatty acids and among them, there was 422 adults with available data on fatty acid biomarkers and eGFR at baseline and follow-up visits, who were included in the analysis for the secondary outcomes. For primary outcomes of incident chronic kidney disease, after excluding 49 adults with prevalent CKD at baseline, a total of 373 adults were eligible for the current analysis of erythrocyte fatty acids.

Measurement of fatty acid biomarker concentrations: In the 3C study, fasting blood samples were collected at the baseline visit into heparinised evacuated tubes and centrifuged at 1000 x g for 10 min. Total lipids were extracted from plasma with 5 mL of hexane/isopropanol (3:2, by vol). The plasma fatty acid composition was determined from 2 mL of the lipid extract after transformation into isopropyl esters. Separation of isopropyl esters was made on a gas chromatograph (Trace, Thermoelectron, France) using a 25-m Carbowax capillary column (internal diameter: 0.32 mm). Column conditions were 180 °C for 5 min, increasing by 7.5°C/min to 220°C for 30 min. The injector was set at 60 °C and the flame ionisation detector at 250°C. Helium was used as the carrier gas (flow rate: 2 mL/min). The peaks were identified by comparison with reference fatty acid esters (Sigma, St Louis, MO), and peak areas were measured with an automatic integrator (DP700; Fisons Instruments, France). The results for each fatty acid were expressed as a percentage of total fatty acids.

Erythrocyte membrane phospholipid fatty acids were measured at the French Institute for Fats and Oils (ITERG). Total lipids from red blood cell membranes were extracted as described previously.[36] A one dimensional thin-layer chromatography was used to separate total phospholipids of red blood cells from neutral lipids. Total fatty acids of the red blood cell phospholipid fraction were methylated to obtain fatty acid methyl esters. Individual fatty acid methyl esters were separated using a gas chromatograph (Focus GC, Thermo Scientific, France) equipped with a flam ionisation detector and a split injector. A fused silica capillary column (BPX 70, 60m x 0.25mm internal diameter, 0.25mm film; Phenomenex, Germany) was used with H_2 as the carrier gas (inlet pressure: 1 bar). The column temperature was programmed to increase from 150 to 200°C at 1.5°C/min for 25 min, and then from 200 to 225°C at 20°C/min and was held at 225°C until the completion of the analysis (20 min). The injection port and detector were maintained at 250 and 280°C, respectively. Data were integrated using the ChromQuest Software (Thermo Scientific). Individual fatty acid methyl esters were identified by comparing their retention times with those of authentic standards eluted in the same conditions (Sigma Chemical Co., Saint Quentin Fallavier, France). The results are expressed as a weight percentage of total fatty acids.

Physical activity assessment: Regular exercise was defined as engaging in moderate to vigorous physical activity (defined as recreational walking ≥1 hours per day or practicing sport ≥1 times per week).

Statistical methods for incident CKD analysis: Cox regression

Missing data in covariates: Only body mass index and physical activity had missing data (2.3% and 16% respectively). Participants with missing data in body mass index (continuous) were excluded from the analysis. Missing values for physical activity were imputed to the highest frequency category.

Funding support and acknowledgements: The Three-City Study is conducted under a partnership agreement between Sanofi-Aventis, the Institut National de la Santé et de la Recherche Médicale (INSERM) and the Institut de Santé Publique et Développement of the Victor Segalen Bordeaux 2 University. The Fondation pour la Recherche Médicale (FRM) funded the preparation and initiation of the study. The 3C Study is also supported by the Caisse Nationale Maladie des Travailleurs Salariés (CNAMTS), Direction Générale de la Santé (DGS), Mutuelle Générale de l'Education Nationale (MGEN), Institut de la Longévité, Regional Councils of Aquitaine and Bourgogne, Fondation de France, and Ministry of Research - INSERM Programme "Cohortes et collections de données biologiques.", Agence Nationale de la Recherche ANR PNRA 2006 and LongVie 2007, the "Fondation Plan Alzheimer" (FCS 2009-2012), the Caisse Nationale de Solidarité pour l'Autonomie (CNSA) and Roche Pharma. The authors express their gratitude to the 3C participants.

20. Covariates

All the covariates were prespecified and harmonised between studies. These covariates include age (years); sex (male/female); race (Caucasian/non-Caucasian, or study-specific); clinical centre / field site, if applicable (study-specific categories); education (<high school, high school graduate or >high school; if unavailable, cohort-specific categories); occupation (using cohort specific categories); body mass index (kg/m^2); smoking (current, former or never; if history of smoking not assessed, then current or non-current); alcohol intake (none, <1 drink/day, 1-2 drink/day or >2 drink/day; if unavailable, cohort-specific categories); physical activity (quartiles of metabolic equivalents per week; if unavailable, cohort-specific categories or definition); prevalent coronary heart disease (defined by ICD-9 410-414, or ICD-10 I20-I25, or cohort-specific definitions); use of lipid-lowering drugs; prevalent diabetes mellitus (defined as treatment with oral hypoglycaemic agents or insulin, fasting glucose ≥ 126mg/dL, or cohort-specific definitions); urine albumin-creatinine ratio (mg/g); systolic blood pressure (mm Hg); use of angiotensin-converting enzyme inhibitors or angiotensin receptor blockers (yes/no); and use of other anti-hypertensive drugs (yes/no) (if data on specific types of anti-hypertensive drugs were not available, then use of any anti-hypertensive drugs).

References

1. Johanna M Geleijnse, Erik J Giltay, Evert G Schouten, Janette de Goede, Linda M Oude Griep, Anna M Teitsma-Jansen, Martijn B Katan, Daan Kromhout, Alpha Omega Trial Group. Effect of low doses of n-3 fatty acids on

cardiovascular diseases in 4,837 post-myocardial infarction patients: design and baseline characteristics of the Alpha Omega Trial. *Am Heart J* 2010;159:539-46 (PubMed PMID: 20362710).

2. Wright JD, Folsom AR, Coresh J, Sharrett AR, Couper D, Wagenknecht LE, Mosley TH Jr, Ballantyne CM, Boerwinkle EA, Rosamond WD, Heiss G. The ARIC (Atherosclerosis Risk In Communities) Study: JACC Focus Seminar 3/8. *J Am Coll Cardiol* 2021;77:2939-59 (PubMed PMID: 34112321).
3. Lee Y, Lin RS, Sung FC, Yang C, Chien K, Chen W, Su T, Hsu H, Huang Y. Chin-Shan Community Cardiovascular Cohort in Taiwan-baseline data and five-year follow-up morbidity and mortality. *J Clin Epidemiol* 2000;53:838-46 (PubMed PMID: 10942867).
4. Lin HJ, Chen ST, Wu HY, Hsu HC, Chen MF, Lee YT, Wu KY, Chien KL. Urinary biomarkers of oxidative and nitrosative stress and the risk for incident stroke: a nested case-control study from a community-based cohort. *Int J Cardiol* 2015;183:214-20 (PubMed PMID: 25675904).
5. Fried LP, Borhani NO, Enright P, Furberg CD, Gardin JM, Kronmal RA, Kuller LH, Manolio TA, Mittelmark MB, Newman A, et al. The Cardiovascular Health Study: design and rationale. *Ann Epidemiol* 1991;1:263-76 (PubMed PMID: 1669507).
6. Folch J, Lees M, Sloane Stanley GH. A simple method for the isolation and purification of total lipides from animal tissues. *J Biol Chem* 1957;226:497-509 (PubMed PMID: 13428781).
7. Day N, Oakes S, Luben R, Khaw KT, Bingham S, Welch A, Wareham N. EPIC-Norfolk: study design and characteristics of the cohort. European Prospective Investigation of Cancer. *Br J Cancer* 1999;80(Suppl 1):95-103 (PMID: 10466767).
8. Khaw KT, Friesen MD, Riboli E, Luben R, Wareham N. Plasma phospholipid fatty acid concentration and incident coronary heart disease in men and women: the EPIC-Norfolk prospective study. PLoS Med 2012;9:e1001255 (PubMed PMID: 22802735).
9. Boeing H, Korfmann A, Bergmann MM. Recruitment procedures of EPIC-Germany. European Investigation into Cancer and Nutrition. *Ann Nutr Metab* 1999;43:205-15 (PubMed PMID: 10592369).
10. Tuomilehto J, Lindström J, Eriksson JG, Valle TT, Hämäläinen H, Ilanne-Parikka P, Keinänen-Kiukaanniemi S, Laakso M, Louheranta A, Rastas M, Salminen V, Uusitupa M; Finnish Diabetes Prevention Study Group. Prevention of type 2 diabetes mellitus by changes in lifestyle among subjects with impaired glucose tolerance. *N Engl J Med* 2001;344:1343-50 (PubMed PMID: 11333990).
11. Lindström J, Peltonen M, Eriksson JG, Ilanne-Parikka P, Aunola S, Keinänen-Kiukaanniemi S, Uusitupa M, Tuomilehto J; Finnish Diabetes Prevention Study (DPS). Improved lifestyle and decreased diabetes risk over 13 years: long-term follow-up of the randomised Finnish Diabetes Prevention Study (DPS). *Diabetologia* 2013;56:284-93 (PubMed PMID: 23093136).
12. Takkunen MJ, Schwab US, de Mello VD, Eriksson JG, Lindström J, Tuomilehto J, Uusitupa MI; DPS Study Group. Longitudinal associations of serum fatty acid composition with type 2 diabetes risk and markers of insulin secretion and sensitivity in the Finnish Diabetes Prevention Study. *Eur J Nutr* 2016;55:967-79 (PubMed PMID: 25930966).
13. Kannel WB, Feinleib M, McNamara PM, Garrison RJ, Castelli WP. An investigation of coronary heart disease in families. The Framingham offspring study. *Am J Epidemiol* 1979;110:281-90 (PubMed PMID: 474565).
14. Ninomiya T, Nagata M, Hata J, Hirakawa Y, Ozawa M, Yoshida D, Ohara T, Kishimoto H, Mukai N, Fukuhara M, Kitazono T, Kiyohara Y. Association between ratio of serum eicosapentaenoic acid to arachidonic acid and risk of cardiovascular disease: the Hisayama Study. *Atherosclerosis*.2013;231:261-7 (PubMed PMID: 24267237).
15. Ozawa A, Takayanagi K, Fujita T, Hirai A, Hamazaki T, Terano T, Tamura Y, Kumagai A. Determination of long chain fatty acids in human total plasma lipids using gas chromatography [in Japanese]. *Jpn Analyst* 1982;31:87-91.
16. Ferrucci L, Bandinelli S, Benvenuti E, Di Iorio A, Macchi C, Harris TB, Guralnik JM. Subsystems contributing to the decline in ability to walk: bridging the gap between epidemiology and geriatric practice in the InCHIANTI study. *J Am Geriatr Soc* 2000;48:1618-25 (PubMed PMID: 11129752).
17. Ferrucci L, Cherubini A, Bandinelli S, Bartali B, Corsi A, Lauretani F, Martin A, Andres-Lacueva C, Senin U, Guralnik JM. Relationship of plasma polyunsaturated fatty acids to circulating inflammatory markers. *J Clin Endocrinol Metab* 2006;91:439-46 (PubMed PMID: 16234304).
18. Rodríguez-Palmero M, Lopez-Sabater MC, Castellote-Bargallo AI, De la Torre-Boronat MC, Rivero-Urgell M. Comparison of two methods for the determination of fatty acid profiles in plasma and erythrocytes. *J Chromatogr A* 1998;793:435-40 (PubMed PMID: 9474792).
19. Salonen JT. Is there a continuing need for longitudinal epidemiologic research? The Kuopio Ischaemic Heart Disease Risk Factor Study. *Ann Clin Res* 1988;20:46-50 (PubMed PMID: 3408213).

20. Sachdev PS, Brodaty H, Reppermund S, Kochan NA, Trollor JN, Draper B, Slavin MJ, Crawford J, Kang K, Broe GA, Mather KA, Lux O; Memory and Ageing Study Team. The Sydney Memory and Ageing Study (MAS): methodology and baseline medical and neuropsychiatric characteristics of an elderly epidemiological non-demented cohort of Australians aged 70-90 years. *Int Psychogeriatr* 2010;22:1248-64 (PubMed PMID: 20637138).
21. Quehenberger O, Armando AM, Dennis EA. High sensitivity quantitative lipidomics analysis of fatty acids in biological samples by gas chromatography-mass spectrometry. *Biochim Biophys Acta* 2011;1811:648-56 (PubMed PMID: 21787881).
22. Bild DE, Bluemke DA, Burke GL, Detrano R, Diez Roux AV, Folsom AR, Greenland P, Jacob DR Jr, Kronmal R, Liu K, Nelson JC, O'Leary D, Saad MF, Shea S, Szklo M, Tracy RP. Multi-Ethnic Study of Atherosclerosis: Objectives and design. *Am J Epidemiol* 2002;156:871-81 (PubMed PMID: 12397006).
23. Cao J, Schwichtenberg KA, Hanson NQ, Tsai MY. Incorporation and clearance of omega-3 fatty acids in erythrocyte membranes and plasma phospholipids. *Clin Chem* 2006;52:2265-72 (PubMed PMID: 17053155).
24. Mahendran Y, Ågren J, Uusitupa M, Cederberg H, Vangipurapu J, Stančáková A, Schwab U, Kuusisto J, Laakso M. Association of erythrocyte membrane fatty acids with changes in glycemia and risk of type 2 diabetes. *Am J Clin Nutr* 2014;99:79-85 (PubMed PMID: 24153340).
25. Lankinen MA, Stančáková A, Uusitupa M, Ågren J, Pihlajamäki J, Kuusisto J, Schwab U, Laakso M. Plasma fatty acids as predictors of glycaemia and type 2 diabetes. *Diabetologia* 2015;58:2533-44 (PubMed PMID: 26277381).
26. Laakso M, Kuusisto J, Stančáková A, Kuulasmaa T, Pajukanta P, Lusis AJ, Collins FS, Mohlke KL, Boehnke M. The Metabolic Syndrome in Men study: a resource for studies of metabolic and cardiovascular diseases. *J Lipid Res* 2017;58:481-93 (PubMed PMID: 28119442).
27. Ye X, Yu Z, Li H, Franco OH, Liu Y, Lin X. Distributions of C-reactive protein and its association with metabolic syndrome in middle-aged and older Chinese people. *J Am Coll Cardiol* 2007;49:1798-805 (PubMed PMID: 17466231).
28. Zhang G, Sun Q, Hu FB, Ye X, Yu Z, Zong G, Li H, Zhou Y, Lin X. Erythrocyte n-3 fatty acids and metabolic syndrome in middle-aged and older Chinese. *J Clin Endocrinol Metab* 2012;97:E973-7 (PubMed PMID: 22456621).
29. Zong G, Zhu J, Sun L, Ye X, Lu L, Jin Q, Zheng H, Yu Z, Zhu Z, Li H, Sun Q, Lin X. Associations of erythrocyte fatty acids in the de novo lipogenesis pathway with risk of metabolic syndrome in a cohort study of middle-aged and older Chinese. *Am J Clin Nutr* 2013;98:319-26 (PudMed PMID: 23803879).
30. Lind L, Fors N, Hall J, Marttala K, Stenborg A. A comparison of three different methods to evaluate endothelium-dependent vasodilation in the elderly: the Prospective Investigation of the Vasculature in Uppsala Seniors (PIVUS) study. *Arterioscler Thromb Vasc Biol* 2005;25:2368-75 (PubMed PMID: 16141402).
31. Hedstrand H. A study of middle-aged men with particular reference to risk factors for cardiovascular disease. *Ups J Med Sci Suppl* 1975;19:1-61 (PubMed PMID: 1216390).
32. Shumaker SA, Reboussin BA, Espeland MA, Rapp SR, McBee WL, Dailey M, Bowen D, Terrell T, Jones BN. The Women's Health Initiative Memory Study (WHIMS): a trial of the effect of estrogen therapy in preventing and slowing the progression of dementia. *Control Clin Trials* 1998;19:604-21 (PubMed PMID: 9875839).
33. Espeland MA, Rapp SR, Shumaker SA, Brunner R, Manson JE, Sherwin BB, Hsia J, Margolis KL, Hogan PE, Wallace R, Dailey M, Freeman R, Hays J; Women's Health Initiative Memory Study. Conjugated equine estrogens and global cognitive function in postmenopausal women: Women's Health Initiative Memory Study. *JAMA* 2004;291:2959-68 (PubMed PMID: 15213207).
34. Pottala JV, Espeland MA, Polreis J, Robinson J, Harris WS. Correcting the effects of -20 °C storage and aliquot size on erythrocyte fatty acid content in the Women's Health Initiative. *Lipids* 2012;47:835-46 (PubMed PMID: 22782370).
35. 3C Study Group. Vascular factors and risk of dementia: design of the Three-City Study and baseline characteristics of the study population. *Neuroepidemiology* 2003;22:316-25 (PubMed PMID: 14598854).
36. Peuchant E, Wolff R, Salles C, Jensen R. One-step extraction of human erythrocyte lipids allowing rapid determination of fatty acid composition. *Anal Biochem* 1989;181:341-4 (PubMed PMID: 2817399).

Supplementary Table S1. Invited studies not participating in the analysis

Study	Country	Reference
<u>Ineligible due to lack of exposure or outcome data:</u>		
Akershus Cardiac Examination 1950 Study (ACE1950)	Norway	Chandra A, Røsjø H, Eide IA, Vigen T, Ihle-Hansen H, Orstad EB, Rønning OM, Lyngbakken MN, Berge T, Schmidt EB, Omland T, Tveit A, Svensson M. Plasma marine n-3 polyunsaturated fatty acids and cardiovascular risk factors: data from the ACE 1950 study. *Eur J Nutr* 2020;59:1505-15 (PubMed PMID: 31123865).
Age, Gene/Environment Susceptibility Reykjavik Study (AGES-Reykjavik)	Iceland	Harris TB, Song X, Reinders I, Lang TF, Garcia ME, Siggeirsdottir K, Sigurdsson S, Gudnason V, Eiriksdottir G, Sigurdsson G, Steingrimsdottir L, Aspelund T, Brouwer IA, Murphy RA. Plasma phospholipid fatty acids and fish-oil consumption in relation to osteoporotic fracture risk in older adults: the Age, Gene/Environment Susceptibility Study. *Am J Clin Nutr* 2015;101:947-55 (PubMed PMID: 25787995).
Canadian Study of Health and Aging (CSHA)	Canada	Canadian study of health and aging: study methods and prevalence of dementia. *CMAJ* 1994;150:899-913 (PubMed PMID: 8131123).
Case-control study of Israelis	Israel	-
Costa Rica Study on Adults (CRS)	Costa Rica	Campos H, Siles X. Siesta and the risk of coronary heart disease: results from a population-based, case-control study in Costa Rica. *Int J Epidemiol* 2000;29:429-37 (PubMed PMID: 10869314).
Diet, Cancer and Health Study (DCH)	Denmark	Nielsen MS, Schmidt EB, Stegger J, Gorst-Rasmussen A, Tjonneland A, Overvad K. Adipose tissue arachidonic acid content is associated with the risk of myocardial infarction: a Danish case-cohort study. *Atherosclerosis* 2013;227:386-90 (PubMed PMID: 23390891).
Folic Acid and Carotid Intima-media Thickness (FACIT) trial	Netherlands	Dullemeijer C, Durga J, Brouwer IA, van de Rest O, Kok FJ, Brummer RJ, van Boxtel MP, Verhoef P. n 3 fatty acid proportions in plasma and cognitive performance in older adults. *Am J Clin Nutr* 2007;86:1479-85. (PubMed PMID: 17991662).
European Prospective Investigation into Cancer and Nutrition study – InterAct (EPIC-InterAct)	Denmark, France, Germany, Italy, Netherlands, Spain, Sweden, and United Kingdom	Forouhi NG, Koulman A, Sharp SJ, Imamura F, Kröger J, Schulze MB, Crowe FL, Huerta JM, Guevara M, Beulens JW, van Woudenbergh GJ, Wang L, Summerhill K, Griffin JL, Feskens EJ, Amiano P, Boeing H, Clavel-Chapelon F, Dartois L, Fagherazzi G, Franks PW, Gonzalez C, Jakobsen MU, Kaaks R, Key TJ, Khaw KT, Kühn T, Mattiello A, Nilsson PM, Overvad K, Pala V, Palli D, Quirós JR, Rolandsson O, Roswall N, Sacerdote C, Sánchez MJ, Slimani N, Spijkerman AM, Tjonneland A, Tormo MJ, Tumino R, van der A DL, van der Schouw YT, Langenberg C, Riboli E, Wareham NJ. Differences in the prospective association between individual plasma phospholipid saturated fatty acids and incident type 2 diabetes: the EPIC-InterAct case-cohort study. *Lancet Diabetes Endocrinol* 2014;2:810-8 (PubMed PMID: 25107467).

European Multicenter Case-control Study on Antioxidants, Myocardial Infarction and Breast Cancer (EURAMIC)	Finland, Germany, Israel, Netherlands, Russia, United Kingdom, Spain, Switzerland	Guallar E, Aro A, Jiménez FJ, Martín-Moreno JM, Salminen I, van't Veer P, Kardinaal AF, Gómez-Aracena J, Martin BC, Kohlmeier L, Kark JD, Mazaev VP, Ringstad J, Guillén J, Riemersma RA, Huttunen JK, Thamm M, Kok FJ. Omega-3 fatty acids in adipose tissue and risk of myocardial infarction: the EURAMIC study. *Arterioscler Thromb Vasc Biol* 1999;19:1111-8 (PubMed PMID: 10195943).
European Action on Secondary Prevention through Intervention to Reduce Events (EUROASPIRE)	Finland	Erkkilä AT, Lehto S, Pyörälä K, Uusitupa MI. n-3 Fatty acids and 5-y risks of death and cardiovascular disease events in patients with coronary artery disease. *Am J Clin Nutr* 2003;78:65-71 (PubMed PMID: 12816772).
Guangdong Coronary Artery Disease Cohort (GCADC)	China	Li Z, Zhang Y, Su D, Lv X, Wang M, Ding D, Ma J, Xia M, Wang D, Yang Y, Qiu J, Hu G, Ling W. The opposite associations of long-chain versus very long-chain monounsaturated fatty acids with mortality among patients with coronary artery disease. *Heart* 2014;100:1597-605 (PubMed PMID: 24957531).
Insulin Resistance Atherosclerosis Study (IRAS)	USA	Santaren ID, Watkins SM, Liese AD, Wagenknecht LE, Rewers MJ, Haffner SM, Lorenzo C, Hanley AJ. Serum pentadecanoic acid (15:0), a short-term marker of dairy food intake, is inversely associated with incident type 2 diabetes and its underlying disorders. *Am J Clin Nutr* 2014;100:1532-40 (PubMed PMID: 25411288).
Japan Circulatory Risk in Communities Study (CIRCS)	Japan	Yamagishi K, Ikeda A, Chei CL, Noda H, Umesawa M, Cui R, Muraki I, Ohira T, Imano H, Sankai T, Okada T, Tanigawa T, Kitamura A, Kiyama M, Iso H; CIRCS Investigators. Serum α-linolenic and other ω-3 fatty acids, and risk of disabling dementia: Community-based nested case-control study. *Clin Nutr* 2017;36:793-97 (PubMed PMID: 27265182).
Ludwigshafen Risk and Cardiovascular Health Study (LURIC)	Germany	Kleber ME, Delgado GE, Lorkowski S, März W, von Schacky C. Omega-3 fatty acids and mortality in patients referred for coronary angiography. The Ludwigshafen Risk and Cardiovascular Health Study. *Atherosclerosis* 2016;252:175-81 (PubMed PMID: 27397734).
Melbourne Collaborative Cohort Study (MCCS)	Australia	Hodge AM, English DR, O'Dea K, Sinclair AJ, Makrides M, Gibson RA, Giles GG. Plasma phospholipid and dietary fatty acids as predictors of type 2 diabetes: interpreting the role of linoleic acid. *Am J Clin Nutr* 2007;86:189-97 (PubMed PMID: 17616780).
Northern Sweden Health and Disease Study (NSHDS)	Sweden	Hallmans G, Agren A, Johansson G, Johansson A, Stegmayr B, Jansson JH, Lindahl B, Rolandsson O, Söderberg S, Nilsson M, Johansson I, Weinehall L. Cardiovascular disease and diabetes in the Northern Sweden Health and Disease Study Cohort - evaluation of risk factors and their interactions. *Scand J Public Health Suppl* 2003;61:18-24 (PubMed PMID: 14660243).
Norwegian case-control study	Norway	Pedersen JI, Ringstad J, Almendingen K, Haugen TS, Stensvold I, Thelle DS. Adipose tissue fatty acids and risk of myocardial infarction--a case-control study. *Eur J Clin Nutr* 2000;54:618-25 (PubMed PMID: 10951510).
Older Australian Twins Study (OATS)	Australia	Sachdev PS, Lammel A, Trollor JN, Lee T, Wright MJ, Ames D, Wen W, Martin NG, Brodaty H, Schofield PR; OATS research team. A comprehensive neuropsychiatric study of elderly

		twins: the Older Australian Twins Study. *Twin Res Hum Genet* 2009;12:573-82 (PubMed PMID: 19943720).
Physician's Health Study (PHS)	USA	Christen WG, Gaziano JM, Hennekens CH. Design of Physicians' Health Study II--a randomized trial of beta-carotene, vitamins E and C, and multivitamins, in prevention of cancer, cardiovascular disease, and eye disease, and review of results of completed trials. *Ann Epidemiol* 2000;10:125-34 (PubMed PMID: 10691066).
Scottish Heart Health Extended Cohort Study (SHHEC)	Scotland	Tunstall-Pedoe H, Woodward M, Tavendale R, A'Brook R, McCluskey MK. Comparison of the prediction by 27 different factors of coronary heart disease and death in men and women of the Scottish Heart Health Study: cohort study. *BMJ* 1997;315:722-9 (PubMed PMID: 9314758).
Singapore Chinese Health Study (SCHS)	Singapore	Hankin JH, Stram DO, Arakawa K, Park S, Low SH, Lee HP, Yu MC. Singapore Chinese Health Study: development, validation, and calibration of the quantitative food frequency questionnaire. *Nutr Cancer* 2001;39:187-95 (PubMed PMID: 11759279).
Swedish 60-year-olds cohort (60YO)	Sweden	Halldin M, Rosell M, de Faire U, Hellénius ML. The metabolic syndrome: prevalence and association to leisure-time and work-related physical activity in 60-year-old men and women. *Nutr Metab Cardiovasc Dis* 2007;17:349-57 (PubMed PMID: 17562572).
Declined to participate due to very limited number of incident CKD case:		
Health Professionals Follow-up Study (HPFS)	USA	Malik VS, Chiuve SE, Campos H, Rimm EB, Mozaffarian D, Hu FB, Sun Q. Circulating Very-Long-Chain Saturated Fatty Acids and Incident Coronary Heart Disease in US Men and Women. *Circulation* 2015;132:260-8 (PubMed PMID: 26048094).
Hunter Community Study (HCS)	Australia	Abbott KA, Burrows TL, Thota RN, Alex A, Acharya S, Attia J, McEvoy M, Garg ML. Association between plasma phospholipid omega-3 polyunsaturated fatty acids and type 2 diabetes is sex dependent: The Hunter Community Study. *Clin Nutr* 2020;39:1059-66 (PubMed PMID: 31023487).
Monitoring Project on Risk Factors for Chronic Diseases (MORGEN)	Netherlands	de Goede J, Verschuren WM, Boer JM, Verberne LD, Kromhout D, Geleijnse JM. N-6 and N-3 fatty acid cholesteryl esters in relation to fatal CHD in a Dutch adult population: a nested case-control study and meta-analysis. *PLoS One* 2013;8:e59408 (PubMed PMID: 23741290).
Monitoring Project on Cardiovascular Disease Risk Factors (MPCDRF)	Netherlands	de Goede J, Verschuren WM, Boer JM, Verberne LD, Kromhout D, Geleijnse JM. N-6 and N-3 fatty acid cholesteryl esters in relation to fatal CHD in a Dutch adult population: a nested case-control study and meta-analysis. *PLoS One* 2013;8:e59408 (PubMed PMID: 23741290).
Nurses' Health Study I (NHS I)	USA	Malik VS, Chiuve SE, Campos H, Rimm EB, Mozaffarian D, Hu FB, Sun Q. Circulating Very-Long-Chain Saturated Fatty Acids and Incident Coronary Heart Disease in US Men and Women. *Circulation* 2015;132:260-8 (PubMed PMID: 26048094).

<u>Declined to participate due to lack of human resource:</u>		
Coronary Artery Risk Development in Young Adults Study (CARDIA)	USA	Lemaitre RN, Tanaka T, Tang W, Manichaikul A, Foy M, Kabagambe EK, Nettleton JA, King IB, Weng LC, Bhattacharya S, Bandinelli S, Bis JC, Rich SS, Jacobs DR Jr, Cherubini A, McKnight B, Liang S, Gu X, Rice K, Laurie CC, Lumley T, Browning BL, Psaty BM, Chen YD, Friedlander Y, Djousse L, Wu JH, Siscovick DS, Uitterlinden AG, Arnett DK, Ferrucci L, Fornage M, Tsai MY, Mozaffarian D, Steffen LM. Genetic loci associated with plasma phospholipid n-3 fatty acids: a meta-analysis of genome-wide association studies from the CHARGE Consortium. *PLoS Genet* 2011;7:e1002193 (PubMed PMID: 21829377).
<u>Did not respond to invitation to participate:</u>		
Diabetes Management Through an Integrated Delivery System (DMIDS)	Taiwan	Chung HF, Long KZ, Hsu CC, Al Mamun A, Jhang HR, Shin SJ, Hwang SJ, Huang MC. Association of n-3 polyunsaturated fatty acids and inflammatory indicators with renal function decline in type 2 diabetes. *Clin Nutr* 2015;34:229-34 (PubMed PMID: 24721145).
Genetics of Lipid Lowering Drugs and Diet Network	USA	Irvin MR, Montasser ME, Kind T, Fan S, Barupal DK, Patki A, Tanner RM, Armstrong ND, Ryan KA, Claas SA, O'Connell JR, Tiwari HK, Arnett DK. Genomics of Postprandial Lipidomics in the Genetics of Lipid-Lowering Drugs and Diet Network Study. *Nutrients* 2021;13:4000 (PubMed PMID: 34836252).
Multiple Risk Factor Intervention Trial	USA	Simon JA, Fong J, Bernert JT Jr, Browner WS. Serum fatty acids and the risk of fatal cancer. MRFIT Research Group. Multiple Risk Factor Intervention Trial. *Am J Epidemiol* 1998;148:854-8 (PubMed PMID: 9801015).

Supplementary Table S2. Distribution of categorical covariates at baseline[a]

Study	n	Race	Smoking	Alcohol consumption	Education	Occupation	Physical activity	Prevalent diseases	Medication use
AOC	2026	98.8 (Caucasian)<br>0.8 (Asian)<br>0.2 (Black)<br>0.1 (Others)	16.7 (never)<br>67.3 (former)<br>16.0 (current)	21.8 (none)<br>45.7 (<1 drink/d)<br>15.7 (1-2 drinks/d)<br>16.8 (>2 drinks/d)	20.9 (< high school)<br>66.1 (high school)<br>12.4 (> high school)	n/a	28.8 (quartile 1)<br>21.0 (quartile 2)<br>24.9 (quartile 3)<br>24.9 (quartile 4)	Diabetes: 18.6<br>CHD: 100<br>Albuminuria: n/a	ACEi/ARBs: 41.6<br>other HTD: 45.4<br>Any HTD: 87.1<br>LLD: 86.2
ARIC	3653	100 (Caucasian)	38.0 (never)<br>40.1 (former)<br>21.9 (current)	14.9 (none)<br>34.6 (<1 drink/d)<br>15.9 (1-2 drinks/d)<br>7.6 (>2 drinks/d)	6.0 (< high school)<br>34.4 (high school)<br>59.6 (> high school)	n/a	physical activity index (mean ± SD): 2.6 ± 0.8	Diabetes: 6.7<br>CHD: 3.6<br>Albuminuria: n/a	ACEi/ARBs: n/a<br>other HTD: n/a<br>Any HTD: 16.3<br>LLD: 0.1
CCCC	1074	100 (Asian)	54.5 (never)<br>5.7 (former)<br>39.9 (current)	34.1 (regular intake)	95.6 (< high school)<br>4.4 (high school)<br>0.0 (> high school)	12.3 (clerical)<br>38.6 (labour)<br>49.2 (others)	15.8 (regular exercise)	Diabetes: 16.9<br>CHD: 3.5<br>Albuminuria: n/a	ACEi/ARBs: n/a<br>other HTD: n/a<br>Any HTD: 12.5<br>LLD: n/a
CHS	1608	86.5 (Caucasian)<br>13.5 (Others)	47.9 (never)<br>41.7 (former)<br>8.7 (current)	52.2 (none)<br>33.3 (<1 drink/d)<br>7.7 (1-2 drinks/d)<br>6.7 (>2 drinks/d)	23.6 (< high school)<br>27.5 (high school)<br>48.6 (> high school)	n/a	total number of blocks walked over 1 week (mean ± SD): 49.3 ± 76.2	Diabetes: 13.1<br>CHD: 16.3<br>Albuminuria: n/a	ACEi/ARBs: n/a<br>other HTD: n/a<br>Any HTD: 34.9<br>LLD: 6.9
EPIC-Norfolk	926	100 (Caucasian)	51.8 (never)<br>40.6 (former)<br>7.6 (current)	21.9 (none)<br>52.0 (<1 drink/d)<br>18.0 (1-2 drinks/d)<br>8.1 (>2 drinks/d)	28.0 (No)<br>8.2 (O Level)<br>46.8 (A Level)<br>17.0 (College or higher)	9.4 (general employees)<br>42.5 (manager)<br>35.3 (skilled)<br>10.3 (semi-skilled)<br>2.5 (non-skilled)	22.3 (inactive)<br>31.5 (moderately inactive)<br>25.8 (moderately active)<br>20.5 (active)	Diabetes: 2.0<br>CHD: 2.4<br>Albuminuria: n/a	ACEi/ARBs: n/a<br>other HTD: n/a<br>Any HTD: 14.6<br>LLD: 1.5
EPIC-Potsdam	253	100 (Caucasian)	50.6 (never)<br>30.0 (former)<br>19.4 (current)	2.8 (none)<br>60.5 (<1 drink/d)<br>21.0 (1-2 drinks/d)<br>15.8 (>2 drinks/d)	32.0 (< high school)<br>18.6 (high school)<br>49.4 (> high school)	69.2 (full time)<br>9.1 (part time)<br>10.7 (unemployed)<br>11.1 (retirement/ invalidity pension)	26.1 (0 h/week)<br>21.0 (0.5-1.5 h/week)<br>25.3 (2.0-3.5 h/week)<br>27.7 (>3.5 h/week)	Diabetes: 6.3<br>CHD: 7.1<br>Albuminuria: n/a	ACEi/ARBs: n/a<br>other HTD: n/a<br>Any HTD: 15.0<br>LLD: 5.5

FDPS	355	100 (Caucasian)	60.3 (never)<br>30.1 (former)<br>9.6 (current)	56.9 (none)<br>29.3 (<1 drink/d)<br>7.9 (1-2 drinks/d)<br>5.9 (>2 drinks/d)	36.6 (< high school)<br>52.7 (high school)<br>10.7 (> high school)	n/a (all retired)	mean ± SD: 7.2 ± 5.9 hours/week	Diabetes: 0<br>CHD: 2.3<br>Albuminuria: n/a	ACEi/ARBs: 7.0<br>other HTD: 21.5<br>Any HTD: 28.5<br>LLD: 2.3
FHS	1895	100 (Caucasian)	83.9 (never or former)<br>8.7 (current)	22.2 (none)<br>44.9 (<1 drink/d)<br>20.3 (1-2 drinks/d)<br>5.3 (>2 drinks/d)	2.4 (< high school)<br>22.9 (high school)<br>20.3 (Some College/Voc School)<br>46.8 (≥ college)	45.0 (employed)<br>7.3 (homemaker)<br>1.1 (disabled)<br>1.4 (unemployed)<br>37.6 (retired)	mean ± SD: 42.2 ± 6.0 METs/day	Diabetes: 11.1<br>CHD: 7.3<br>Albuminuria: 5.6	ACEi/ARBs: n/a<br>other HTD: n/a<br>Any HTD: 40.0<br>LLD: 36.4
Hisayama	2713	100 (Asian)	62.5 (never)<br>21.5 (former)<br>16.0 (current)	43.2 (none)<br>30.9 (<1 drink/d)<br>9.3 (1-2 drinks/d)<br>16.6 (>2 drinks/d)	n/a	n/a	42.6 (none)<br>29.8 (walking)<br>16.5 (sometimes)<br>11.1 (regular)	Diabetes: 16.8<br>CHD: 1.3<br>Albuminuria: 17.5	ACEi/ARBs: 9.3<br>other HTD: 21.4<br>Any HTD: 22.0<br>LLD: 9.7
InCHIANTI	830	100 (Caucasian)	54.3 (never)<br>24.1 (former)<br>21.6 (current)	21.1 (none)<br>41.8 (<1 drink/d)<br>16.4 (1-2 drinks/d)<br>20.7 (>2 drinks/d)	77.7 (< high school)<br>13.4 (high school)<br>8.9 (> high school)	n/a	10.5 (hardly any to most sitting/some walking)<br>41.8 (Light exercise 2-4 hrs/week)<br>38.2 (Moderate 1-2hrs or light >4hrs/wk)<br>9.6 (Moderate exercise > 3hrs/wk / Intense exercise many time/wk, walks >5 km/day for >5 days/wk for ≥5 yrs)	Diabetes: 10.2<br>CHD: 8.9<br>Albuminuria: n/a	ACEi/ARBs: 16.5<br>other HTD: n/a<br>Any HTD: 27.7<br>LLD: 3.1
KIHD	1082	100 (Caucasian)	41.1 (never)<br>37.1 (former)<br>21.8 (current)	13.2 (none)<br>68.2 (<1 drink/d)<br>11.9 (1-2 drinks/d)<br>6.6 (>2 drinks/d)	50.7 (< high school)<br>42.2 (high school)<br>6.8 (> high school)	45.3 (clerical)<br>54.7 (others)	mean ± SD: 149±171 kcal/day	Diabetes: 2.7<br>CHD: 19.1<br>Albuminuria: n/a	ACEi/ARBs: n/a<br>other HTD: n/a<br>Any HTD: 17.7<br>LLD: 0.6

MAS	395	n/a (but majority is Caucasian)	47.1 (never)<br>50.1 (former)<br>2.8 (current)	8.4 (none)<br>78.0 (1-2 drinks/d)<br>13.7 (>2 drinks/d)	1.3 (< high school)<br>61.8 (high school)<br>37.0 (> high school)	all retired	8.9 (none)<br>66.8 (1-2 activities)<br>24.3 (≥3 activities)	Diabetes: 10.4<br>CHD: 39.4<br>Albuminuria: n/a	ACEi/ARBs: n/a<br>other HTD: n/a<br>Any HTD: 58.2<br>LLD: 52.7
MESA	1682	26.8 (Caucasian)<br>25.8 (Asian)<br>22.7 (Black)<br>24.7 (Hispanic)	55.5 (never)<br>30.7 (former)<br>13.8 (current)	51.5 (none)<br>39.5 (<1 drink/d)<br>5.0 (1-2 drinks/d)<br>4.0 (>2 drinks/d)	0.8 (no schooling)<br>9.6 (grades 1-8)<br>6.0 (grade 9-11)<br>17.9 (completed high school/GED)<br>15.5 (some college, but no degree)<br>7.5 (technical school certificate)<br>6.0 (associate degree)<br>17.7 (bachelor's degree)<br>18.9 (graduate or professional school)	9.7 (homemaker)<br>49.7 (employed full time)<br>10.5 (employed part-time)<br>0.6 (employed on-leave, Health)<br>0.3 (employed on-leave, non-health)<br>1.2 (unemployed <6 months)<br>0.9 (unemployed >6 months)<br>18.3 (retired not working)<br>4.8 (retired working)<br>4.1 (retired volunteering)	mean ± SD: 5447 ± 4339 METs/day	Diabetes: 11.2<br>CHD: 6.7<br>Albuminuria: 6.5	ACEi/ARBs: 13.1<br>other HTD: n/a<br>Any HTD: n/a<br>LLD: 13.9
METSIM	1152	100 (Caucasian)	45.6 (never)<br>15.9 (former)<br>38.5 (current)	20.1 (none)<br>39.3 (<1 drink/d)<br>25.5 (1-2 drinks/d)<br>15.0 (>2 drinks/d)	n/a	n/a	4.3 (none)<br>26.1 (occasional)<br>17.5 (regular, <2 times/week)<br>52.0 (regular, ≥3 times/week)	Diabetes: 3.6<br>CHD: 0<br>Albuminuria: n/a	ACEi/ARBs: 8.9<br>other HTD: 3.3<br>Any HTD: 12.2<br>LLD: 13.9
NHAPC	2046	100 (Asian)	72.3 (never or former)<br>27.7 (current)	74.4 (never or former)<br>25.6 (current)	81.0 (< high school)<br>12.8 (high school)<br>6.3 (> high school)	22.8 (clerical)<br>68.9 (labour)<br>8.3 (others)	Frequency (%):<br>6.8 (low)<br>38.6 (median)<br>54.6 (high)	Diabetes: 12.0<br>CHD: 6.1<br>Albuminuria: n/a	ACEi/ARBs: n/a<br>other HTD: n/a<br>Any HTD: 25.2<br>LLD: 6.0
PIVUS	893	100 (Caucasian)	75.3 (never)	n/a	24.9 (< high school)	64.9 (clerical)	Frequency (%):	Diabetes: 8.2	ACEi/ARBs: 7.3

			13.6 (former)<br>11.2 (current)		57.2 (high school)<br>17.9 (> high school)	35.0 (others)	40.1 (level 1, low)<br>27.3 (level 2)<br>14.1 (level 3)<br>18.6 (level 4, high)	CHD: 7.6<br>Albuminuria: n/a	other HTD: 21.6<br>Any HTD: 28.9<br>LLD: 14.9
ULSAM	1055	100 (Caucasian)	79.1 (never or former)<br>20.9 (current)	5.1 (5cl spirits in last 24 hr)	0.0 (< high school)<br>8.5 (high school)<br>91.5 (> high school)	n/a	Frequency (%):<br>14.7 (sedentary)<br>35.4 (moderate)<br>43.9 (strenuous)<br>5.0 (athletic)	Diabetes: 1.4<br>CHD: 0.8<br>Albuminuria: n/a	ACEi/ARBs: n/a<br>other HTD: n/a<br>Any HTD: 15.7<br>LLD: 10.0
WHIMS	1686	93.9 (Caucasian)<br>4.1 (Black)<br>1.9 (Hispanic)	57.9 (never)<br>37.4 (former)<br>4.3 (current)	39.0 (none)<br>47.5 (<1 drink/d)<br>9.2 (1-2 drinks/d)<br>4.3 (>2 drinks/d)	3.1 (< high school)<br>21.3 (high school)<br>38.2 (some college)<br>37.5 (college or higher)	11.50 (homemaker)<br>36.2 (manager)<br>28.8 (technician)<br>8.4 (service)<br>1.8 (labour)<br>11.5 (other)<br>1.7 (missing)	mean ± SD: 12.5 ± 13.1 METs/week<br><br>Frequency (%):<br>20.3 (<1.9 METs/week)<br>20.4 (1.9-7.3 METs/week)<br>26.6 (7.3-16.4 METs/week)<br>28.8 (>16.4 METs/week)	Diabetes: 3.0<br>CHD: 14.5<br>Albuminuria: n/a	ACEi/ARBs: n/a<br>other HTD: n/a<br>Any HTD: 25.0<br>LLD: 15.8
3C - Total plasma sample	1019	100 (Caucasian)	65.4 (never)<br>29.7 (former)<br>4.9 (current)	19.3 (none)<br>23.7 (<1 drink/d)<br>41.2 (1-2 drinks/d)<br>15.8 (>2 drinks/d)	59.8 (< high school)<br>22.2 (high school)<br>18.0 (> high school)	n/a	Frequency (%):<br>30.3 (regular exercise)	Diabetes: 9.0<br>CHD: 11.4<br>Albuminuria: n/a	ACEi/ARBs: 21.6<br>other HTD: 42.4<br>Any HTD: 53.2<br>LLD: 30.9
3C - Erythrocyte phospholipid sample	373	100 (Caucasian)	64.5 (never)<br>28.2 (former)<br>7.3 (current)	20.9 (none)<br>23.1 (<1 drink/d)<br>40.5 (1-2 drinks/d)<br>15.6 (>2 drinks/d)	61.7 (< high school)<br>23.1 (high school)<br>15.3 (> high school)	n/a	Frequency (%):<br>27.9 (regular exercise)	Diabetes: 12.6<br>CHD: 12.6<br>Albuminuria: n/a	ACEi/ARBs: 20.6<br>other HTD: 38.1<br>Any HTD: 49.6<br>LLD: 27.9

Abbreviations: ACEi, angiotensin-converting enzyme inhibitor; AOC, Alpha Omega Cohort; ARB, angiotensin receptor blockers; ARIC, Atherosclerosis Risk in Communities Study; CCCC, Chin-Shan Community Cardiovascular Cohort Study; CHD, coronary heart disease; CHS, Cardiovascular Health Study; CKD, chronic kidney disease; EPIC-Norfolk, European Prospective Investigation into Cancer and Nutrition (Norfolk); EPIC-Potsdam, European Prospective Investigation into Cancer and Nutrition (Potsdam); FDPS, Finnish Diabetes Prevention Study; FHS, Framingham Heart Study; InCHIANTI, Invecchiare in Chianti Study; HTD, hypertension drug; KIHD, Kuopio Ischaemic Heart Disease Risk Factor Study; LLD, lipid-lowering drug; MAS, Sydney Memory and Ageing Study; MESA, Multi-Ethnic Study of Atherosclerosis; MET, metabolic equivalent; METSIM, Metabolic Syndrome in Men; n/a, data not available; NHAPC, Nutrition and Health of Aging Populations in

China; PIVUS, Prospective Investigation of the Vasculature in Uppsala Seniors; WHIMS, Women's Health Initiative Memory Study; ULSAM, Uppsala Longitudinal Study of Adult Men; 3C, Three City Study.

[a]For statistical analyses, covariates were pre-specified and standardised across studies with harmonised definitions and categorisation: For physical activity, the first preference is quartiles of METs or continuous METs. If METs are not available, then the second preference is the use of four categories of physical or leisure activity as defined in individual studies, or cohort-specific definitions. Prevalent diabetes mellitus was defined as treatment with oral hypoglycaemic agents or insulin, fasting glucose >126 mg/dL, or cohort-specific definitions. Prevalent coronary heart disease was defined by ICD-9 410-414, or ICD-10 I20-I25, or cohort-specific definitions. Albuminuria was defined as urinary albumin-creatinine ratio ≥30 mg/g.

Supplementary Table S3. Distribution of continuous covariates at baseline

Study	n	SBP, mm Hg (mean±SD)	DBP, mm Hg (mean±SD)	UACR, mg/g (mean±SD)	eGFR at baseline, mL/min/1.73m^2 (mean±SD)
AOC	2026	143.4±21.4	81.4±10.8	n/a	76.1±15.5
ARIC	3653	118.5±16.1	73.7±9.3	n/a	99.8±11.6
CCCC	1074	127.2±20.6	77.9±11.1	n/a	93.6±14.0
CHS	1608	134.9±19.7	71.1±11.0	n/a	72.6±9.6
EPIC-Norfolk	926	134.0±17.7	81.7±11.1	n/a	78.9±16.2
EPIC-Potsdam	253	126.5±16.4	83.5±10.6	n/a	91.7±13.9
FDPS	355	138.5±18.1	85.9±9.7	n/a	78.0±10.8
FHS	1895	127.1±16.2	74.5±9.7	11.6±30.5	85.6±11.5
Hisayama	2713	131.3±20.5	78.5±11.7	28.5±88.2	80.6±8.9
InCHIANTI	830	143.1±20.8	82.4±9.2	n/a	76.9±16.2
KIHD	1082	131.3±15.2	87.7±9.9	n/a	89.8±11.6
MAS	395	146.4±19.4	84.0±10.4	n/a	79.4±9.6
MESA	1682	122.4±19.9	71.7±10.0	14.5±68.0	82.7±12.8
METSIM	1152	134±15	87±9	n/a	90.8±10.3
NHAPC	2046	139.5±22.3	79.9±10.7	n/a	82.2±10.6
PIVUS	893	146.5±20.3	68.8±9.1	n/a	82.9±11.0
ULSAM	1055	133±11	84±11	n/a	95.2±11.1
WHIMS	1686	129.9±16.7	74.8±8.7	n/a	80.9±12.2
3C - Total plasma sample	1019	143.3±21.0	80.9±10.7	n/a	80.4±9.4
3C - Erythrocyte phospholipid sample	373	142.4±19.9	79.4±10.9	n/a	78.9±9.0

Abbreviations: AOC, Alpha Omega Cohort; ARIC, Atherosclerosis Risk in Communities Study; CCCC, Chin-Shan Community Cardiovascular Cohort Study; CHS, Cardiovascular Health Study; DBP, diastolic blood pressure; eGFR; estimated glomerular filtration rate; EPIC-Norfolk, European Prospective Investigation into Cancer and Nutrition (Norfolk); EPIC-Potsdam, European Prospective Investigation into Cancer and Nutrition (Potsdam); FDPS, Finnish Diabetes Prevention Study; FHS, Framingham Heart Study; InCHIANTI, Invecchiare in Chianti Study; KIHD, Kuopio Ischaemic Heart Disease Risk Factor Study; MAS, Memory and Ageing Study; MESA, Multi-Ethnic Study of Atherosclerosis; METSIM, Metabolic Syndrome in Men; n/a, data not available; NHAPC, Nutrition and Health of Aging Populations in China; PIVUS, Prospective Investigation of the Vasculature in Uppsala Seniors; SBP, systolic blood pressure; WHIMS, Women's Health Initiative Memory Study; UACR, Urinary albumin-creatinine ratio; ULSAM , Uppsala Longitudinal Study of Adult Men; 3C, Three City Study.

Supplementary Table S4. Number of incident cases for each of the primary and secondary outcomes across the 19 participating cohorts

Study	Biomarker Compartment	Primary outcome of incident CKD			Sensitivity analysis with stringent definition of incident CKD with >25% decrease in eGFR compared to baseline			Secondary outcome of ≥40% decrease in eGFR[b]		
		Total n	no. of case (%)	Annual rate (%)[d]	Total n	no. of case (%)	Annual rate (%)[d]	Total n	no. of case (%)	Annual rate (%)[d]
AOC	Cholesterol esters	2026	457 (22.6)	6.6	2026	306 (15.1)	4.4	2407	264 (11.0)	3.2
ARIC	Plasma phospholipids	3526	720 (20.4)	0.8	3526	687 (19.5)	0.8	3555	611 (17.2)	0.7
CCCC	Total serum	1074	187 (17.4)	17.4	1074	174 (16.2)	16.2	1718	280 (16.3)	16.3
CHS	Plasma phospholipids	1608	407 (25.3)	4.5	1608	181 (11.3)	2.0	2643	144 (5.4)	1.0
EPIC-Norfolk	Plasma phospholipids	926	73 (7.9)	0.6	926	48 (5.2)	0.4	1027	24 (2.3)	0.2
EPIC-Potsdam	Erythrocyte phospholipids	253	30 (11.9)	0.6	253	26 (10.3)	0.5	259	14 (5.4)	0.3
FDPS[a]	Total serum	355	31 (8.7)	2.2	-	-	-	-	-	-
FHS	Erythrocyte phospholipids	1895	288 (15.2)	2.6	1895	170 (9.0)	1.5	2041	64 (3.1)	0.5
Hisayama	Total serum	2713	697 (25.7)	2.6	2713	457 (16.8)	1.7	2934	164 (5.6)	0.6
InCHIANTI	Total plasma	830	270 (32.5)	3.6	830	176 (21.2)	2.3	962	89 (9.3)	1.0
KIHD	Total serum	1082	61 (5.6)	0.3	1082	36 (3.3)	0.2	1091	20 (1.8)	0.1
MAS[a]	Total plasma	395	24 (6.1)	1.0	-	-	-	-	-	
MESA	Plasma phospholipids	1682	280 (16.6)	1.8	1682	176 (10.5)	1.1	1841	60 (3.3)	0.3
METSIM[a]	Erythrocyte phospholipids, plasma phospholipids, cholesterol esters	1152	17 (1.5)	0.3	-	-	-	-	-	
NHAPC	Erythrocyte phospholipids	2046	541 (26.4)	4.4	2046	298 (14.6)	2.4	2140	57 (2.7)	0.4
PIVUS	Plasma phospholipids	893	178 (19.9)	2.0	893	138 (15.5)	1.6	1016	74 (7.3)	0.7
ULSAM	Cholesterol esters	1055	137 (13.0)	0.6	1055	118 (11.2)	0.5	1055	75 (7.1)	0.3
WHIMS	Erythrocyte phospholipids	1686	444 (26.3)	1.7	1686	333 (19.8)	1.3	1785	519 (29.1)	1.9
3C	Total plasma	1019	252 (24.7)	6.5	1019	164 (16.1)	4.8	1130	258 (22.8)	5.4
3C	Erythrocyte phospholipids	373	102 (27.3)	5.9	373	75 (20.1)	3.8	422	95 (22.5)	5.4
Total[c]	-	25570	4944 (19.3)	-	23668	3399 (14.4)	-	26896	2554 (9.5)	-

Abbreviations: AOC, Alpha Omega Cohort; ARIC, Atherosclerosis Risk in Communities Study; CCCC, Chin-Shan Community Cardiovascular Cohort Study; CHS, Cardiovascular Health Study; CKD, chronic kidney disease; DHA, docosahexaenoic acid; EPIC-Norfolk, European Prospective Investigation into Cancer and Nutrition (Norfolk); EPIC-Potsdam, European Prospective Investigation into Cancer and Nutrition (Potsdam); FDPS, Finnish Diabetes Prevention Study; FHS, Framingham Heart Study; InCHIANTI, Invecchiare in Chianti Study; KIHD, Kuopio Ischaemic Heart Disease Risk Factor Study; MAS, Sydney Memory and Ageing Study; MESA, Multi-Ethnic Study of Atherosclerosis; METSIM, Metabolic Syndrome in Men; NHAPC, Nutrition and Health of Aging Populations in China; PIVUS, Prospective Investigation of the Vasculature in Uppsala Seniors; WHI, Women's Health Initiative Memory Study; ULSAM, Uppsala Longitudinal Study of Adult Men; 3C, Three City Study.

[a]FDPS, MAS and METSIM did not contribute to the sensitivity and the secondary analyse of ≥40% decline in eGFR due to very low numbers of incident cases (n ≤11).

[b]For the secondary outcomes, participants with prevalent CKD at baseline were not excluded in the analysis, resulting in a larger sample size compared to the primary outcome.

[c]The total number included the sample size for the erythrocyte phospholipid for the 3C study since this biomarker compartment was used in the overall pooled analysis.

[d]The annual rate was calculated by dividing the percentage of cases with the median follow-up duration for each cohort.

Supplementary Table S5. Baseline levels of omega-3 polyunsaturated fatty acid biomarkers

Study	Compartment	Fatty acid levels, % of total fatty acid (median [10th-90th percentiles])				
		**ALA**	**EPA**	**DPA**	**DHA**	**EPA + DPA + DHA[a]**
AOC	Cholesterol esters	0.51 (0.35 - 0.70)	1.06 (0.61 - 2.25)	0.00 (0.00 - 0.08)	0.66 (0.44 - 1.00)	1.74 (1.13 - 3.20)
ARIC	Plasma phospholipids	0.14 (0.09 - 0.21)	0.51 (0.31 - 0.83)	0.89 (0.69 - 1.12)	2.66 (1.87 - 3.93)	3.58 (3.16 - 5.57)
CCCC	Total serum	0.65 (0.46 - 0.95)	0.34 (0.21 - 0.62)	n/a	1.73 (1.09 - 2.65)	2.10 (1.36 - 3.22)*
CHS	Plasma phospholipids	0.14 (0.09 - 0.22)	0.54 (0.32 - 0.97)	0.83 (0.65 - 1.06)	2.93 (1.99 - 4.48)	4.31 (3.25 - 6.23)
EPIC-Norfolk	Plasma phospholipids	0.22 (0.14 - 0.35)	1.06 (0.60 - 2.13)	1.38 (0.97 - 1.92)	5.02 (3.37 - 7.36)	7.56 (3.21 - 11.01)
EPIC-Potsdam	Erythrocyte phospholipids	0.15 (0.11 - 0.22)	0.79 (0.50 - 1.21)	2.39 (1.83 - 2.84)	4.93 (3.50 - 6.21)	8.24 (6.23 - 9.85)
FDPS	Total serum	0.96 (0.72 - 1.36)	1.28 (0.69 - 2.73)	0.58 (0.40 - 0.80)	2.75 (1.64 - 4.36)	4.55 (2.88 - 7.66)
FHS	Erythrocyte phospholipids	0.16 (0.11 - 0.28)	0.60 (0.37 - 1.13)	2.67 (2.22 - 3.23)	4.62 (3.14 - 6.54)	7.89 (6.14 - 10.51)
Hisayama	Total serum	0.64 (0.46 - 0.94)	2.10 (1.02 - 4.04)	0.62 (0.44 - 0.90)	4.62 (3.24 - 6.52)	7.37 (4.83 - 11.17)
InCHIANTI	Total plasma	0.39 (0.26 - 0.77)	0.59 (0.42 - 0.84)	n/a	2.26 (1.41 - 3.22)	2.87 (1.91 - 4.02)*
KIHD	Total serum	0.71 (0.48 - 1.05)	1.46 (0.89 - 2.65)	0.55 (0.44 - 0.69)	2.38 (1.66 - 3.32)	4.33 (3.14 - 6.60)
MAS	Total plasma	0.31 (0.16 - 0.50)	1.86 (0.77 - 3.78)	0.45 (0.17 - 1.59)	4.00 (1.29 - 6.81)	6.54 (3.09 - 11.78)
MESA	Plasma phospholipids	0.17 (0.13 - 0.21)	0.72 (0.52 - 1.08)	0.93 (0.81 - 1.08)	3.98 (3.03 - 5.14)	5.64 (4.53 - 7.16)
METSIM	Erythrocyte phospholipids	0.18 (0.13 - 0.26)	1.42 (0.90 - 2.30)	2.59 (2.11-3.10)	6.21 (4.73 - 7.59)	10.23 (8.10 - 12.60)
METSIM	Cholesterol esters	0.99 (0.70 - 1.36)	2.16 (1.26 - 4.06)	n/a	1.03 (0.70 - 1.49)	3.18 (2.05 - 5.47)
METSIM	Plasma phospholipids	0.30 (0.19 - 0.48)	2.03 (1.19 - 3.82)	1.40 (1.15 - 1.67)	5.66 (3.84 - 7.63)	9.10 (6.55 - 12.86)
NHAPC	Erythrocyte phospholipids	0.24 (0.15 - 0.39)	0.41 (0.24 - 0.72)	1.73 (1.39 - 2.13)	4.37 (3.21 - 5.77)	6.54 (5.11 - 8.27)
PIVUS	Plasma phospholipids	0.30 (0.21 - 0.44)	1.97 (1.22-3.62)	1.13 (0.88-1.42)	5.15 (3.78 - 6.81)	8.28 (6.23 - 11.42)
ULSAM	Cholesterol esters	0.64 (0.47 - 0.87)	1.25 (0.70 - 2.14)	n/a	0.68 (0.46 - 0.96)	1.93 (1.16 - 3.05)*
WHIMS	Erythrocyte phospholipids	0.15 (0.09 - 0.26)	0.63 (0.35 - 1.22)	2.54 (2.03 - 3.1)	4.44 (2.96 - 6.43)	7.65 (5.81 - 10.24)
3C	Total plasma	0.4 (0.2 - 0.6)	0.9 (0.5 - 1.8)	0.5 (0.3 - 0.6)	2.4 (1.6 - 3.5)	3.8 (2.5 - 5.7)
3C	Erythrocyte phospholipids	0.08 (0.05 - 0.12)	0.72 (0.41 - 1.29)	1.70 (1.39 - 1.86)	4.04 (2.97 - 5.13)	6.42 (5.06 - 8.13)

Abbreviations: ALA, α-linolenic acid; AOC, Alpha Omega Cohort; ARIC, Atherosclerosis Risk in Communities Study; CCCC, Chin-Shan Community Cardiovascular Cohort Study; CHS, Cardiovascular Health Study; CKD, chronic kidney disease; DHA, docosahexaenoic acid; DPA, docosapentaenoic acid; EPA, eicosapentaenoic acid; EPIC-Norfolk, European Prospective Investigation into Cancer and Nutrition (Norfolk); EPIC-Potsdam, European Prospective Investigation into Cancer and Nutrition (Potsdam); FDPS, Finnish Diabetes Prevention Study; FHS, Framingham Heart Study; InCHIANTI, Invecchiare in Chianti Study; KIHD, Kuopio Ischaemic Heart Disease Risk Factor Study; MAS, Memory and Ageing Study; MESA, Multi-Ethnic Study of Atherosclerosis; METSIM, Metabolic Syndrome in Men; n/a, not available; NHAPC, Nutrition and Health of Aging Populations in China; PIVUS, Prospective Investigation of the Vasculature in Uppsala Seniors; WHIMS, Women's Health Initiative Memory Study; ULSAM, Uppsala Longitudinal Study of Adult Men; 3C, Three City Study.

[a]For the sum of seafood n-3 PUFA (EPA+DPA+DHA), as DPA was not available in three of the cohorts (CCCC, InCHIANTI and ULSAM), the sum in these cohorts was calculated as EPA+DHA instead.

Supplementary Table S6. Association of seafood n-3 PUFA biomarkers measured in different lipid compartments with the primary outcome of incident CKD[a]

n-3 PUFA biomarkers	Biomarker	Studies, n	Cases, n[b]	Model[c]	Per interquintile range		Quintile 5 vs. Quintile 1	
					RR (95% CI)	I^2	RR (95% CI)	I^2
**EPA**	Phospholipids	11	3080	1	0.95 (0.89 to 1.02)	0.0	0.90 (0.81 to 1.00)	0.0
				2	0.94 (0.88 to 1.01)	0.0	0.89 (0.80 to 0.99)	0.0
	Cholesterol esters	3	607	1	0.89 (0.76 to 1.04)	0.0	0.89 (0.70 to 1.14)	34.1
				2	0.88 (0.76 to 1.03)	0.0	0.91 (0.71 to 1.16)	51.0
	Total plasma/serum	7	1522	1	0.92 (0.83 to 1.03)	0.0	0.92 (0.78 to 1.08)	0.0
				2	0.93 (0.84 to 1.04)	14.9	0.92 (0.79 to 1.09)	32.7
**DPA**	Phospholipids	11	3080	1	0.92 (0.85 to 1.00)	0.0	0.91 (0.81 to 1.02)	0.0
				2	0.93 (0.85 to 1.01)	0.0	0.92 (0.82 to 1.03)	0.0
	Cholesterol esters	1	457	1	0.96 (0.82 to 1.11)	-	0.90 (0.74 to 1.10)	-
				2	0.95 (0.82 to 1.11)	-	0.90 (0.73 to 1.10)	-
	Total plasma/serum	5	1065	1	0.92 (0.80 to 1.07)	36.0	0.81 (0.65 to 1.00)	20.0
				2	0.95 (0.81 to 1.10)	40.5	0.83 (0.66 to 1.04)	35.1
**DHA**	Phospholipids	11	3080	1	0.93 (0.85 to 1.01)	2.2	0.92 (0.81 to 1.04)	0.0
				2	0.92 (0.84 to 1.01)	14.8	0.91 (0.81 to 1.03)	10.4
	Cholesterol esters	3	611	1	0.88 (0.73 to 1.05)	0.0	0.78 (0.61 to 0.98)	0.0
				2	0.87 (0.73 to 1.04)	0.0	0.77 (0.61 to 0.97)	0.0
	Total plasma/serum	7	1522	1	0.98 (0.87 to 1.11)	55.7	0.86 (0.73 to 1.02)	19.3
				2	0.98 (0.87 to 1.11)	53.6	0.87 (0.73 to 1.04)	16.8
**EPA + DPA + DHA[d]**	Phospholipids	11	3080	1	0.92 (0.85 to 1.00)	0.0	0.88 (0.78 to 1.00)	0.0
				2	0.91 (0.84 to 0.99)	0.0	0.88 (0.78 to 1.00)	0.0
	Cholesterol esters	3	606	1	0.88 (0.74 to 1.03)	0.0	0.84 (0.66 to 1.07)	37.7
				2	0.87 (0.74 to 1.03)	0.0	0.83 (0.66 to 1.06)	36.2
	Total plasma/serum	7	1522	1	0.91 (0.80 to 1.03)	51.2	0.82 (0.69 to 0.97)	0.0
				2	0.91 (0.80 to 1.03)	52.0	0.83 (0.70 to 0.99)	0.0

Abbreviations: CKD, chronic kidney disease; DHA, docosahexaenoic acid; DPA, docosapentaenoic acid; EPA, eicosapentaenoic acid; PUFA, polyunsaturated fatty acids; RR, relative risk.

[a]Incident CKD was defined as an eGFR <60 mL/min/1.73 m^2 during follow-up among participants with baseline eGFR ≥60 mL/min/1.73 m^2. Effect estimates were pooled using inverse-variance weighted meta-analysis.

[b]The small difference in number of cases was due to missing measurement for specific n-3 PUFA fatty acids in some cohorts.

[c]Model 1 adjusted for age, sex, race, clinical centre / field site, education, occupation, body mass index, smoking, alcohol intake, physical activity, prevalent coronary heart disease, and use of lipid-lowering drugs, where applicable. Model 2 adjusted for all covariates in Model 1 and additionally adjusted for prevalent diabetes mellitus, urine albumin-creatinine ratio, systolic blood pressure, use of angiotensin-converting enzyme inhibitors or angiotensin receptor blockers, and use of other anti-hypertensive drugs.

[d]For the sum of seafood n-3 PUFA (EPA+DPA+DHA), as DPA was not available in three of the cohorts (CCCC, InCHIANTI and ULSAM), the sum in these cohorts was calculated as EPA+DHA instead.

Supplementary Table S7. Association of n-3 PUFA biomarkers with incident CKD using a more stringent definition of incident CKD[a]

n-3 PUFA biomarkers	Studies, n[b]	Cases, n[c]	Model[d]	Per interquintile range		Quintile 5 vs. Quintile 1	
				RR (95% CI)	I^2	RR (95% CI)	I^2
**EPA**	16	3397	1	0.95 (0.88 to 1.01)	0.0	0.86 (0.77 to 0.97)	6.1
			2	0.94 (0.87 to 1.00)	0.0	0.84 (0.75 to 0.95)	3.9
**DPA**	13	2931	1	0.94 (0.86 to 1.02)	0.0	0.88 (0.79 to 0.99)	0.0
			2	0.94 (0.86 to 1.02)	0.0	0.89 (0.79 to 1.00)	0.0
**DHA**	16	3399	1	0.91 (0.84 to 0.99)	35.5	0.85 (0.76 to 0.95)	24.0
			2	0.90 (0.83 to 0.98)	23.2	0.85 (0.76 to 0.96)	17.8
**EPA + DPA + DHA[e]**	16	3396	1	0.91 (0.84 to 0.99)	27.3	0.87 (0.77 to 0.97)	30.5
			2	0.90 (0.83 to 0.97)	13.6	0.87 (0.77 to 0.98)	17.7
**ALA**	16	3397	1	1.00 (0.93 to 1.08)	5.1	0.95 (0.85 to 1.07)	0.0
			2	0.99 (0.92 to 1.07)	0.0	0.95 (0.85 to 1.06)	0.0

Abbreviations: CKD, chronic kidney disease; DHA, docosahexaenoic acid; DPA, docosapentaenoic acid; eGFR, estimated glomerular filtration rate; EPA, eicosapentaenoic acid; PUFA, polyunsaturated fatty acids; RR, relative risk.

[a]Incident CKD was defined using more stringent criteria as a follow-up eGFR <60 mL/min/1.73 m^2 and <75% of baseline eGFR among participants with baseline eGFR ≥60 mL/min/1.73 m^2. Effect estimates were pooled using inverse-variance weighted meta-analysis.

[b]Number of cohorts contributing to this analysis was lower than the primary outcome because three cohorts were excluded due to the very low number of incident cases (FDPS, MAS and METSIM).

[c]The small difference in number of cases was due to missing measurement for specific n-3 PUFA fatty acids in some cohorts.

[d]Model 1 adjusted for age, sex, race, clinical centre / field site, education, occupation, body mass index, smoking, alcohol intake, physical activity, prevalent coronary heart disease, and use of lipid-lowering drugs, where applicable. Model 2 adjusted for all covariates in Model 1 and additionally adjusted for prevalent diabetes mellitus, urine albumin-creatinine ratio, systolic blood pressure, use of angiotensin-converting enzyme inhibitors or angiotensin receptor blockers, and use of other anti-hypertensive drugs.

[e]For the sum of seafood n-3 PUFA (EPA+DPA+DHA), as DPA was not available in three of the cohorts (CCCC, InCHIANTI and ULSAM), the sum in these cohorts was calculated as EPA+DHA instead.

Supplementary Table S8. Association of seafood n-3 PUFA biomarkers with incident CKD in sensitivity analyses[a]

Study	EPA		DPA		DHA		EPA + DPA + DHA	
	Model 1	Model 2	Model 1	Model 2	Model 1	Model 2	Model 1	Model 2
Main analysis	0.94 (0.89 to 1.00)	0.94 (0.88 to 0.98)	0.94 (0.88 to 1.00)	0.94 (0.88 to 1.01)	0.93 (0.87 to 1.00)	0.93 (0.87 to 0.99)	0.92 (0.86 to 0.98)	0.91 (0.85 to 0.97)
Analysis after excluding[b]								
AOC	0.94 (0.89 to 1.00)	0.94 (0.89 to 1.00)	0.93 (0.86 to 1.00)	0.94 (0.87 to 1.01)	0.94 (0.88 to 1.01)	0.94 (0.87 to 1.01)	0.92 (0.86 to 0.98)	0.91 (0.85 to 0.98)
ARIC	0.94 (0.89 to 1.00)	0.94 (0.88 to 1.00)	0.94 (0.88 to 1.01)	0.95 (0.89 to 1.02)	0.92 (0.86 to 0.99)	0.91 (0.85 to 0.98)	0.91 (0.85 to 0.98)	0.90 (0.84 to 0.97)
CCCC[c]	0.94 (0.88 to 0.99)	0.93 (0.88 to 0.99)	-	-	0.94 (0.88 to 1.01)	0.93 (0.87 to 1.00)	0.92 (0.86 to 0.98)	0.91 (0.85 to 0.98)
CHS	0.93 (0.88 to 0.99)	0.93 (0.88 to 0.99)	0.94 (0.88 to 1.01)	0.95 (0.89 to 1.02)	0.94 (0.88 to 1.01)	0.94 (0.88 to 1.01)	0.92 (0.86 to 0.99)	0.92 (0.86 to 0.99)
EPIC-Norfolk	0.95 (0.90 to 1.00)	0.94 (0.89 to 1.00)	0.94 (0.88 to 1.00)	0.95 (0.88 to 1.01)	0.94 (0.87 to 1.00)	0.93 (0.87 to 1.00)	0.92 (0.86 to 0.98)	0.92 (0.86 to 0.98)
EPIC-Potsdam	0.94 (0.89 to 1.00)	0.94 (0.89 to 0.99)	0.93 (0.87 to 0.99)	0.94 (0.88 to 1.01)	0.93 (0.87 to 1.00)	0.93 (0.87 to 0.99)	0.92 (0.86 to 0.98)	0.91 (0.85 to 0.97)
FDPS	0.94 (0.89 to 1.00)	0.94 (0.88 to 0.98)	0.94 (0.88 to 1.00)	0.94 (0.88 to 1.01)	0.93 (0.87 to 1.00)	0.93 (0.87 to 0.99)	0.92 (0.86 to 0.98)	0.91 (0.85 to 0.97)
FHS	0.94 (0.89 to 1.00)	0.94 (0.89 to 1.00)	0.93 (0.87 to 1.00)	0.94 (0.88 to 1.01)	0.95 (0.88 to 1.01)	0.94 (0.88 to 1.01)	0.93 (0.87 to 0.99)	0.92 (0.86 to 0.99)
Hisayama	0.94 (0.89 to 1.00)	0.94 (0.88 to 1.00)	0.93 (0.87 to 1.00)	0.94 (0.87 to 1.01)	0.93 (0.86 to 1.00)	0.92 (0.86 to 0.99)	0.91 (0.85 to 0.98)	0.90 (0.84 to 0.97)
InCHIANTI[c]	0.95 (0.90 to 1.00)	0.94 (0.89 to 1.00)	-	-	0.94 (0.88 to 1.01)	0.94 (0.87 to 1.00)	0.93 (0.87 to 0.99)	0.92 (0.86 to 0.98)
KIHD	0.95 (0.89 to 1.00)	0.94 (0.89 to 1.00)	0.94 (0.88 to 1.00)	0.94 (0.88 to 1.01)	0.93 (0.87 to 1.00)	0.92 (0.86 to 0.99)	0.92 (0.86 to 0.98)	0.91 (0.85 to 0.97)
MAS	0.94 (0.89 to 0.99)	0.94 (0.88 to 0.99)	0.93 (0.87 to 0.99)	0.94 (0.88 to 1.00)	0.92 (0.85 to 0.98)	0.91 (0.85 to 0.98)	0.91 (0.85 to 0.97)	0.90 (0.84 to 0.96)
MESA	0.94 (0.89 to 0.99)	0.93 (0.88 to 0.99)	0.94 (0.88 to 1.00)	0.94 (0.88 to 1.01)	0.93 (0.87 to 1.00)	0.93 (0.87 to 1.00)	0.92 (0.86 to 0.98)	0.91 (0.85 to 0.97)
METSIM	0.94 (0.89 to 1.00)	0.94 (0.88 to 0.99)	0.93 (0.87 to 1.00)	0.94 (0.88 to 1.01)	0.93 (0.87 to 1.00)	0.93 (0.87 to 0.99)	0.92 (0.86 to 0.98)	0.91 (0.85 to 0.97)
NHAPC	0.95 (0.90 to 1.01)	0.95 (0.89 to 1.01)	0.93 (0.87 to 1.00)	0.94 (0.88 to 1.01)	0.94 (0.87 to 1.01)	0.93 (0.86 to 1.00)	0.92 (0.86 to 0.99)	0.91 (0.85 to 0.98)
PIVUS	0.94 (0.89 to 1.00)	0.94 (0.89 to 1.00)	0.94 (0.88 to 1.01)	0.95 (0.88 to 1.02)	0.93 (0.86 to 1.00)	0.93 (0.86 to 1.00)	0.91 (0.85 to 0.98)	0.91 (0.85 to 0.98)
ULSAM[c]	0.95 (0.89 to 1.00)	0.94 (0.89 to 1.00)	-	-	0.93 (0.87 to 1.00)	0.93 (0.87 to 1.00)	0.92 (0.86 to 0.98)	0.91 (0.86 to 0.98)
WHIMS	0.94 (0.88 to 0.99)	0.93 (0.88 to 0.99)	0.93 (0.87 to 0.99)	0.93 (0.87 to 1.00)	0.92 (0.86 to 0.99)	0.92 (0.85 to 0.99)	0.91 (0.85 to 0.97)	0.90 (0.84 to 0.96)
3C	0.94 (0.89 to 1.00)	0.94 (0.89 to 0.99)	0.93 (0.87 to 0.99)	0.94 (0.88 to 1.00)	0.93 (0.87 to 0.99)	0.92 (0.86 to 0.99)	0.91 (0.85 to 0.97)	0.91 (0.85 to 0.97)
Alternative biomarker compartment priority[d]	0.94 (0.89 to 0.99)	0.93 (0.88 to 0.99)	0.92 (0.86 to 0.98)	0.93 (0.87 to 0.99)	0.93 (0.87 to 1.00)	0.93 (0.87 to 0.99)	0.91 (0.85 to 0.97)	0.90 (0.84 to 0.96)

Abbreviations: CKD, chronic kidney disease; DHA, docosahexaenoic acid; DPA, docosapentaenoic acid; EPA, eicosapentaenoic acid; PUFA, polyunsaturated fatty acids.

[a]Data are relative risk (95% CI) per interquintile range of each n-3 PUFA biomarker. Incident CKD was defined as an eGFR <60 mL/min/1.73 m^2 during follow-up among participants with baseline eGFR ≥60 mL/min/1.73 m^2. Model 1 adjusted for age, sex, race, clinical centre / field site, education, occupation, body mass index, smoking, alcohol intake, physical activity, prevalent coronary heart disease, and use of lipid-lowering drugs, where applicable. Model 2 adjusted for all covariates in Model 1 and additionally adjusted for prevalent diabetes mellitus, urine albumin-creatinine ratio, systolic blood pressure, use of angiotensin-converting enzyme inhibitors or angiotensin receptor blockers, and use of other anti-hypertensive drugs.

[b]The overall pooled analyses were performed after excluding data from one cohort at a time.

[c]For the sum of seafood n-3 PUFA (EPA+DPA+DHA), as DPA was not available in three of the cohorts (CCCC, InCHIANTI and ULSAM), the sum in these cohorts was calculated as EPA+DHA instead.

[d]For studies evaluating multiple biomarker compartments, results from alternative fractions (i.e., total plasma for 3C instead of erythrocyte phospholipids, and plasma phospholipids for METSIM instead of erythrocyte phospholipids) were utilised in place of results from phospholipids.

Supplementary Table S9. Association of n-3 PUFA biomarkers with incident CKD by study location[a]

Exposure	Region[b]	Studies, n	RR (95% CI), per interquintile range[c]	
			Model 1	Model 2
**ALA**	Europe	11	0.95 (0.86 to 1.05)	0.95 (0.86 to 1.05)
	USA	5	0.97 (0.88 to 1.08)	0.97 (0.87 to 1.07)
	East Asia	3	1.11 (0.98 to 1.25)	1.09 (0.96 to 1.23)
	P value for heterogeneity		0.18	0.25
**EPA**	Europe	11	0.90 (0.82 to 0.99)	0.89 (0.81 to 0.98)
	USA	5	0.99 (0.91 to 1.08)	0.98 (0.90 to 1.07)
	East Asia	3	0.93 (0.83 to 1.04)	0.93 (0.83 to 1.04)
	P value for heterogeneity		0.37	0.35
**DPA**	Europe	9	0.95 (0.86 to 1.06)	0.96 (0.86 to 1.07)
	USA	5	0.90 (0.80 to 1.01)	0.91 (0.81 to 1.02)
	East Asia	2	0.95 (0.84 to 1.08)	0.96 (0.85 to 1.09)
	P value for heterogeneity		0.71	0.80
**DHA**	Europe	11	0.95 (0.86 to 1.06)	0.94 (0.84 to 1.05)
	USA	5	0.91 (0.81 to 1.03)	0.92 (0.81 to 1.03)
	East Asia	3	0.92 (0.81 to 1.05)	0.92 (0.81 to 1.05)
	P value for heterogeneity		0.74	0.77
**EPA + DPA + DHA[d]**	Europe	11	0.92 (0.83 to 1.02)	0.90 (0.81 to 1.01)
	USA	5	0.92 (0.82 to 1.03)	0.92 (0.82 to 1.03)
	East Asia	3	0.91 (0.80 to 1.03)	0.91 (0.80 to 1.04)
	P value for heterogeneity		0.99	1.00

Abbreviations: ALA, α-linolenic acid; CKD, chronic kidney disease; DHA, docosahexaenoic acid; DPA, docosapentaenoic acid; EPA, eicosapentaenoic acid; RR, relative risk.

[a]Data are relative risk (95% CI) per interquintile range of each n-3 PUFA biomarker. Incident CKD was defined as an eGFR <60 mL/min/1.73 m^2 during follow-up among participants with baseline eGFR ≥60 mL/min/1.73 m^2. Effect estimates were pooled using inverse-variance weighted meta-analysis. *P* for heterogeneity across three regions was assessed by meta-regression.

[b]For the MAS cohort, which was conducted in Australia, data were combined with other European cohorts.

[c]Model 1 adjusted for age, sex, race, clinical centre / field site, education, occupation, body mass index, smoking, alcohol intake, physical activity, prevalent coronary heart disease, and use of lipid-lowering drugs, where applicable. Model 2 adjusted for all covariates in Model 1 and additionally adjusted for prevalent diabetes mellitus, urine albumin-creatinine ratio, systolic blood pressure, use of angiotensin-converting enzyme inhibitors or angiotensin receptor blockers, and use of other anti-hypertensive drugs.

[d]For the sum of seafood n-3 PUFA (EPA+DPA+DHA), as DPA was not available in three of the cohorts (CCCC, InCHIANTI and ULSAM), the sum in these cohorts was calculated as EPA+DHA instead.

Supplementary Table S10. Association of n-3 PUFA biomarkers with incident CKD by study follow-up duration[a]

Exposure	Follow-up duration	Studies, n	RR (95% CI), per interquintile range[b]	
			Model 1	Model 2
**ALA**	<median (9 years)	9	0.98 (0.89 to 1.08)	0.98 (0.89 to 1.07)
	≥median (9 years)	10	1.01 (0.93 to 1.10)	1.00 (0.92 to 1.09)
	P value for heterogeneity		0.72	0.72
**EPA**	<median (9 years)	9	0.95 (0.87 to 1.04)	0.95 (0.87 to 1.04)
	≥median (9 years)	10	0.94 (0.87 to 1.00)	0.93 (0.86 to 1.00)
	P value for heterogeneity		0.75	0.70
**DPA**	<median (9 years)	8	0.95 (0.87 to 1.04)	0.95 (0.87 to 1.05)
	≥median (9 years)	8	0.92 (0.84 to 1.01)	0.93 (0.84 to 1.03)
	P value for heterogeneity		0.62	0.72
**DHA**	<median (9 years)	9	0.89 (0.80 to 0.98)	0.88 (0.79 to 0.98)
	≥median (9 years)	10	0.97 (0.89 to 1.06)	0.96 (0.88 to 1.05)
	P value for heterogeneity		0.47	0.52
**EPA + DPA + DHA**[c]	<median (9 years)	9	0.89 (0.80 to 0.99)	0.88 (0.79 to 0.98)
	≥median (9 years)	10	0.94 (0.86 to 1.02)	0.93 (0.86 to 1.01)
	P value for heterogeneity		0.63	0.65

Abbreviations: ALA, α-linolenic acid; CKD, chronic kidney disease; DHA, docosahexaenoic acid; DPA, docosapentaenoic acid; EPA, eicosapentaenoic acid; RR, relative risk.

[a]Data are relative risk (95% CI) per interquintile range of each n-3 PUFA biomarker. Incident CKD was defined as an eGFR <60 mL/min/1.73 m^2 during follow-up among participants with baseline eGFR ≥60 mL/min/1.73 m^2. Effect estimates were pooled using inverse-variance weighted meta-analysis. *P* for heterogeneity across three regions was assessed by meta-regression.

[b]Model 1 adjusted for age, sex, race, clinical centre / field site, education, occupation, body mass index, smoking, alcohol intake, physical activity, prevalent coronary heart disease, and use of lipid-lowering drugs, where applicable. Model 2 adjusted for all covariates in Model 1 and additionally adjusted for prevalent diabetes mellitus, urine albumin-creatinine ratio, systolic blood pressure, use of angiotensin-converting enzyme inhibitors or angiotensin receptor blockers, and use of other anti-hypertensive drugs.

[c]For the sum of seafood n-3 PUFA (EPA+DPA+DHA), as DPA was not available in three of the cohorts (CCCC, InCHIANTI and ULSAM), the sum in these cohorts was calculated as EPA+DHA instead.

Supplementary Table S11. Association of n-3 PUFA biomarkers with incident CKD by study sample size[a]

Exposure	Sample size, n	Studies, n	RR (95% CI), per interquintile range[b] Model 1	Model 2
**ALA**	<median (1082)	9	0.90 (0.80 to 1.01)	0.90 (0.80 to 1.01)
	≥median (1082)	10	1.04 (0.97 to 1.12)	1.03 (0.96 to 1.11)
	P value for heterogeneity		0.05	0.07
**EPA**	<median (1082)	9	0.93 (0.84 to 1.03)	0.92 (0.82 to 1.03)
	≥median (1082)	10	0.95 (0.89 to 1.01)	0.94 (0.88 to 1.01)
	P value for heterogeneity		0.77	0.71
**DPA**	<median (1082)	9	0.97 (0.82 to 1.14)	0.97 (0.82 to 1.15)
	≥median (1082)	10	0.93 (0.86 to 1.00)	0.94 (0.87 to 1.01)
	P value for heterogeneity		0.69	0.75
**DHA**	<median (1082)	9	0.95 (0.84 to 1.09)	0.94 (0.82 to 1.07)
	≥median (1082)	10	0.92 (0.85 to 1.00)	0.92 (0.85 to 1.00)
	P value for heterogeneity		0.69	0.79
**EPA + DPA + DHA**[c]	<median (1082)	9	0.92 (0.81 to 1.04)	0.89 (0.79 to 1.02)
	≥median (1082)	10	0.92 (0.85 to 0.99)	0.92 (0.85 to 0.99)
	P value for heterogeneity		0.99	0.88

Abbreviations: ALA, α-linolenic acid; CKD, chronic kidney disease; DHA, docosahexaenoic acid; DPA, docosapentaenoic acid; EPA, eicosapentaenoic acid; RR, relative risk.

[a]Data are relative risk (95% CI) per interquintile range of each n-3 PUFA biomarker. Incident CKD was defined as an eGFR <60 mL/min/1.73 m^2 during follow-up among participants with baseline eGFR ≥60 mL/min/1.73 m^2. Effect estimates were pooled using inverse-variance weighted meta-analysis. *P* for heterogeneity across three regions was assessed by meta-regression.

[b]Model 1 adjusted for age, sex, race, clinical centre / field site, education, occupation, body mass index, smoking, alcohol intake, physical activity, prevalent coronary heart disease, and use of lipid-lowering drugs, where applicable. Model 2 adjusted for all covariates in Model 1 and additionally adjusted for prevalent diabetes mellitus, urine albumin-creatinine ratio, systolic blood pressure, use of angiotensin-converting enzyme inhibitors or angiotensin receptor blockers, and use of other anti-hypertensive drugs.

[c]For the sum of seafood n-3 PUFA (EPA+DPA+DHA), as DPA was not available in three of the cohorts (CCCC, InCHIANTI and ULSAM), the sum in these cohorts was calculated as EPA+DHA instead.

Supplementary Figure S1. Levels of n-3 PUFA biomarkers across different compartments in the 19 participating cohorts. Fatty acid levels were calculated as percent of total fatty acids, and indicated as median (squares) and interquintile range (lines extending from the squares), respectively. “n” indicates the number of participants. For the sum of seafood n-3 PUFA (EPA+DPA+DHA), as DPA was not available in three of the cohorts (CCCC, InCHIANTI and ULSAM), the sum in these cohorts was calculated as EPA+DHA instead.

(A) α-linolenic acid (ALA; 18:3ω3)

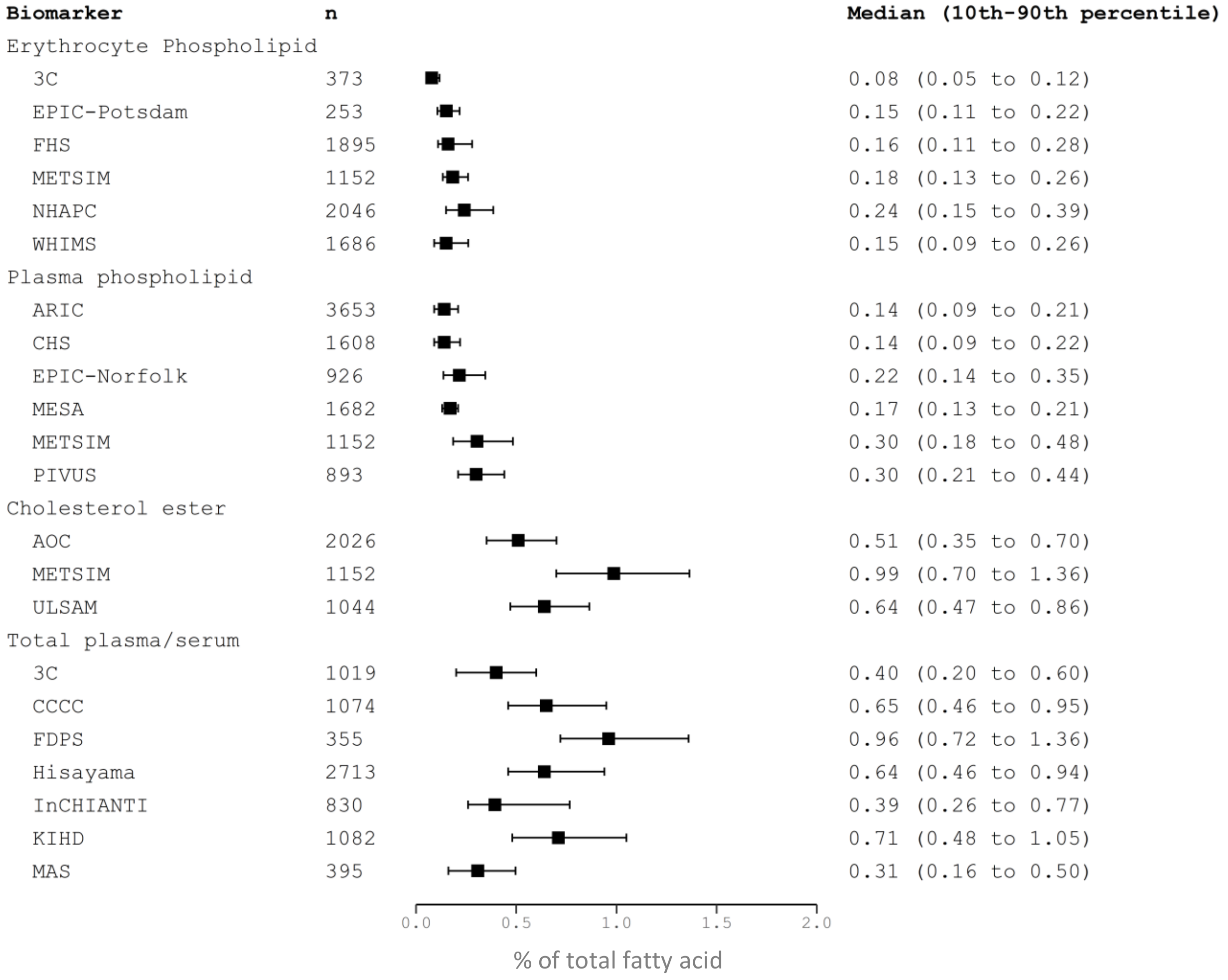

(B) eicosapentaenoic acid (EPA; 20:5ω3)

Biomarker	n	Median (10th-90th percentile)
Erythrocyte Phospholipid		
3C	373	0.72 (0.41 to 1.29)
EPIC-Potsdam	253	0.79 (0.50 to 1.21)
FHS	1895	0.60 (0.37 to 1.13)
METSIM	1152	1.42 (0.90 to 2.30)
NHAPC	2046	0.41 (0.24 to 0.72)
WHIMS	1686	0.63 (0.35 to 1.22)
Plasma phospholipid		
ARIC	3653	0.51 (0.31 to 0.83)
CHS	1608	0.54 (0.32 to 0.97)
EPIC-Norfolk	926	1.06 (0.60 to 2.13)
MESA	1682	0.72 (0.52 to 1.08)
METSIM	1152	2.02 (1.19 to 3.82)
PIVUS	893	1.97 (1.22 to 3.62)
Cholesterol ester		
AOC	2026	1.06 (0.61 to 2.25)
METSIM	1152	2.16 (1.26 to 4.05)
ULSAM	1022	1.25 (0.70 to 2.14)
Total plasma/serum		
3C	1019	0.90 (0.50 to 1.80)
CCCC	1074	0.34 (0.21 to 0.62)
FDPS	355	1.28 (0.69 to 2.73)
Hisayama	2713	2.10 (1.02 to 4.04)
InCHIANTI	830	0.59 (0.42 to 0.84)
KIHD	1082	1.46 (0.89 to 2.65)
MAS	395	1.86 (0.77 to 3.78)

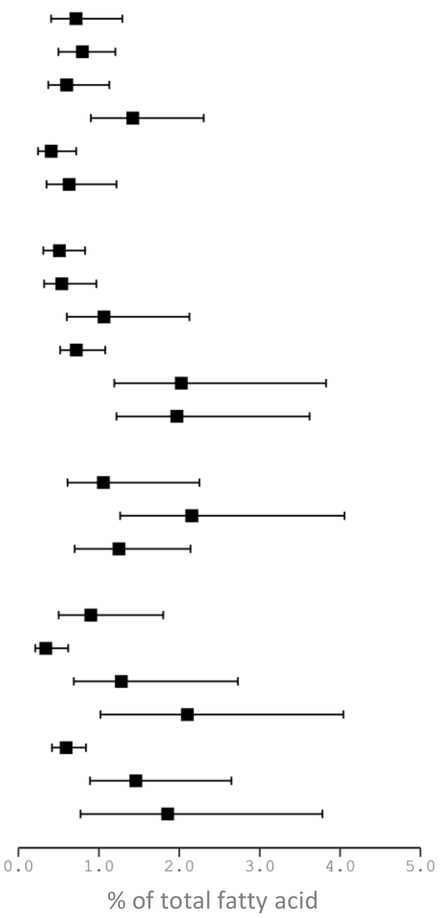

(C) docosapentaenoic acid (DPA; 22:5ω3)

Biomarker	n	Median (10th-90th percentile)
Erythrocyte Phospholipid		
3C	373	1.70 (1.39 to 1.86)
EPIC-Potsdam	253	2.39 (1.83 to 2.84)
FHS	1895	2.67 (2.22 to 3.23)
METSIM	1152	2.59 (2.11 to 3.10)
NHAPC	2046	1.73 (1.39 to 2.13)
WHIMS	1686	2.54 (2.03 to 3.10)
Plasma phospholipid		
ARIC	3653	0.89 (0.69 to 1.12)
CHS	1608	0.83 (0.65 to 1.06)
EPIC-Norfolk	926	1.38 (0.97 to 1.92)
MESA	1682	0.93 (0.81 to 1.08)
METSIM	1152	1.40 (1.15 to 1.67)
PIVUS	893	1.13 (0.88 to 1.42)
Cholesterol ester		
AOC	2026	0.00 (0.00 to 0.08)
Total plasma/serum		
3C	1019	0.50 (0.30 to 0.60)
FDPS	355	0.58 (0.40 to 0.80)
Hisayama	2713	0.62 (0.44 to 0.90)
KIHD	1082	0.55 (0.44 to 0.69)
MAS	395	0.45 (0.17 to 1.59)

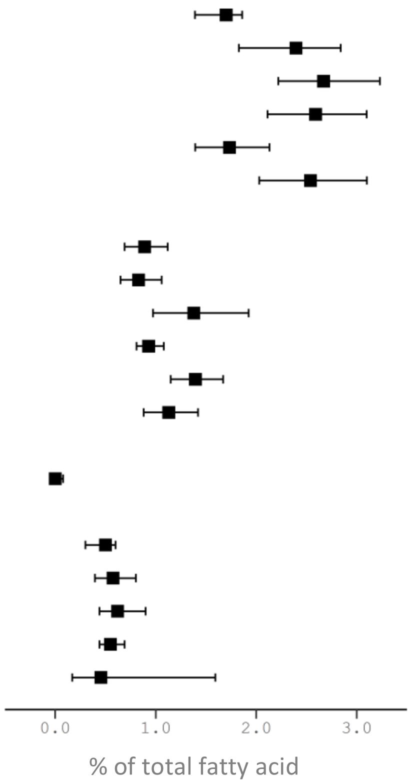

(D) docosahexaenoic acid (DHA; 22:6ω3)

Biomarker	n	Median (10th-90th percentile)
Erythrocyte Phospholipid		
3C	373	4.04 (2.97 to 5.13)
EPIC-Potsdam	253	4.93 (3.50 to 6.21)
FHS	1895	4.62 (3.14 to 6.54)
METSIM	1152	6.21 (4.73 to 7.59)
NHAPC	2046	4.37 (3.21 to 5.77)
WHIMS	1686	4.44 (2.96 to 6.43)
Plasma phospholipid		
ARIC	3653	2.66 (1.87 to 3.93)
CHS	1608	2.93 (1.99 to 4.48)
EPIC-Norfolk	926	5.02 (3.37 to 7.36)
MESA	1682	3.98 (3.03 to 5.14)
METSIM	1152	5.66 (3.84 to 7.63)
PIVUS	893	5.15 (3.78 to 6.81)
Cholesterol ester		
AOC	2026	0.66 (0.44 to 1.00)
METSIM	1152	1.03 (0.70 to 1.49)
ULSAM	1055	0.68 (0.46 to 0.96)
Total plasma/serum		
3C	1019	2.40 (1.60 to 3.50)
CCCC	1074	1.73 (1.09 to 2.65)
FDPS	355	2.75 (1.64 to 4.36)
Hisayama	2713	4.62 (3.24 to 6.52)
InCHIANTI	830	2.26 (1.41 to 3.22)
KIHD	1082	2.38 (1.66 to 3.32)
MAS	395	4.00 (1.29 to 6.81)

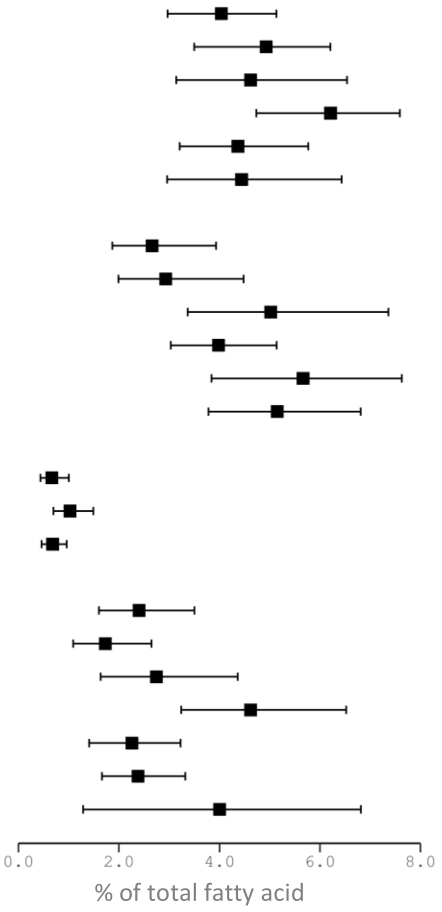

(E) sum of n-3 PUFA (EPA+DPA+DHA)

Biomarker	n	Median (10th-90th percentile)
Erythrocyte Phospholipid		
3C	373	6.42 (5.06 to 8.13)
EPIC-Potsdam	253	8.24 (6.23 to 9.85)
FHS	1895	7.89 (6.14 to 10.51)
METSIM	1152	10.22 (8.10 to 12.60)
NHAPC	2046	6.54 (5.11 to 8.27)
WHIMS	1686	7.65 (5.81 to 10.24)
Plasma phospholipid		
ARIC	3653	3.58 (3.16 to 5.57)
CHS	1608	4.31 (3.25 to 6.23)
EPIC-Norfolk	926	7.56 (3.21 to 11.01)
MESA	1682	5.64 (4.53 to 7.16)
METSIM	1152	9.10 (6.55 to 12.86)
PIVUS	893	8.28 (6.23 to 11.42)
Cholesterol ester		
AOC	2026	1.74 (1.13 to 3.20)
METSIM	1152	3.18 (2.05 to 5.47)
ULSAM	1016	1.93 (1.16 to 3.05)
Total plasma/serum		
3C	1019	3.80 (2.50 to 5.70)
CCCC	1074	2.10 (1.36 to 3.22)
FDPS	355	4.55 (2.88 to 7.66)
Hisayama	2713	7.37 (4.83 to 11.17)
InCHIANTI	830	2.87 (1.91 to 4.02)
KIHD	1082	4.33 (3.14 to 6.60)
MAS	395	6.54 (3.09 to 11.78)

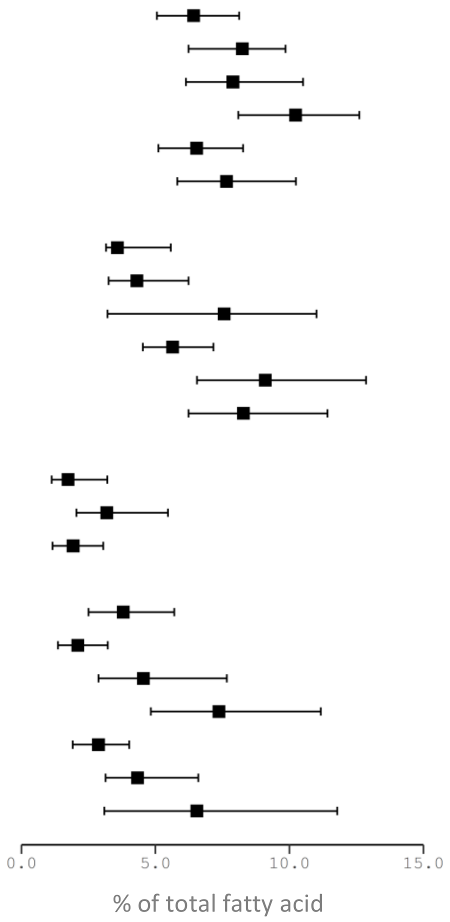

Supplementary Figure S2. Association of quintiles of seafood n-3 PUFA biomarkers with incident CKD. Incident CKD was defined as an eGFR <60 mL/min/1.73 m^2 during follow-up among participants with baseline eGFR ≥60 mL/min/1.73 m^2. Analyses were adjusted for the following covariates: age, sex, race, field centre if applicable, education, occupation, body mass index, smoking, physical activity, alcohol intake, prevalent coronary heart disease, and use of lipid-lowering drugs. See Table 1 footnote for abbreviations of cohorts. Abbreviations: CKD, chronic kidney disease; DHA, docosahexaenoic acid; DPA, docosapentaenoic acid; eGFR, estimated glomerular filtration rate; EPA, eicosapentaenoic acid; RR, relative risk; Q, quintile.

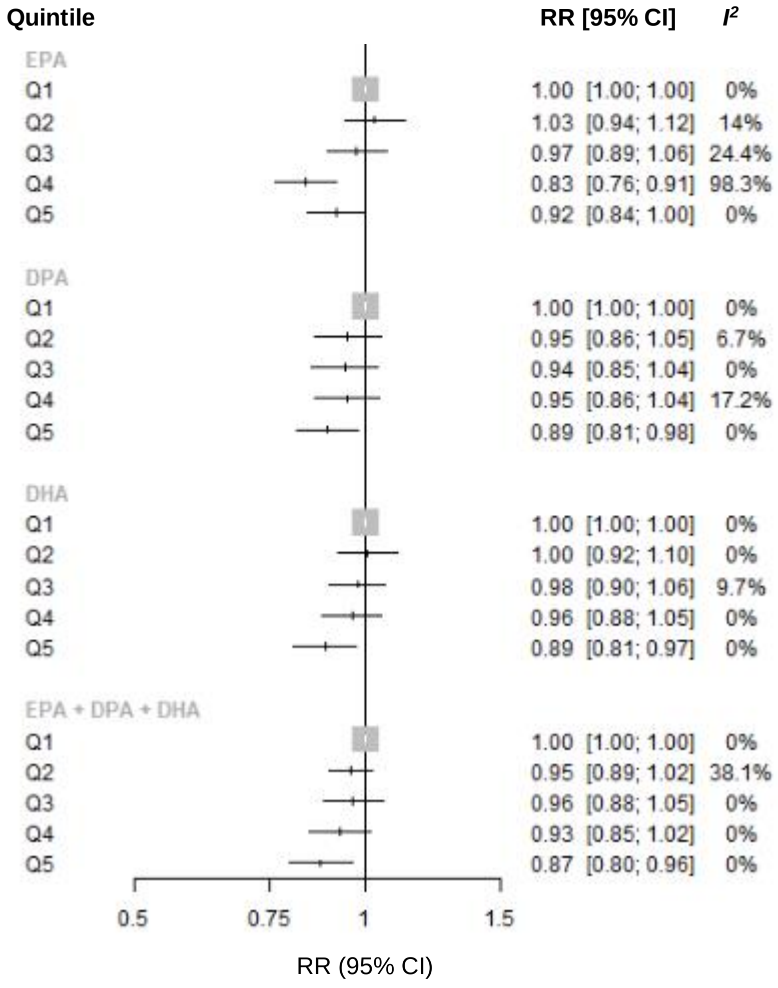

Supplementary Figure S3. Relationship between total seafood n-3 PUFA (EPA+DPA+DHA) and risk of incident CKD assessed using restricted cubic splines. Associations were evaluated using multivariate meta-analysis with 3-knot restricted cubic splines, for each of the biomarker compartments. The reference value was set at the 10th percentile of each fatty acid-biomarker exposure. The 95% confidence interval is depicted as the area between the dashed lines.

(A) Erythrocyte phospholipids (6 cohorts)

P-nonlinearity = 0.84

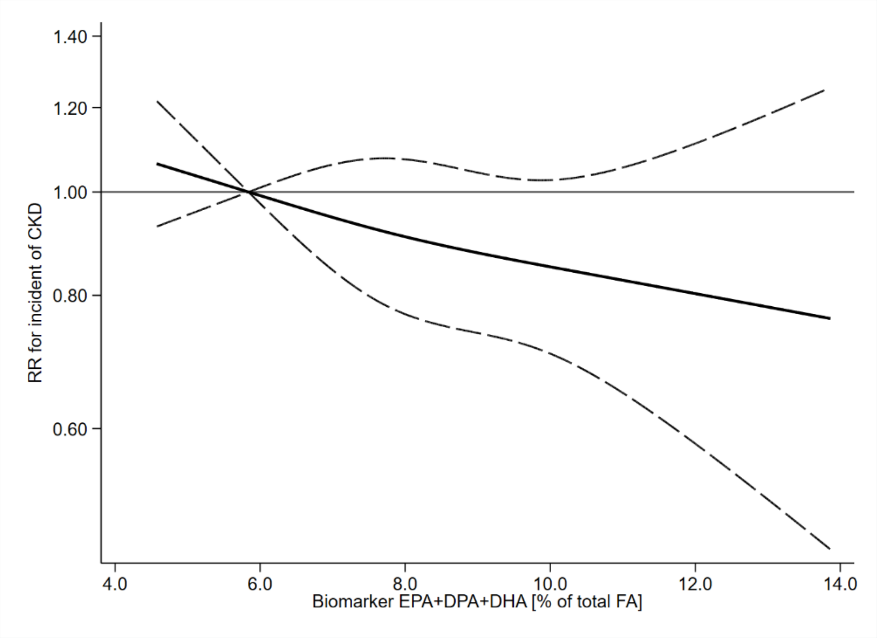

(B) Plasma phospholipids (6 cohorts)

P-nonlinearity = 0.71

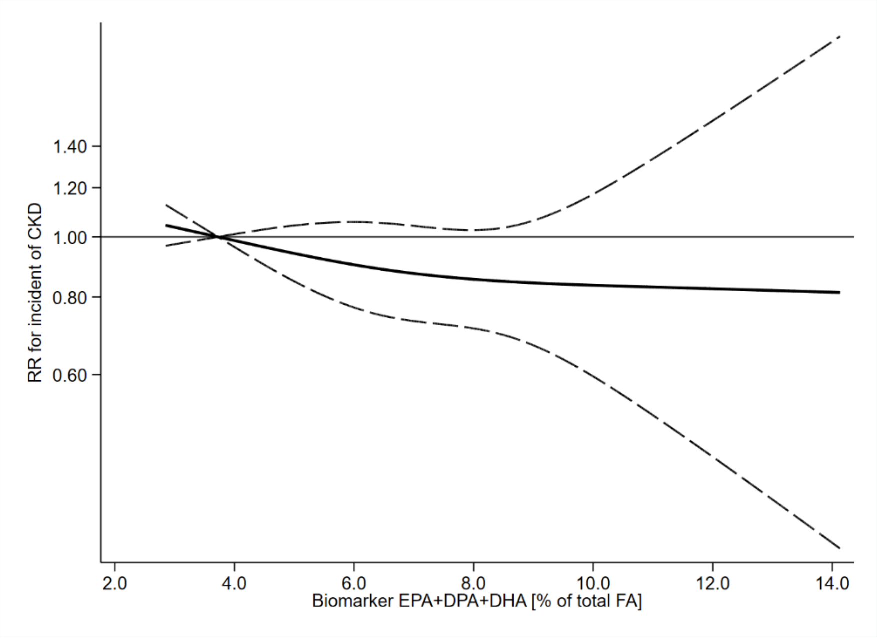

(C) Cholesterol esters (3 cohorts)

P-nonlinearity = 0.42

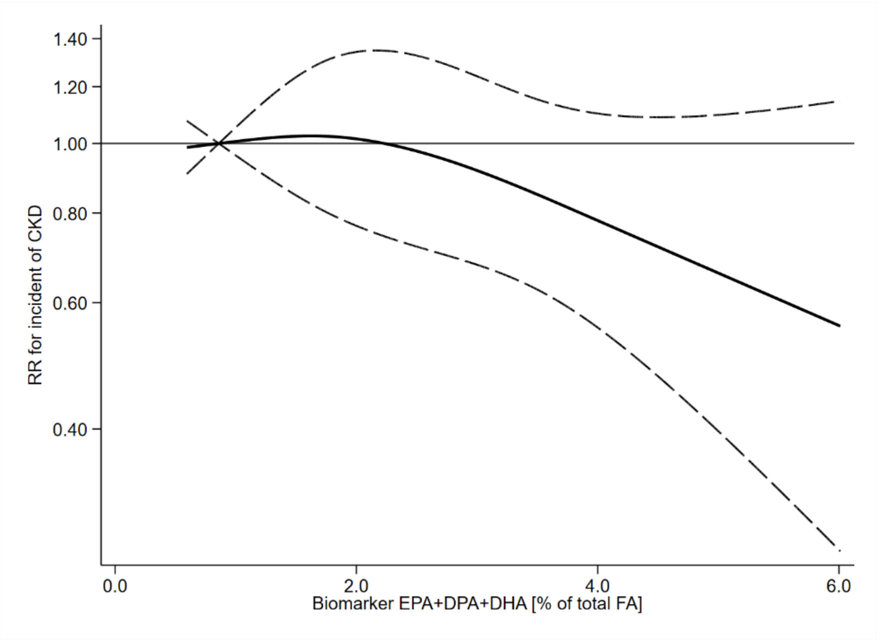

(D) Total plasma/serum (6 cohorts)

P-nonlinearity = 0.14

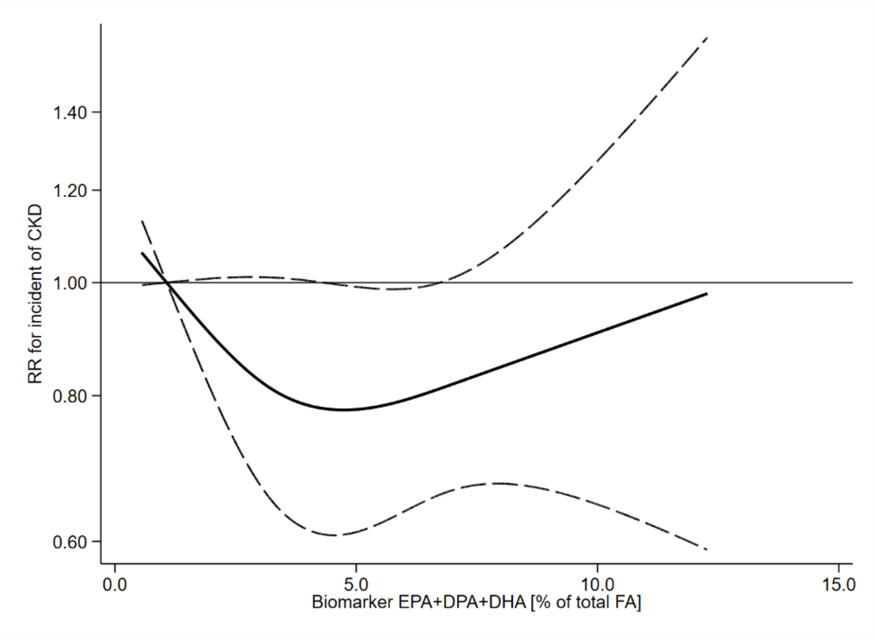

Supplementary Figure S4. Association of α-linolenic acid with incident CKD. Incident CKD was defined as an eGFR <60 mL/min/1.73 m^2 during follow-up among participants with baseline eGFR ≥60 mL/min/1.73 m^2. Analyses were adjusted for the following covariates: age, sex, race, field centre if applicable, education, occupation, body mass index, smoking, physical activity, alcohol intake, prevalent coronary heart disease, and use of lipid-lowering drugs. See Table 1 footnote for abbreviations of cohorts. Abbreviations: RR, relative risk; Q, quintile.

(A) Continuous analysis (per interquintile range)

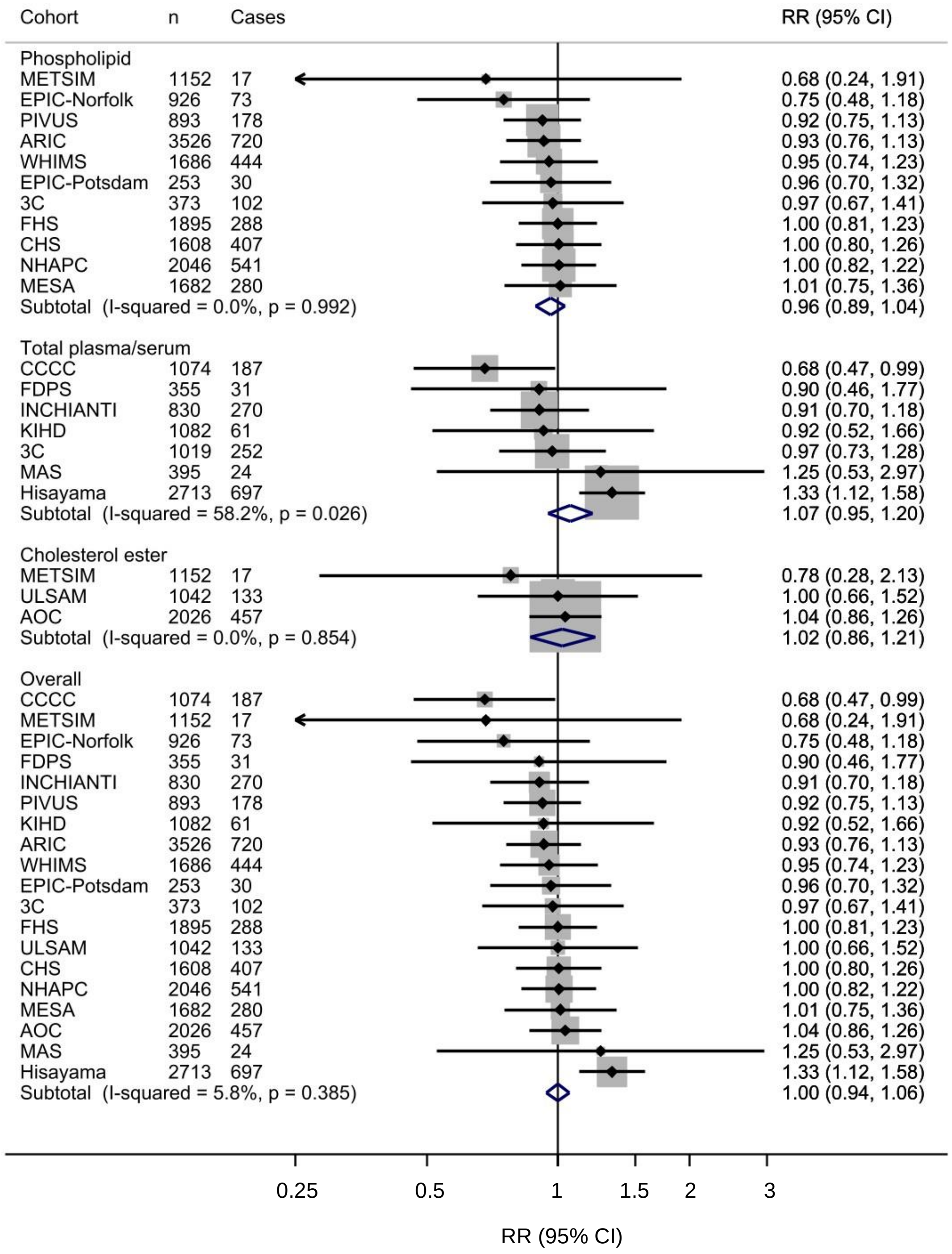

(B) Categorical analysis (quintiles)

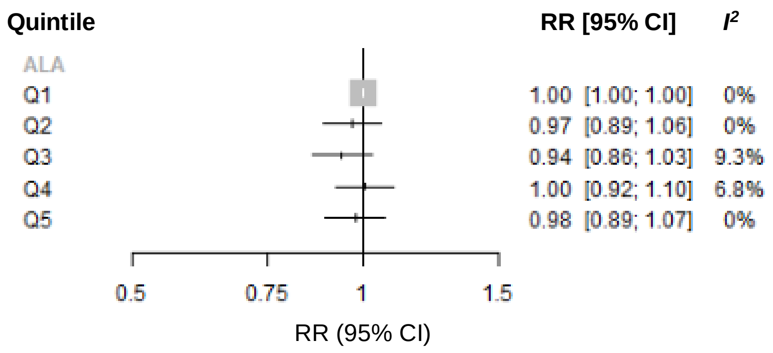

Supplement: Supplementary file 1 — Web appendix: Supplementary online content [file ongk072909.ww.pdf]
